# UMIB Summit 2015

**DOI:** 10.1097/MD.0000000000002371

**Published:** 2016-03-11

## UMIB SUMMIT PROCEEDINGS

Editorial – Mariana P. Monteiro.

UMIB Summit 2015 was the first international scientific event organized by the Unit for Multidisciplinary Research in Biomedicine. Being a research unit based at a medical school, UMIB's research interests span across a broad field of medical sciences and specialties. Therefore, organizing a multidisciplinary translational research event in an era of increasing specialization, was definitely a big challenge that UMIB decided to undertake. The three host institutions of UMIB, the Instituto de Ciências Biomédicas Abel Salazar of University of Porto, the Santo António Hospital and the Jacinto Magalhães Center of Medical Genetics, both belonging to the Centro Hospitalar do Porto, have gathered their efforts to organize this meeting.

Our aim was not only to present UMIB's latest achievements, but also to bring clinicians and researchers from across different fields in medical sciences together, towards improved advances in the biomedical knowledge and human healthcare. The UMIB Summit has been designed to disseminate UMIB's work by providing an overall view of the active research lines of the unit and of our peer researchers. The event was intended to promote synergies not only within UMIB's research groups, as well as, with other national and international research institutions, some were already ongoing or were emerging collaborations and partnerships. The UMIB Summit was a truly international event focusing on biomedicine, which during the two full days had 270 registered delegates, coming from 14 different countries across Europe, Africa, America and Asia. The program included a plenary lecture on “How to establish successful translational research protocols”, by Inmaculada Ibáñez Cáceres from IdiPaz Madrid, which illustrated the example of role model institution dedicated to translational research. There were 7 thematic symposia, organized by the principal investigators of UMIB's research groups, focused on the Immune response to infection, Genetic and neurodegenerative diseases, Mechanisms of cancer treatment resistance, Autoimmune and inflammatory mechanisms, Dysmetabolism and Chronic Kidney Disease, Gastrointestinal Hormones and type 2 Diabetes, and Human Fertility and Reproductive disorders, in which the latest breakthroughs in science were presented. In addition, over 60 oral and 40 posters were presented, distributed by seven different categories, and among these, a panel nominated by the scientific Committee elected the awardees for each category. The past few years have been challenging times for scientific research units and for UMIB in particular, as a result of the well-known nationwide financial constraints. Nevertheless, we strive to continue our consolidation as research unit while remaining adaptable and open to innovative ideas, and the overall success of the UMIB SUMMIT will most certainly contribute to attain these aims.

## SYMPOSIA ABSTRACTS

## IMMUNITY AND INFLAMMATION

### S01 – Immune response in the adipose tissue

Luzia Teixeira, PhD. Unit for Multidisciplinary Research in Biomedicine, Institute of Biomedical Sciences, University of Porto (UMIB, ICBAS-UP) (e-mail: lmteixeira@icbas.up.pt)

The adipose tissue has been increasingly recognized as an organ not only involved in energy homeostasis but also contributing to immune responses. Indeed, inflammation in adipose tissue has been associated with human metabolic disorders. Moreover, it has been shown that the adipose tissue is a reservoir for diverse microorganisms and studies focusing on the immune response to infection in this tissue are arising. In recent years we have been characterizing the immune response elicited in the adipose tissue upon *Neospora caninum* infection, an abortive parasite closely related to *Toxoplasma gondii*. We showed that infection established with a single challenge with this parasite contributes to marked immune cell alterations in the adipose tissue still observed at a chronic phase of infection. Moreover, leptin levels were increased in chronically infected animals suggesting that persistent metabolic alterations may be elicited by infection in the lean host.

**Acknowledgements:** LT is supported by Fundo Social Europeu and Programa Operacional Potencial Humano through FCT Investigator Grant IF/01241/2014.

### S02 – Human Immunodeficiency as a research tool

Ana Espada Sousa, MD, PhD. Instituto de Medicina Molecular, Faculdade de Medicina, Universidade de Lisboa (IMM, FMUL) (e-mail: asousa@medicina.ulisboa.pt)

HIV infection induces a generalized immune activation that is the main determinant of the progressive CD4 T cell depletion that ultimately leads to AIDS in the absence of antiretroviral treatment. HIV-2, like HIV-1, establishes a disseminated infection with persistent viral reservoirs and immune activation. Nevertheless, the plasma viral load is usually undetectable and the CD4 decline rate is very slow in HIV-2-infected individuals. We have been exploring the distinct equilibria established between the host and HIV-1 and HIV-2, not only to better understand HIV immunopathogenesis per se, but also to identify the host's immune system mechanisms to counteract CD4 depletion and limit immunopathology. Moreover, within the scope of our primary immunodeficiency centre, we have been able to combine the provision of differentiated diagnosis and follow-up with the generation of unique knowledge through the study of these naturally-occurring human gene knock-out settings.

### S03 – Dendritic cells: central players in orchestration of type 2 inflammation

Andrew S. Macdonald, PhD. Manchester Collaborative Centre for Inflammation Research, United Kingdom (e-mail: andrew.macdonald@manchester.ac.uk)

Dendritic cells (DCs) are specialised innate immune cells that play a key role in initiation and direction of adaptive immunity against diverse immune challenges. However, relatively little is known about precisely how DCs become activated and function in Type 2 settings, either during parasitic helminth infection or following exposure to allergens. We have shown that DCs responding to helminths display an unusual, low level, activation phenotype. Irrespective of this, we have demonstrated that DCs are both sufficient and necessary for induction of Type 2 immunity against several helminth species. Although DCs are clearly centrally involved in coordination of the immune response during Type 2 inflammation, the specific DC subsets that are required, and the mechanism(s) by which they direct Th2 polarisation, remain poorly understood. Our recent work addressing these fundamental issues will be presented.

## GENETIC AND NEURODEGENERATIVE DISORDERS

### S04 – Alzheimer's disease biomarkers in mouse models of cerebral b-amyloidosis: bridging the translational gap

Luís F. Maia, MD, PhD. Hospital de Santo António, Centro Hospitalar do Porto (HSA-CHP), Portugal (e-mail: luis.lf.maia@gmail.com)

Abnormalities in brains of Alzheimer's Disease (AD) patients start long before the first clinical symptoms. Identifying individuals at this “preclinical AD” stage relies on biochemical and imaging biomarkers. However, little is known on the longitudinal biomarker dynamics, particularly in the earliest preclinical stages. Transgenic mice overexpressing human amyloid precursor protein (APP) are excellent models for Aβ pathology, but remained largely unexplored in the study of CSF AD biomarkers. Using APP-tg mice, we showed that CSF Aβ and t-Tau follow the trends predicted to occur in AD patients. CSF Aβ reflected brain Aβ-pathological changes and CSF t-Tau seems a marker of Aβ pathology progression at later stages. When we tracked CSF Aβ to earlier time points, we detected a transient increase of CSF Aβ that pinpointed the emergence of the first amyloid plaques. Our findings support the translational use of APP-tg in the search for novel disease biomarkers and may open new perspectives in identifying and stratifying subjects at risk for AD significantly earlier, for preventive treatment strategies.

### S05 – Neurodevelopmental disorders: many genetic causes pointing towards common mechanisms of disease

Hans van Bokhoven, PhD. Molecular Neurogenetics Unit, Departments of Human Genetics and Cognitive Neuroscience, Radboud University Medical Center, Donders Institute for Brain, Cognition and Behaviour, Nijmegen, Netherlands (e-mail: Hans.vanBokhoven@radboudumc.nl)

Neurodevelopmental disorders (NDDs) constitute a heterogeneous group of disorders for which the genetic foundations are rapidly being uncovered. The large number of NDD-associated gene mutations presents an opportunity to identify common mechanisms of disease as well as molecular processes that are of key importance to normal and abnormal development and function of the human brain. Shared molecular networks may be affecting specific neurobiological processes that are spatio-temporally restricted in their activity. Accordingly, mutations in genes of such networks usually give rise to specific brain anomalies and/or cognitive dysfunction. Other networks may have more widespread biological functions, which is reflected in the clinical manifestations associated with mutations in components of these networks. I will discuss examples of both sorts of gene networks underlying NDDs and the implications for further research into strategies for therapeutic intervention.

### S06 – EuroBioBank: A collaborative, transnational biobanking network for rare diseases

Marina Mora, PhD. Muscle Cell Biology Lab, Neuromuscular Diseases and Neuroimmunolgy Unit, Fondazione Istituto Neurologico C. Besta, Milano, Italy (e-mail: mmora@istituto-besta.it)

The EuroBioBank (EBB) network was the first network of biobanks in Europe dedicated to Rare Disease (RD) research. It was established in 2001 under the auspices of two patient organisations and was funded by the European Commission (5th framework programme). The network set up and consolidation phases have brought cohesion to the network, coordination of activities, development of a quality control system and expansion of the network through the joining of new partners. It enlists RD biobanks from eight EU and three other countries with a catalogue of over 350,000 samples. EBB has been involved in the European Biobanking and Biomolecular Resources Research Infrastructure planning and, recently, has become partner of RD-Connect, an FP7 EU program aimed at linking RD biobanks, registries and bioinformatics data. In this context EBB contributes with expertise, and promotes high professional standards and best practices, thus challenging the fragmentation of international cooperation on the field.

## MECHANISMS OF CANCER TREATMENT RESISTANCE

### S07 – Mechanisms of resistance to treatment in metastatic castration resistant prostate cancer

António Araújo, MD, PhD. Service of Medical Oncology, Centro Hospitalar do Porto - Unit for Multidisciplinary Research in Biomedicine, Institute of Biomedical Sciences University of Porto (UMIB, ICBAS-UP) (e-mail: antonio.araujo@chporto.min-saude.pt)

Prostate cancer (PC) is the second leading cause of cancer death in men. The androgen deprivation therapy (ADT) is the standard treatment for patients diagnosed at advanced stages, since the androgens can stimulate the growth, survival and inhibit apoptosis of normal and tumoral prostate cells. However these responses typically last 18 to 24 months, after which the patients develops resistance to ADT and castration-resistant prostate cancer (CRPC). CRPC is an uncertain and a lethal stage of the disease, with limited treatments and with a strong propensity to spread to the bone. Several molecular mechanisms were responsible for PC cells survival after ADT but they are not entirely understood. Presently, it is assumed that AR signalling resurgence activity is a key for therapeutic failure and CRPC development. Several ligand-dependent and independent mechanisms have been proposed to underlie AR signalling reactivation, causing changes in cell AR-regulated transcriptome, affecting the expression of genes involved in cell proliferation and death, immune system and DNA repair. In conclusion, there is a clinical need for more specific, sensitive and accurate cancer biomarkers to assist clinicians in the patients’ management and the analysis of AR-regulated genes expression profiles in circulating samples, such as blood, could be a more precise strategy in patients’ management.

### S08 – Mechanisms of resistance in breast cancer

Luís Costa, MD, PhD. Hospital de Santa Maria, Instituto de Medicina Molecular (IMM), Lisbon, Portugal (e-mail: luiscosta.oncology@gmail.com)

In the past 5 years the major discoveries in the medical treatment of breast cancer (BC) happened in the field of how to circumvent treatment resistance. The applied scientific translational research provided significant insight about the mechanisms of resistance in endocrine therapy and in HER2 target therapy in BC. Until now there no major advances to evade resistance to chemotherapy in BC. Endocrine therapy (ET) is a major option either in palliative or in adjuvant setting for about 60% of BC cases. In the metastatic setting (palliative) we often provide 2 or 3 lines of ET and the rate of clinical benefit in first line is about 50%, decreasing afterwards. In Bolero2 study, the use of the mTOR inhibitor everolimus in combination with exemestane in second-line of ET, was associated with a significant increase in the time to progression of disease, as if those patients were treated as first-line ET. In our own data, pS6 (a biomarker of mTOR activity) correlates with survival in metastatic BC patients. Another relevant target to revert (or prevent) resistance of ET in BC is the inhibition of CDK4/6. Palbociclib is a CDK4 inhibitor that increases the response and time to progression in metastic BC when added to the aromatase inhibitor letrozole. The second most frequent biological target in BC is HER2. For those BC patients with overexpression of HER2 trastuzumab is a fundamental part of the treatment and changed the natural history of the disease either in adjuvant or in palliative setting. However, mechanisms of resistance to trastuzumab were well described and are now new targets to treat BC. In the Cleopatra study, Pertuzumab in combination with trastuzumab and docetaxel was significantly superior to docetaxel and trastuzumab alone. The advantage of including Pertuzumab in the therapeutic strategy increased in about 15 months the median overall survival for metastatic BC patients. Another significant step in HER2 BC disease was observed when T-DM1 was tested in EMILIA study for trastuzumab resistant disease. This new form of conducting chemotherapy to the cancer cell using trastuzumab was significantly superior to the previous alternative of combining lapatinib with capecitabine. We are living enthusiastic times in the new era of reverting or preventing resistance in metastatic BC. However, we do not know yet if these options will increase the rate of cure in adjuvant setting.

### S09 – Exosomes and drug resistance: the new frontier of liquid biopsy

Christian Rolfo, MD, PhD. Phase I – Early Clinical Trials Unit, Antwerp University Hospital, Center for Oncological Research (CORE), Antwerp University, Belgium (e-mail: Christian.Rolfo@uza.be)

Drug resistance plays a crucial role especially in tumor therapy, where the most part of resistance mechanisms against therapeutic agents remain unclear. Tumor derived exosomes (TDEs) have a strong role. Exosomes have pleiotropic effects in physiological and pathological conditions. One studied mechanism is drug exportation via exosome pathway. In human ovarian carcinoma cell lines it was demonstrated that exosomes released from cisplatin-resistant cells contained more cisplatinum than exosomes released from cisplatin-sensitive cells, suggesting that this mechanism can be exploited by cancer cell to export drugs. Moreover, it was described that exosomes can also function to neutralize antibody-based drugs, overexpressing target proteins (i.e. HER2) in order to decrease the antibody amount that could bind parental cancer cells. In addition, was also proposed that drugs or their intracellular metabolites can be expelled out of the cells through exosomal mechanisms mediated by ABC transport. Another example is prostate cancer, exosomal transfer of multi drug resistance proteins as MDR-1/P-gp involved in docetaxel resistance. These findings strongly suggest that exosomes releasing and drug resistance are connected in several tumors. More preclinical studies are required in order to support this hypothesis of exosomes-mediated drug resistance.

## AUTOIMMUNE AND INFLAMMATORY MECHANISMS

### S10 – Autoimmunity Network

Carlos Vasconcelos, MD, PhD. Unit for Multidisciplinary Research in Biomedicine, Institute of Biomedical Sciences University of Porto (UMIB, ICBAS-UP) Hospital de Santo António, Centro Hospitalar do Porto (HSA-CHP) (e-mail: cvcarlosvasconcelos@gmail.com)

The immune system develops its action in a network of humoral factors, cells and territoires with the objective of maintaining homeostasis. The specifity of immune responses goes side-by-side with degeneracy and redundancy. Heteroimmune with autoimmune responses. Physiological autoimmune and heteroimmune responses may evolve to pathological autoimmunity. Why this happens is still unknown. Autoimmune diseases are becoming an important health system problem, as more than 10 % of the population may be affected by one these diseases. The commonest are organ-specific, as those involving thyroid, but virtually all organs and biological systems can be involved, being the systemic ones, like Lupus, those more clinically problematic. Autoimmune disease is also described as a network, as the presence of one increases the probability of the occurrence of another, resulting in polyautoimmunity. Autoimmune diseases and immunodeficiencies are networked as the first can be described as an immunodeficiency of the physiological homeostatic network and many patients have infections, even without the influence of exogenous immunosupressors; by other side autoimmune manifestations are the second commonest in immunodeficiency patients. Network of clinical centres dedicated to autoimmune diseases are of utmost importance for pushing joint efforts in order to develop the scientific field and better treat patients.

### S11 – Tissue damage control in immune mediated inflammatory diseases

Miguel Soares, PhD. Instituto Gulbenkian de Ciência, Lisbon, Portugal (IGC) (e-mail: mpsoares@igc.gulbenkian.pt)

Damage control is a concept used for example by the naval industry to refer to emergency procedures put in place when dealing with situations that may cause the sinking of a ship. Damage control has also been used to refer to a company in Marvel Comics, which specializes in repairing damaged property arising from conflicts between superheroes and supervillains. I will use tissue damage control to refer to a biologic concept in which adaptive responses to different types of stress act in a concerted manner to limit the extent of tissue damage arising in the context of infection. I will argue that tissue damage control is an underlying mechanism of disease tolerance, a defense strategy that limits the severity of infectious diseases, irrespectively of the host pathogen load. I will propose that tissue damage control might be integrated as an inherent component of immunity that decouples inflammation and immunity from tissue damage and disease.

### S12 – Epigenetics in autoimmunity and inflammation

Esteban Ballestar, PhD. Institut d’Investigació Biomèdica de Bellvitge (IDIBELL), Barcelona, Spain (e-mail: eballestar@idibell.cat)

Epigenetics, including DNA methylation, plays a fundamental role in differentiation and function of cells by driving and stabilising gene activity states. Our group aims at understanding the functional role, targeting mechanisms, and interplay of the enzymes involved in deposition or active removal of 5-methylcytosine with transcription factors and upstream signalling pathways in differentiation processes in the immune system. Analysis of these elements provides clues on how they can be misregulated in autoimmune and autoinflammatory diseases. In fact, DNA methylation alterations do take place in autoimmune diseases like rheumatoid arthritis (RA) and systemic lupus erythematosus (SLE). This presentation will be divided in two parts: in the first part, I will discuss the latest findings on alterations in DNA methylation, autoimmune diseases and inflammatory processes. During the second part, I will present some of our findings and the current status of the field on targeting mechanisms for DNA methylation changes and their connections with extracellular signals.

## DYSMETABOLISM AND CHRONIC KIDNEY DISEASE

### S13 – Glucose, obesity and insulin resistance in dialysis

Ana Paula Bernardo, MD, Anabela Rodrigues, MD. Unit for Multidisciplinary Research in Biomedicine, Institute of Biomedical Sciences University of Porto (UMIB, ICBAS-UP) and Nephrology Department, Hospital de Santo António, Centro Hospitalar do Porto (HSA-CHP) (e-mail: anabernardo@portugalmail.pt)

Cumulative exposure to glucose, used as an osmotic agent in Peritoneal Dialysis solutions, is presumed to induce systemic hyperglycemia, obesity, and aggravate insulin-resistance. Peritoneal membrane characteristics (fast transport status) and therapy schedules might also condition glucose absorption. But evidence is still lacking about the causative role of peritoneal glucose absorption on body fat accumulation over time. Therefore we explored the leptin/adiponectin ratio (LAR) and homeostasis model assessment corrected for adiponectin (HOMA-AD) in nondiabetic peritoneal dialysis patients, as better correlations of glucose-disposal rate. We concluded that insulin resistance is associated with obesity and LAR independently of glucose absorption and small-solute transport status. Fast transport status was not associated with obesity or insulin resistance. Current PD treatments minimize glucose exposition. The lack of correlations between peritoneal glucose absorption and insulin resistance/body composition parameters suggest that other factors contribute to a metabolic syndrome development in PD patients. Beyond genetics, resting energy expenditure, energy intake and the role of physical exercise deserve investigation in CKD patients.

### S14 – Pancreas-kidney transplantation in diabetic patients: metabolic and immunologic outcomes

Jorge Malheiro, MD, La Salete Martins, MD, PhD. Unit for Multidisciplinary Research in Biomedicine, Institute of Biomedical Sciences University of Porto (UMIB, ICBAS-UP) and Nephrology & Kidney Transplantation Unit, Hospital de Santo António, Centro Hospitalar do Porto (HSA-CHP) (e-mail: jjorgemalheiro@gmail.com)

Simultaneous pancreas-kidney transplantation (SPKT) has been recommended by ADA as the treatment of choice in type 1 DM patients with CKD, given its favorable impact on patient survival. From May 2000 to October 2012, 150 type 1 diabetic patients underwent SKPT in our center. This presentation will revue our program results in terms of significant adverse post-transplant events, cardiovascular and metabolic outcomes, grafts and patient survival. We will also present our investigational data on the impact of pancreatic autoimmunity and *de novo* allorecognition in SPKT. Persistence or relapse of pancreatic autoantibodies was associated with worse glycemic control but not with pancreas graft failure. *De novo* DSA had an independent detrimental effect on pancreas graft survival, while, in kidney graft, its negative effect on survival was related with acute rejection occurrence. We believe that prospective analysis of auto- and allo-immunity should have a role in the management of SPKT recipients.

### S15 – Transcriptomics to identify novel mediators of renal cell death

Alberto Ortiz, MD, PhD. IIS – Fundación Jiménez Díaz, Madrid, Spain (e-mail: Aortiz@fjd.es)

Chronic kidney disease is the fastest growing non-transmissible global cause of death. This portrays our inability to adequately treat kidney disease, due to our insufficient understanding of pathogenesis. –omics techniques have been studied or the non-biased identification of novel mediators of kidney disease. We have used kidney transcriptomics to identify upregulation of the TWEAK receptor FN14 and then went on to characterize a key role of TWEAK/FN14 in kidney injury, including acute kidney injury, chronic interstitial fibrosis and non-immune proteinuric nephropathies. Of interest, TWEAK downregulates the kidney expression of the antiaging factor Klotho, which was also found to be downregulated during kidney injury by kidney transcriptomics. TWEAK is a potent inducer of apoptotic and necroptotic renal cell death under inflammatory circumstances. Anti-TWEAK neutralizing antibodies are currently undergoing clinical trials for lupus nephritis.

## GASTROINTESTINAL HORMONES AND TYPE 2 DIABETES

### S16 – Insights from bariatric surgery into metabolic control

Mariana Monteiro, MD, PhD. Unit for Multidisciplinary Research in Biomedicine, Institute of Biomedical Sciences University of Porto (UMIB, ICBAS-UP) (e-mail: mpmonteiro@icbas.up.pt)

Bariatric surgery has an important antidiabetic effect in obese type 2 diabetes patients. Possible mechanisms to explain bariatric surgery induced hypoglycemic effect and metabolic improvement include weight loss, decreased food intake, decreased insulin resistance and changes in gut hormone secretion profile derived from the anatomical rearrangement of the gastro-intestinal tract. Bariatric surgery affects the levels of several hormones, such as, GLP-1, oxyntomodulin, PYY and ghrelin, which signal satiety and hunger to the human brain and improve metabolic control. In particular, the early arrival of undigested nutrients to the distal intestine can lead to overstimulation of GLP-1 secreting L cells, which predominate 200 cm distally to the Treitz ligament, to enhance the incretin effect and promote insulin secretion. Thus, the metabolic benefits of bariatric surgery could potentially be maximized by selecting the procedure with the most suitable endocrine profile to target the patient comorbidities and specially type 2 diabetes.

### S17 – Design of high-affinity gip receptor ligands

Lærke Smidt Hansen, MD, Alexander Hovard Sparre-Ulrich, cand. Scient, PhD, Mikkel Christensen, MD, PhD, Filip Krag Knop, MD, PhD, Jens Juul Holst, MD, PhD, Mette Marie Rosenkilde, MD, PhD. University of Copenhagen, Denmark (e-mail: rosenkilde@sund.ku.dk)

GIP (Glucose-dependent Insulinotropic Polypeptide) is an intestinal hormone secreted in response to a meal. The GIP receptor belongs to 7-transmembrane receptor family B and is located in the pancreas, brain, bone, cardiovascular system, GI-tract and adipose tissue. GIP stimulates insulin and glucagon secretion glucose-dependently and combined with adipogenic effects, GIP are given increased attention in relation to diabetes and obesity. Several aspects of the GIP pharmacology are influential. Firstly, there are considerable differences between rodent and human receptors which impacts basic and translational research. Secondly, a mutation in GIP that creates degradation resistance (Pro3(GIP)) increases the half-life but decreases affinity and efficacy. Furthermore the effect of this mutation is diverse between species. Thirdly, truncated GIP variants with high affinity inhibit receptor signaling length-dependently, whereas a naturally occuring C-terminal truncation improves antagonism. The peptide based antagonists are tolerated well *in vivo* and will be tools for exploration of GIP physiology and pathophysiology.

### S18 – The future of glp-1 based therapy

Jens Juul Holst, MD, PhD. NNF Center for Basic Metabolic Research and Department of Biomedical Sciences, University of Copenhagen, Denmark (e-mail: jjholst@sund.ku.dk)

GLP-1 based therapies include GLP-1 receptor agonists and inhibitors of the enzyme, dipeptidyl peptidase-4, which rapidly degrades GLP-1; the inhibitors therefore increase plasma levels of endogenous GLP-1. The inhibitors are available as once daily (and soon once weekly) oral tablets and lower HbA1c levels (0.5–1.0 %) in most patients with insignificant side effects. They were safe in large outcome trials and may have protective microvascular effects. GLP-1 receptor agonists are injectable but are available in short-acting (hours) and long-acting forms (days -> weeks), provide greater improvements in A1c, and also reduce food intake causing weight loss, but may also have usually short-lasting and mild gastrointestinal side effects. An oral GLP-1 agonist is under development. Their action is limited in patients with poor beta-cell function, but together with basal insulin most patients (70–80%) will reach treatment goals. The inhibitors, which will be increasingly cheaper, will probably, combined with metformin, substitute SUs. Also, combinations with SGLT-2 inhibitors (if they survive) may be beneficial. The GLP-1 agonists will be used in oral treatment failures particularly in combination with basal insulin (a combination which is superior to intensified, multiple dose insulin therapy). They may also be used in obesity because they prevent diabetes development in high risk individuals, whereas application for obesity alone probably will be successful only if combinations with other anti-obesity agents are effective and if the safety profile remains benign.

## HUMAN FERTILITY AND REPRODUCTIVE DISORDERS

### S19 – Oocyte dysmorphisms and female fertility

Rosália Sá, PhD. Department of Microscopy, Laboratory of Cell Biology, Institute of Biomedical Sciences Abel Salazar (ICBAS), University of Porto (UP); Unit for Multidisciplinary Research in Biomedicine (UMIB-UP) (e-mail: rmsa@icbas.up.pt)

Scientific evidence indicates that oocyte quality is a crucial limiting factor of female fertility. About 70% of the mature oocytes retrieved during assisted reproduction cycles exhibit one or more abnormal morphologic abnormalities. Oocyte dysmorphisms can be extracytoplasmic, intracytoplasmic, or both. Of these dysmorphisms, only a few have been strongly correlated with negative embryological and clinical outcomes (giant oocyte, small oocyte, vacuolization, large aggregates of smooth endoplasmic reticulum tubules, granular vacuoles, large refractile body, dense or irregular zona pellucida, small or large polar body). At the present date, none of these dysmorphisms have been correlated to a gene defect, and despite intensive research on oocyte proteomics and metabolomics, only a few promising results have been obtained. Thus, the detailed inspection of these dysmorphisms remains the most robust mean to predict the oocyte fertilizing ability and developmental competence.

**Acknowledgements:** UMIB (Pest-OE/SAU/UI0215/2014) was funded by National Funds through FCT-Foundation for Science and Technology.

### S20 – Diabetes mellitus: a threat for male fertility?

Pedro F. Oliveira, PhD. Department of Microscopy, Laboratory of Cell Biology, Institute of Biomedical Sciences Abel Salazar (ICBAS) and Institute of Health Research and Innovation (I3S), University of Porto (e-mail: pfobox@gmail.com)

Diabetes mellitus (DM) is a major public health threat in modern societies. Although it was suggested for many years that DM did not have a significant effect on male reproductive function, this has been challenged by recent findings. Compelling evidence suggests that the metabolic and hormonal deregulation associated with this disease compromises male reproductive potential by promoting profound alterations in testicular metabolism, particularly in the metabolism of somatic Sertoli cells (SCs). Spermatogenesis depends on SCs glycolytic metabolism, which is markedly altered during the progressive stages of DM. The more pronounced effects were reported when DM was established, while in the prodromal stage of DM, testicular metabolism tends to adapt in order to meet developing germ cells’ demands. Nevertheless, in all cases, these metabolic changes were associated with a decrease in sperm quality, which will surely be accountable for the decline observed in male reproductive health.

### S21 – Male late-onset hypogonadism: current concepts and controversies

Ilpo Huhtaniemi, MD, PhD, MD(hc), FMedSci. Institute of Reproductive and Developmental Biology, Department of Surgery & Cancer, Imperial College London, Hammersmith Campus, UK (e-mail: ilpo.huhtaniemi@imperial.ac.uk)

A recent fashion is to diagnose ageing men with borderline suppressed testosterone and diffuse symptoms, such as impaired sexual function, physical fatigue and depression, as having late-onset hypogonadism (LOH), and offering them testosterone replacement therapy for treatment. Testosterone treatment of LOH occurs in the ‘off-label’ domain, and the reputed beneficial effects and lack of adverse events are mainly based on experience and subjective feelings of the patients and treating physicians, in the absence of evidence-based data. This speaker has been involved for the last 15 years in the European Male Ageing Study (EMAS), a prospective multicenter cohort study on endocrine and metabolic changes in men upon ageing. Some recent findings of the EMAS study will be reported in this lecture.

## ORAL COMMUNICATIONS AND POSTERS ABSTRACTS

## BIOLOGY OF REPRODUCTION

### CO1 – Signaling fingerprint of the human spermatozoa: correlation with clinical data

Joana V. Silva, MSc^1^, Maria João Freitas, MSc^1^, Bárbara R. Correia, BSc^1^, Luís Korrodi-Gregório, PhD^1^, António Patrício, MD^2^, Steven Pelech, PhD^3,4^, Margarida Fardilha, PhD^1^. ^1^Laboratory of Signal Transduction, Institute for Research in Biomedicine – iBiMED, Health Sciences Program, University of Aveiro, Campus Universitário de Santiago, 3810-193, Aveiro, Portugal, ^2^Hospital Infante D. Pedro E.P.E., 3810-193 Aveiro, Portugal, ^3^Kinexus Bioinformatics Corporation, Vancouver, British Columbia, Canada V6P 6T3, ^4^Department of Medicine, University of British Columbia, Vancouver, British Columbia, Canada V6P 6T3 (e-mail: mfardilha@ua.pt)

Sperm cells are virtually incapable of genetic expression and thus highly dependent upon post-translational modifications and signal cascades to execute their function. In this study we attempted to unravel the signaling pathways involved in regulating human sperm function and to correlate the activity of signaling proteins with clinical data. A total of 37 human semen samples, obtained from a randomized group of donors, were included in this study. Basic semen parameters were analyzed according to the WHO's guidelines. Sperm DNA fragmentation (SDF) was measured using a Sperm Chromatin Dispersion (SCD) test. Antibody-based arrays were carried out to determine the expression patterns of 18 well-characterized signaling molecules when phosphorylated or cleaved. A commercial Kinetworksô Protein Kinase Screen was used to analyze the levels of 75 protein kinases. The results indicated that the phosphorylated levels of several proteins [Bad, GSK-3β, HSP27, JNK/SAPK, mTOR, p38 MAPK and p53], as well as, cleavage of PARP (at D214), and Caspase-3 (at D175)] were significantly correlated with motility parameters. Additionally, the percentage of morphologically normal spermatozoa demonstrated a significant positive correlation with the phosphorylated levels of p70 S6 kinase and, in turn, head defects and the teratozoospermia index (TZI) showed a significant negative correlation with the phosphorylated levels of Stat3. There was a significant positive correlation between SDF and the TZI, as well as, the presence of head defects. In contrast, SDF negatively correlated with the percentage of morphologically normal spermatozoa and the phosphorylation of Akt and p70 S6 kinase. Subjects with varicocele demonstrated a significant negative correlation between head morphological defects and the phosphorylated levels of Akt, GSK3β, p38 MAPK and Stat1. Additionally, 34 protein kinases were identified as expressed in their total protein levels in normozoospermic samples. From those, 8 protein kinases were identified for the first time in human spermatozoa. This study contributed towards establishing a biomarker “fingerprint” to assess sperm quality based on molecular parameters.

**Acknowledgements:** This work was supported by: Research grant from the Portuguese Urology Association (APU); “FCT – Fundação para a Ciência e Tecnologia (PTDC/DTP-PIC/0460/2012) and cofinanced by FEDER through “Eixo I do Programa Operacional Fatores de Competitividade (POFC) do QREN” (COMPETE: FCOMP-01-0124-FEDER-028692); and individual fellowship from FCT to JVS (SFRH/BD/81458/2011).

### CO2 – New insights into the regulation of human Sertoli cells metabolism: differential effects of dehydroepiandrosterone and 7-oxo-dehydroepiandrosterone

Tânia R. Dias, MSc^1^, Marco G. Alves, PhD^1^, Susana P. Almeida, MSc^2^, Joaquina Silva, MD^3^, Alberto Barros, MD, PhD^3,4,5^, Mário Sousa, MD, PhD^2,3^, Branca M. Silva, PhD^1^, Samuel M. Silvestre, PhD^1,6^, Pedro F. Oliveira, PhD^2,5^. ^1^CICS – UBI – Health Sciences Research Centre, University of Beira Interior, 6200-506 Covilhã, Portugal, ^2^Department of Microscopy, Laboratory of Cell Biology and Unit for Multidisciplinary Research in Biomedicine (UMIB), Institute of Biomedical Sciences Abel Salazar (ICBAS), University of Porto, 4050-313 Porto, Portugal, ^3^Centre for Reproductive Genetics Prof. Alberto Barros, 4100-009 Porto, Portugal, ^4^Department of Genetics, Faculty of Medicine, University of Porto, 4050-313 Porto, Portugal, ^5^Institute of Health Research and Innovation (I3S), University of Porto, 4050-313 Porto, Portugal (e-mail: pfobox@gmail.com)

Dehydroepiandrosterone (DHEA) is a precursor of about 30–50% of androgens in adult men. DHEA action is partially exerted through its metabolites. Within these, 7-oxo-dehydroepiandrosterone (7-oxo-DHEA) is a biological DHEA metabolite that is not convertible to androgens and has proven to be a promising therapeutic agent. The process of spermatogenesis, central to male fertility, is strongly regulated by hormones, including androgens. Sertoli cells (SCs) constitute the main support of spermatogenesis. They present high metabolic rates to ensure lactate production for the developing germ cells, which may lead to high oxidative stress. Since any disruptions in androgen synthesis may compromise SCs function and male fertility, we aimed to evaluate the effects of DHEA and 7-oxo-DHEA in human SCs (hSCs) metabolism and oxidative profile. hSCs were exposed to increasing concentrations of DHEA and 7-oxo-DHEA (0.025, 1 and 50 μM) that revealed to be non-cytotoxic in these experimental conditions. The consumption/production of metabolic substrates by hSCs were measured by ^1^H-NMR. Additionally, protein levels of key players of hSCs glycolytic pathway were evaluated by Western blot. The levels of protein oxidation and nitration, as well as lipid peroxidation were measured by Slot blot to evaluate hSCs oxidative status. The obtained data demonstrated that 7-oxo-DHEA is a more potent metabolic modulator than DHEA since it increased hSCs glycolytic flux. DHEA seems to redirect hSCs metabolism to the Krebs cycle, while 7-oxo-DHEA has some inhibitory effect in this pathway. The highest 7-oxo-DHEA concentrations (1 and 50 μM) also increased lactate production, which is of extreme relevance for the successful progression of spermatogenesis *in vivo*. None of these steroids significantly altered the intracellular oxidative profile of hSCs, illustrating that the concentrations used do not have pro- nor antioxidant actions in hSCs. Overall, we found that DHEA and 7-oxo-DHEA induce distinct alterations in hSCs metabolism, which transduce the respective androgenic and non-androgenic effects of these steroids. The exposure of hSCs to DHEA seems to stimulate the Krebs cycle, while exposure to 7-oxo-DHEA increased hSCs glycolytic flux. Our study represents a further step in the establishment of safe doses of DHEA and 7-oxo-DHEA to hSCs, supporting its possible use in hormonal and non-hormonal therapies against male reproductive problems.

**Acknowledgements:** This work was supported by “Fundação para a Ciência e a Tecnologia” – FCT to Tânia R. Dias (SFRH/BD/109284/2015); Marco G. Alves (SFRH/BPD/80451/2011 and PTDC/BIM-MET/4712/2014); Samuel M. Silvestre (SFRH/BPD/41612/2007); Pedro F. Oliveira (SFRH/BPD/108837/2015 and PTDC/BBB-BQB/1368/2014); CICS (PEst-C/SAU/UI0709/2014) and UMIB (PEst-OE/SAU/UI0215/2014) co-funded by Fundo Europeu de Desenvolvimento Regional - FEDER via Programa Operacional Factores de Competitividade - COMPETE/QREN.

### CO3 – The antidiabetic drug Pioglitazone alters human Sertoli cells metabolism by increasing its glycolytic efficiency

Maria J. Meneses, MSc^1,2^, Raquel L. Bernardino, MSc^1^, Rosália Sá, PhD^1^, Joaquina Silva, MD^3^, Alberto Barros, MD, PhD^3,4,5^, Mário Sousa, MD, PhD^1,3^, Branca M. Silva, PhD^2^, Pedro F. Oliveira, PhD^1,5^, Marco G. Alves, PhD^2^. ^1^Department of Microscopy, Laboratory of Cell Biology and Unit for Multidisciplinary Research in Biomedicine (UMIB), Abel Salazar Institute of Biomedical Sciences (ICBAS), University of Porto, Porto, Portugal, ^2^CICS-UBI – Health Sciences Research Centre, University of Beira Interior, Covilhã, Portugal, ^3^Centre for Reproductive Genetics Professor Alberto Barros, Porto, Portugal, ^4^Department of Genetics, Faculty of Medicine, University of Porto, Porto, Portugal, ^5^Institute of Health Research and Innovation (I3S), University of Porto, Porto, Portugal (e-mail: alvesmarc@gmail.com)

Pioglitazone is a potent synthetic agonist for the nuclear receptor peroxisome proliferator-activated receptor γ used, alone or in combination with metformin, to treat type 2 Diabetes *mellitus* (T2DM). In recent years, new clinical benefits have been described to Pioglitazone but its effects on male reproductive system have not been investigated though other antidiabetic drugs, such as metformin, were reported to regulate the nutritional support of spermatogenesis by Sertoli cells (SCs). Thus, we proposed to study human SCs (hSCs) metabolism after exposure to pioglitazone alone or in combination with metformin. We hypothesized that Pioglitazone can act as a modulator of hSCs metabolism, altering the nutritional support of spermatogenesis. To test our hypothesis, hSCs were cultured in the presence of pioglitazone (1, 10, 100 μM) and pioglitazone 1.5 μM plus metformin (10 μM). hSCs were obtained from testicular biopsies from six men under treatment for recovery of male gametes and presenting anejaculation (psychological, vascular, neurologic) and conserved spermatogenesis. Protein levels of glycolysis-related enzymes and transporters were determined by Western blot. Lactate dehydrogenase activity was spectrophotometrically assessed. Mitochondrial complexes levels were studied by Western blot and mitochondrial membrane potential was studied with a dye (JC-1). Finally, metabolite production and consumption were determined by ^1^H-NMR. The suprapharmacological concentration of pioglitazone increased glucose consumption and mitochondrial complex II protein levels in hSCs though mitochondrial membrane potential was decreased. On the other hand, the pharmacological concentration of pioglitazone (10 μM) stimulated lactate production and allowed the establishment of important correlations among several key intervenient of glycolysis in hSCs. Treatment of hSCs with pioglitazone and metformin did not alter the glycolytic profile of these cells. Nevertheless, it induced alterations in the expression of mitochondrial complexes III and V in hSCs. Overall, our results provide clear evidence that the pharmacological dose of pioglitazone (10 μM) increases the efficiency of hSCs glycolytic flux. This led to an increase in lactate production, which is known to improve spermatogenesis. Thus, pioglitazone might be considered a suitable antidiabetic drug for men in reproductive age and with potential to improve male fertility.

**Acknowledgements:** This work was supported by the “Fundação para a Ciência e a Tecnologia”–FCT co-funded by Fundo Europeu de Desenvolvimento Regional - FEDER via Programa Operacional Factores de Competitividade - COMPETE/QREN to UMIB (PEst-OE/SAU/UI0215/2014); CICS-UBI (Pest-C/SAU/UI0709/2014); RL Bernardino (SFRH/BD/103105/2014); PF Oliveira (PTDC/BBB-BQB/1368/2014 and SFRH/BPD/108837/2015) and MG Alves (SFRH/BPD/80451/2011 and PTDC/BIM-MET/4712/2014).

### CO4 – Mammalian target of rapamycin is a novel modulator of Sertoli cells glycolytic and oxidative profiles

Tito T. Jesus, MSc^1,2^, Pedro F. Oliveira, PhD^1,3^, Joaquina Silva, MD^4^, Alberto Barros, MD, PhD^3,4,5^, Rita Ferreira, PhD^6^, Mário Sousa, MD, PhD^1,4^, C. Yan Cheng, PhD^7^, Branca M. Silva, PhD^2^, Marco G. Alves, PhD^2^. ^1^Department of Microscopy, Laboratory of Cell Biology, Unit for Multidisciplinary Research in Biomedicine (UMIB), Abel Salazar Institute of Biomedical Sciences (ICBAS), University of Porto, Porto, Portugal, ^2^CICS-UBI – Health Sciences Research Centre, University of Beira Interior, Covilhã, Portugal, ^3^Institute of Health Research and Innovation (I3S), University of Porto, Porto, Portugal, ^4^Center for Reproductive Genetics Professor Alberto Barros, Porto, Portugal, ^5^Faculty of Medicine (FMUP), University of Porto, Porto, Portugal, ^6^QOPNA, Department of Chemistry, University of Aveiro, Aveiro, Portugal, ^7^Population Council's Center for Biomedical Research, New York, USA (e-mail: alvesmarc@gmail.com)

Sertoli cells (SCs) are testicular somatic cells present in the seminiferous tubule epithelium. They are responsible for the physical and nutritional support of developing germ cells. The mammalian target of rapamycin (mTOR) is a conserved serine/threonine kinase known to be involved in several cellular functions, including metabolism which is pivotal in testis. In this study, we hypothesized that mTOR regulates the nutritional support of spermatogenesis and the oxidative profile of human SCs. Human SCs were treated with rapamycin, an allosteric inhibitor of mTORC1. Their glycolytic profile was assessed by Proton Nuclear Magnetic Resonance and by studying the protein expression of key glycolysis-related transporters and enzymes. The expression of mitochondrial complexes was determined, protein carbonylation and nitration as well as lipid peroxidation were quantified to establish an oxidative profile. mTOR signaling pathway was also studied. We demonstrate that exposure of human SCs to rapamycin increases glucose consumption although maintaining the production of lactate. Thus, inhibition of mTORC1 did not increase the glycolytic flux of SCs which are usually reported to possess a Warburg-like metabolic behavior. Alanine production by rapamycin-exposed cells was severely affected, resulting on an unbalanced intracellular redox state, as noted by the lactate/alanine ratio. This redox unbalance resulted in an increase of lipid peroxidation in rapamycin-exposed human SCs. Rapamycin treatment also resulted in decreased expression of mitochondrial complex III and decreased expression of the phosphorylated-mTOR at Ser-2448 evidencing an effective partial inhibition of mTORC1. Nevertheless, the protein levels of the downstream signaling molecule of mTORC1, p-4EB-P1, were not altered suggesting that during the 24 hours treatment it is rephosphorylated. Our data give new insight to a crucial role for mTOR in male reproduction, particularly by regulating the nutritional support of spermatogenesis by human SCs.

**Acknowledgements:** This work was supported by the Portuguese “Fundação para a Ciência e a Tecnologia” - FCT: Tito T. Jesus (SFRH/BD/103518/2014); Marco G. Alves; (SFRH/BPD/80451/2011 and PTDC/BIM-MET/4712/2014); Pedro F. Oliveira; (SFRH/BPD/108837/2015); CICS-UBI (Pest-C/SAU/UI0709/2014); UMIB (Pest-OE/SAU/UI0215/2014) and co-funded by FEDER via Programa Operacional Fatores de Competitividade-COMPETE/QREN & FSE and POPH funds.

### CO5 – Protective effects of N-acetylcysteine on human spermatozoa DNA during short-term etoposide exposure *in vitro*

A Rabaça, BSc^1,2,3^, A Gonçalves, BSc^4^, C Ferreira, BSc^1,2,5^, R L Bernardino, MSc^1,2^, A Barros, MD, PhD^4,6,7^, M G Alves, PhD^8^, M Sousa, MD, PhD^1,2,4^, P F Oliveira, PhD^1,2,8^, R Sá, PhD^1,2^. ^1^Department of Microscopy, Laboratory of Cell Biology, Institute of Biomedical Sciences Abel Salazar (ICBAS), University of Porto, 4050-313 Porto, Portugal, ^2^Unit for Multidisciplinary Research (UMIB), University of Porto, 4050-313 Porto, Portugal, ^3^Faculty of Medicine, University of Porto (FMUP), 4200-319 Porto, Portugal, ^4^Centre for Reproductive Genetics Prof. Alberto Barros, 4100-009 Porto, Portugal, ^5^University of Trás-os-Montes and Alto Douro (UTAD), 5000-801 Vila Real, Portugal, ^6^Department of Genetics, Faculty of Medicine, University of Porto, 4200-319 Porto, Portugal, ^7^Institute of Health Research and Innovation, University of Porto, Portugal, ^8^Health Sciences Research Centre (CICS), University of Beira Interior (UBI), 6201-506 Covilhã, Portugal (e-mail: rmsa@icbas.up.pt)

Male fertility can be affected by several commonly used chemotherapeutics like etoposide. Etoposide is a topoisomerase II inhibitor responsible for the induction of permanent double-stranded DNA breaks that affects spermatogenesis and impairs fertility recovery after treatment. Therefore, there is a need to preserve male fertility during etoposide exposure. N-acetylcysteine (NAC) has chemopreventive and antioxidant properties displaying several protective effects on cells. Besides, NAC improves semen parameters from oxidative stress-induced damages due to its antioxidant potential. As NAC has been reported to possess cytoprotector properties, we theorised that it may be a good conserver of sperm quality during etoposide exposure. Human spermatozoa were incubated for 2 h at 37 °C with 25 μg/ml of etoposide, 50 μM of NAC and a combination of both drugs. Spermatozoa motility, vitality and morphology, as well as DNA fragmentation and chromatin condensation were evaluated. Oxidative damages were measured and sperm metabolism was studied by proton nuclear magnetic resonance spectroscopy (^1^H-NMR). Results demonstrate that short-term exposure of human spermatozoa to etoposide *in vitro* induces chromatin alterations and DNA fragmentation. Moreover, etoposide does not induce sperm oxidative damages nor glycolytic profile alterations. NAC's addition to sperm exposed to etoposide preserved sperm chromatin condensation and reduced sperm DNA fragmentation (sDNAfrag). In conclusion, although short-term exposure to etoposide does not affect sperm vitality, it induces severe chromatin alterations and DNA damages. NAC addition to etoposide-exposed sperm protected chromatin integrity and highly reduced sDNAfrag. Thus, NAC acts as a cytoprotector, sheltering spermatozoa DNA from the etoposide-induced damages. Additionally, exposure of spermatozoa to etoposide does not induce cellular oxidative damages nor glycolytic profile alterations, providing evidence that etoposide directly affects DNA. NAC's ability to preserve sperm DNA is clinically relevant, as the majority of patients undergoing chemotherapy fail to cryopreserve semen prior to the initiation of treatment. Consequently, the integrity of the collected spermatozoa after etoposide treatment initiation could be preserved by NAC's addition, guaranteeing that the majority of viable sperm would have their DNA integrity conserved and could be securely used in assisted reproductive technologies.

**Acknowledgements:** UMIB (Pest-OE/SAU/UI0215/2014) and MG Alves (SFRH/BPD/80451/2011) were funded by National Funds through FCT-Foundation for Science and Technology and A Rabaça by the Master Course in Molecular Medicine and Oncology, FMUP.

### CO6 – Metformin preserves human sperm DNA integrity during short-term incubation

C Ferreira, BSc^1,2,3^, A Gonçalves, BSc^4^, A Rabaça, BSc^1,2,5^, R L Bernardino, MSc^1,2^, A Barros, MD, PhD^4,6,7^, PF Oliveira, PhD^1,7^, M Sousa, MD, PhD^1,2,4^, MG Alves, PhD^8^, R Sá, PhD^1,2^. ^1^Department of Microscopy, Laboratory of Cell Biology, Institute of Biomedical Sciences Abel Salazar (ICBAS), University of Porto, 4050-313 Porto, Portugal, ^2^Unit for Multidisciplinary Research in Biomedicine (UMIB), University of Porto, 4050-313 Porto, Portugal, ^3^University of Trás-os-Montes and Alto Douro (UTAD), 5000-801 Vila Real, Portugal, ^4^Centre for Reproductive Genetics Prof. Alberto Barros (CGR), 4100-009 Porto, Portugal, ^5^Faculty of Medicine, University of Porto (FMUP), 4200-319 Porto, Portugal, ^6^Department of Genetics, Faculty of Medicine, University of Porto, 4200-319 Porto, Portugal, ^7^Institute of Health Research and Innovation, University of Porto, Portugal, ^8^Health Sciences Research Centre (CICS), University of Beira Interior (UBI), 6201-506 Covilhã, Portugal (e-mail: rmsa@icbas.up.pt)

Metformin, an oral antidiabetic agent, is the most cost-effective therapy used for the treatment of patients with type 2 Diabetes mellitus. Although this medicine has been clinically used for 50 years, its exact mechanism of action remains poorly understood. Recently, other applications for metformin have emerged such as the treatment of reproductive pathologies. Nonetheless, its effects on human male reproduction, namely in sperm, remain scarcely known. Male infertility due to abnormal semen parameters has become a treatable problem by the use of assisted reproductive technologies (ART). Sperm preparation methods enable the purification of high quality sperm, but sperm DNA integrity and motility not always can be totally improved. As metformin has been reported to possess antioxidant properties and act as a metabolic modulator, we hypothesized if this agent could be a good additive to sperm media during the short-term incubation used in ART treatments. Human sperm samples were obtained from 12 male patients at CGR, and were incubated for 2 h at 37°C in sperm preparation medium supplemented with increasing concentrations of metformin. We tested sub-pharmacological (5 μM), pharmacological (50 μM) and supra-pharmacological (500 μM) metformin concentrations. Sperm parameters, oxidative damages (protein carbonylation and nitration; lipid peroxidation), metabolism (proton nuclear magnetic resonance spectroscopy, ^1^H-NMR), and DNA integrity were assessed. None of the metformin concentrations had an effect on human sperm oxidative profile. Incubation of human sperm during 2 h, increased sperm DNA fragmentation (sDNAfrag). At 5 μM, metformin decreased acetate production. At 50 μM, metformin decreased sperm motility, with high levels of choline production, and increased immature chromatin condensation and the sperm glycolytic flux. On the other hand, at 500 μM, metformin reduced sDNAfrag and immature chromatin condensation, to values found at collection time, increased sperm motility, and did not altered the sperm glycolytic profile. Results revealed that at supra-pharmacological concentrations, metformin is able to preserve sperm quality and DNA integrity of morphologically normal sperm, thus indicating that this medicine might be an advantageous supplement for sperm preparation media.

**Acknowledgements:** UMIB (Pest-OE/SAU/UI0215/2014), MGA (SFRH/BPD/80451/2011) and RLB (SFRH/BD/103105/2014) were funded by National Funds through FCT-Foundation for Science and Technology and AR by the Master Course in Molecular Medicine and Oncology, FMUP.

### CO7 – Maternal age and placenta redox changes

Soares AI, MSc^1^, Silveira S, MSc^1^, Matos L, MSc^1,2^, Guedes-Martins L, MD^1,3^, Saraiva J, MD^3,4^, Almeida H, MD, PhD^1,5^, Silva E, PhD^1^. ^1^Ageing and Stress Group, I3S Instituto de Investigação e Inovação em Saúde; IBMC - Instituto de Biologia Celular e Molecular and Departamento de Biologia Experimental, Faculdade de Medicina, Universidade do Porto, Portugal, ^2^Faculdade de Ciências da Nutrição e Alimentação, Universidade do Porto, Portugal, ^3^Centro Hospitalar do Porto EPE, Departamento da Mulher e da Medicina Reprodutiva, Largo Prof. Abel Salazar, 4099-001 Porto, Portugal, ^4^Obstetrics-Gynecology, Private Hospital Trofa, 4785-409 Trofa, Portugal, ^5^Obstetrics-Gynecology, Hospital-CUF Porto, 4100-180 Porto, Portugal (e-mail: esilva@med.up.pt)

In pregnancy, reactive oxygen species play a physiological role in placenta establishment. Therefore, it is believed that a local imbalance in redox homeostasis underlies abnormal placental development and function resulting in pregnancy complications. As their occurrence is associated with increased maternal age, it was hypothesized that at an older reproductive age, loss of redox homeostasis is a contributor to disruption of foetal/placental interactions and the development of such complications. To address this hypothesis, an evaluation of oxidative stress (OS) markers was performed in samples of placenta and uterine placental site collected from women at different ages. All human samples were collected at delivery by elective caesarean section. The protocol was approved by the ethical committee of “Centro Materno-Infantil do Porto” and volunteers gave written consent to be included in the study. Western blotting was used to detect protein carbonylation and expression of the antioxidant enzymes SODI and SODII. The association between OS markers and maternal age was calculated using the Pearson correlation coefficient. A P value less than 0.05 was assumed to denote significant difference. A strong correlation between protein carbonylation and maternal age was observed. Protein carbonylation correlated positively with maternal age in samples collected from placental site (r = 0.4241; P = 0.1016) and negatively with those collected from placenta (r = 0.4068; P = 0.1052). In placental site, a highly carbonylated protein of 66 kDa showed a strong positive correlation with maternal age (r = 0.5272; P = 0.0434). SODI expression correlated negatively with maternal age in both tissues studied (r = -0.3291; P = 0.2311 and r = -0.4201; P = 0.0932 for placental site and placenta tissue, respectively). SODII expression showed a strong positive correlation with maternal age at the placental site (r = 0.4558; P = 0.0499) but not at the placenta (r = -0.0778; P = 0.7667). Data shows selective redox balance disturbances in foetal/placental interface of aged women. They favour the existence of oxidative stress on the woman's tissue (placental bed), not accompanied by similar effects on the foetal derived tissues. Future studies should focus on elucidating the role of these alterations in the regulation of uterine tissue remodelling and placentation.

Acknowledgments: Prémio Crioestaminal 2011; FCT grants: SFRH/BD/61820/2009 and SFRH/BPD/72536/2010.

### CO8 – Cannabinoid signaling in human placenta: effects of cannabinoids in cytotrophoblast cells

LF Costa, BSc^1,2^, BM Fonseca, PhD^2^, C Amaral, PhD^2^, A Mendes, MD^3^, J Braga, MD^3^, NA Teixeira, PhD^2^, G Correia-da-Silva, PhD^2^. ^1^Departamento de Biologia, Universidade de Aveiro, Portugal, ^2^UCIBIO, REQUIMTE, Laboratório de Bioquímica, Departamento de Ciências Biológicas, Faculdade de Farmácia, Universidade do Porto, Portugal, ^3^Departamento da Mulher e da Criança, Serviço de Obstetrícia, Centro Materno-Infantil do Norte-Centro Hospitalar do Porto, Portugal (e-mail: george@ff.up.pt).

Cannabinoids (CBs) are active compounds of *Cannabis sativa*. The most prevalent psychoactive substance is Δ9-tetrahydrocannabinol (THC), whose effects are achieved through the activation of cannabinoid receptors – CB1 and CB2. Due to its medicinal properties, or as drugs of abuse, some cannabinoids started to be synthetized artificially. On the other hand, there are endogenous lipid compounds, called endocannabinoids (eCBs) that are capable of binding and activate the same cannabinoid receptors, being anandamide (AEA) and 2-arachidonoylglycerol (2-AG) the major eCBs. Placenta development requires a tight regulated proliferation, differentiation and apoptosis of cytotrophoblasts. Recently, we have shown that eCBs are able to induce apoptosis and impair cytotrophoblasts differentiation into syncytiotrophoblasts. The exogenous administration of cannabinoids may interfere with this delicate balance of trophoblast turnover. In this work, we studied the effects of CBs in primary cultures of human cytotrophoblasts and in BeWo cells, a cytotrophoblast cell model. Cells were treated for 24 h with different concentrations of 2-AG, THC and the synthetic cannabinoid WIN-55,212. We used MTT and LDH release assays to access cell viability; morphological changes were analyzed by Höechst and Giemsa staining whereas ROS/RNS species generation and mitochondrial membrane potential (Δψm) were evaluated by fluorimetry assays; glutathione levels were measured by spectrophotometry and cell cycle was analyzed by flow cytometry. Treatment with THC did not affect cell viability. Low concentrations of WIN·55,212 resulted in loss of cell viability, while for 2-AG this effect was only observed for higher concentrations. These findings were supported by morphological analysis. All cannabinoids induced a decrease in Δψm, but only 2-AG led to an increase in ROS/RNS intracellular levels though no changes in glutathione levels were observed. In addition to the decrease in cell viability, WIN·55,212 treatment induced a cell cycle perturbation in G(2)/M- and S-phases. Here we reinforce the importance of cannabinoid signaling in placenta. Deregulation of this signaling network may be implicated in alterations of oxidative state of cytotrophoblasts and proliferation/apoptosis and contribute to the pathophysiological mechanisms of some pregnancy complications.

Acknowledgments: FCT for B. Fonseca and C. Amaral grants (SFRH/BPD/72958/2010; SFRH/BPD/98304/2013).

### P01 – Patients with Y chromosome microdeletions: embryological and clinical outcomes

Carolina Gonçalves, MSc^1,2^, Mariana Cunha, BSc^3^, Eduardo Rocha, PhD^4^, Susana Fernandes, PhD^5^, Joaquina Silva, MD^3^, Paulo Viana, BSc^3^, Luís Ferraz, MD^6^, José Teixeira da Silva, MD^3^, Cristiano Oliveira, MD^3^, Alberto Barros, MD, PhD^3,5^ and Mário Sousa, MD, PhD^2^. ^1^Department of Biology, University of Aveiro (UA), Campus Universitário de Santiago, 3810-193 Aveiro, Portugal, ^2^Department of Microscopy, Laboratory of Cell Biology, Institute of Biomedical Sciences Abel Salazar, University of Porto (ICBAS-UP), Rua Jorge Viterbo Ferreira, 228, 4050-313 Porto, Portugal; Unit for Multidisciplinary Research in Biomedicine-UMIB, ICBAS-UP, ^3^Centre for Reproductive Genetics Prof. Alberto Barros (CGR), Av. do Bessa, 240, 1° Dto. Frente, 4100-009 Porto, Portugal, ^4^Department of Microscopy, Laboratory of Histology and Embryology, ICBAS-UP, Rua Jorge Viterbo Ferreira, 228, 4050-313 Porto, Portugal, ^5^Department of Genetics, Faculty of Medicine, University of Porto (FMUP), Alameda Prof. Hernâni Monteiro, 4200-319 Porto, Portugal; Institute for Innovation and Health Research (I3S), University of Porto, Portugal, ^6^Department of Urology, Hospital Center of Vila Nova de Gaia, Rua Conceição Fernandes, 4430-502 Vila Nova de Gaia, Portugal (e-mail: msousa@icbas.up.pt)

About 15% of couples present infertility and half of these cases are due to male factors. After Klinefelter syndrome, Y-chromosome microdeletions are the most frequent genetic cause of male infertility, frequently associated to azoospermia and severe oligozoospermia. The locus defined as Azoospermia Factor (AZF) contain the genes necessary for normal spermatogenesis and detection of microdeletions in this locus have been proposed as a tool for infertility diagnosis. We aim to present the outcomes of patients with Y-chromosome microdeletions treated by intracytoplasmic sperm injection (ICSI), either using fresh (TESE) or frozen-thawed (TESE-C) testicular sperm, and ejaculated sperm (EJAC). The originality of this work resides in the comparisons between the different types of Y-microdeletions (AZFa, AZFb, AZFc) and treatments (TESE, TESE-C, EJAC), with detailed demographic, stimulation, embryological, clinical and newborn (NB) outcomes. Methods: We evaluated 128 patients with AZF microdeletions to determine if there are any significant differences in outcomes between the different types of Y-microdeletions using TESE, TESE-C or EJAC. Results: Of 128 patients with Y-microdeletions, 18 performed ICSI with ejaculated sperm and 65 went for TESE. There were 51 TESE treatment cycles and 43 TESE-C treatment cycles, with birth of 19 NB (2 in AZFa/TESE-C; 12 in AZFc/TESE; 5 in AZFc/TESE-C). Of the 29 EJAC cycles there was birth of 8 NB (in AZFc). In TESE and EJAC cycles there were no significant differences in embryological and clinical parameters. In TESE-C cycles, there was a significant lower oocyte maturity rate, embryo cleavage rate, mean number of day-3 embryos and mean number of embryos transferred in AZFb, and a higher mean number of oocytes and lower fertilization rate in AZFc. There was 1 major malformation (in AZFc/TESE), 1 NB with very preterm delivery (in AZFc/TESE), 2 NB with preterm birth delivery (in AZFc: 1 in TESE-C; 1 in EJAC) and 8 NB with low birth weight (in AZFc: 3 in TESE, 2 in TESE-C and 3 in EJAC). Conclusions: Although patients with AZFc microdeletions presented a high testicular sperm recovery rate and acceptable clinical outcomes, cases with AZFa and AZFb microdeletions presented a poor prognosis. Due to the reported heredity of microdeletions, patients should be informed about the infertile consequences on the NB and the possibility of using preimplantation genetic diagnosis for female sex selection.

Aknowledgements: For oocyte retrieval to Jorge Beires, MD, PhD, Gynecologist, Dept. of Gynecology and Obstetrics, Director of the Unit of Gynecology and Reproductive Medicine, Hospital of S. John, E.P.E, Porto, Portugal and José Manuel Teixeira da Silva, MD, Ginecologist. For anesthesiology to José Correia, MD, Anesthetist (Department of Anesthesiology, Hospital of S. John, E.P.E, Porto, Portugal). For spermiology laboratorial work to Cláudia Osório, BSc, Ana Gonçalves, BSc, and Nuno Barros BSc (CGR-ABarros). For cytogenetics support to Sofia Dória, PhD, Carolina Almeida, PhD, Ana Grangeia, PhD and Maria João Pinho, PhD (Department of Genetics, FMUP). This work was funded by the author's Institutions and in part by Unit for Multidisciplinary Research in Biomedicine -UMIB, ICBAS-UP (National Funds through FCT-Foundation for Science and Technology, under the Pest-OE/SAU/UI0215/2014).

### P02 – Estrogen receptors alpha (ER-a), beta (ER-b) and G protein- coupled receptor 30 (GPR30) in the testicular tissue of individuals with Klinefelter syndrome

Raquel L. Bernardino, MSc^1,2^, Marco G. Alves, PhD^3^, Joaquina Silva, MD^4^, Alberto Barros, MD, PhD^4,5^, Luis Ferraz, MD^6^, Mário Sousa, MD, PhD^1,2,4^, Rosália Sá, PhD^1,2^, Pedro F. Oliveira, PhD^1,2^. ^1^Department of Microscopy, Laboratory of Cell Biology, Institute of Biomedical Sciences Abel Salazar (ICBAS), University of Porto, Porto, Portugal, ^2^Unit for Multidisciplinary Research in Biomedicine (UMIB), Institute of Biomedical Sciences Abel Salazar (ICBAS), University of Porto, Porto, Portugal, ^3^CICS – UBI – Health Sciences Research Centre, University of Beira Interior, Covilhã, Portugal, ^4^Centre for Reproductive Genetics Prof. Alberto Barros, Porto, Portugal, ^5^Department of Genetics, Faculty of Medicine and Institute of Health Research and Innovation, University of Porto, Porto, Portugal, ^6^Department of Urology, Hospital Center of Vila Nova de Gaia, Vila Nova de Gaia, Portugal. (e-mail: pfobox@gmail.com)

Klinefelter syndrome (KS) is characterized by X chromosome polysomy, with X disomy being the most recurrent variant. KS is frequently associated with hypogonadism, infertility and gynaecomastia. Adults with KS are characterized by severe hormonal deregulation, particularly hypergonadotropic hypogonadism with elevated serum concentrations of estradiol (E2). Estrogens play important roles in the regulation of testes development and spermatogenesis, through the interaction with their specific receptors. The expression of E2 receptors, namely G protein-coupled estrogen receptor (GPR30), estrogen receptor α (ERα) and estrogen receptor β (ERβ), has been described in male reproductive tract, but no data is available on the expression of these receptors in men with KS. Herein, we aimed to evaluate the mRNA expression of GPR30, ERα and ERβ in testis of men with KS as compared to those with conserved spermatogenesis and 46XY karyotype. Six human testicular biopsies were obtained from individuals with an average age of 44±4 years, confirmed 46XY karyotype, conserved spermatogenesis and seeking fertility treatment due to anejaculation, vasectomy or traumatic section of vas deferens (Control group). Other six testicular biopsies were obtained from azoospermic men with KS, average age of 34±3 years and confirmed homogeneous 47XXY karyotype (KS group). RNA was extracted from testicular tissue. cDNA was synthesized for each sample and gene expression of the three estrogen receptors (GPR30, ERα and ERβ) was evaluated by reverse transcriptase polymerase chain reaction (RT-PCR) and quantitative PCR (RT-qPCR). We were able to detect the presence of ERα, ERβ and GPR30 transcripts in the testicular tissue of individuals from Control and KS groups by RT-PCR. Our results show that ERβ transcripts are the most abundant in the testicular tissue of 46XY men. Furthermore, the testicular abundance of GPR30 mRNA in men with KS was approximately twelve times higher as compared to that of men with conserved spermatogenesis. The results presented herein suggest that GPR30 is an important mediator of E2 effects over steroidogenesis and may be partly responsible for the testicular alterations observed in men with KS. Hence, although the role of GPR30 in testes of KS men needs to be fully investigated, this E2 receptor may be a possible therapeutic target in order to reduce the implications of the higher levels of E2 observed in those individuals.

**Acknowledgements:** This work was supported by FCT (PTDC/QUI-BIQ/ 121446/2010, SFRH/BPD/80451/2011 and SFRH/BD/103105/2014) through FSE and POPH funds (Pest-OE/SAU/UI0215/2014).

### P03 – Testicular metabolic reprogramming in neonatal streptozotocin-induced type 2 diabetic rats impairs glucose metabolism and promotes glycogen accumulation

L Rato, PhD^1^, MG Alves, PhD^1,2^, TR Dias, MSc^1^, JE Cavaco, PhD^1^, PF Oliveira, PhD^1,3,4^. ^1^CICS – UBI – Health Sciences Research Centre, Faculty of Health Sciences, University of Beira Interior, 6201-506 Covilhã, Portugal, ^2^Department of Life Sciences, Faculty of Sciences and Technology and Center for Neurosciences and Cell Biology (CNC), University of Coimbra, Portugal, ^3^Department of Microscopy, Laboratory of Cell Biology, Institute of Biomedical Sciences Abel Salazar (ICBAS), University of Porto, Portugal, ^4^Unit for Multidisciplinary Research in Biomedicine, Abel Salazar Institute of Biomedical Sciences, University of Porto – UMIB/ICBAS/UP (e-mail: pfobox@gmail.com)

Type 2 diabetes mellitus (T2DM) is one of the most prevalent and serious metabolic diseases affecting young males on modern societies. This pathological condition is closely related to current lifestyle habits and induces metabolic alterations particularly in glucose metabolism, which is vital for the normal occurrence of spermatogenesis. Defects in testicular glucose metabolism have been associated with decline of male fertility, but most of the mechanisms associated with T2DM-induced male infertility remain unknown. We aimed to evaluate the effects of T2DM on testicular glucose metabolism by using a neonatal-streptozotocin (n-STZ)-induced T2DM animal model. Plasma and testicular hormonal levels were evaluated by using specific kits. mRNA and protein expression levels were assessed by real-time PCR and Western blot, respectively. Testicular metabolic profile was assessed by ^1^H-NMR spectroscopy. T2DM rats showed increased glycemia, glucose intolerance and hyperinsulinemia. Both testicular and serum testosterone levels were decreased, whereas those of 17β-estradiol were not altered. Testicular glycolytic flux was not favored in testes of T2DM rats, since despite the increased expression of both glucose transporters 1 and 3 and the enzyme phosphofructokinase 1, lactate dehydrogenase activity was severely decreased contributing to lower testicular lactate content. However, T2DM enhanced testicular glycogen accumulation, by modulating the availability of the precursors for its synthesis. T2DM also affected the reproductive sperm parameters. Altogether these results indicate that T2DM is able to reprogram testicular metabolism by enhancing alternative metabolic pathways, in particular glycogen synthesis and such alterations are associated with impaired sperm parameters.

**Acknowledgements:** To Maria João Silva and Maria José Pinto for their technical assistance in animal care and handling; Johnsons Portugal and Dalila Côrte for providing the glucometer and compatible reactive strips. Portuguese “Fundação para a Ciência e a Tecnologia”—FCT cofunded by FEDER via Programa Operacional Factores de Competitividade— COMPETE/QREN [PEst-C/SAU/UI0709/2011]. LR [SFRH/BD/72733/2010], MG Alves [SFRH/BPD/80451/2011] and PFO (PTDC/QUI-BIQ/121446/2010) were financed by FCT through FSE and POPH Funds.

### P04 – How does Sertoli cell Metabolism Responds to the Reduced Testosterone Levels Induced by Progressive Stages of Diabetes Mellitus?

JE Cavaco^∗^, PhD^1^, L Rato^∗^, PhD^1^, MG Alves, PhD^1,2^, AI Duarte, PhD^2,3^, MS Santos, PhD^2,4^, PI Moreira, PhD^2,5^, PF Oliveira, PhD^1,6^. ^1^CICS – UBI – Health Sciences Research Centre, Faculty of Health Sciences, University of Beira Interior, 6201-506 Covilhã, Portugal, ^2^CNC – Centre for Neuroscience and Cell Biology, University of Coimbra, Portugal, ^3^Institute for Interdisciplinary Research (IIIUC), University of Coimbra, Portugal, ^4^Life Sciences Department, Faculty of Sciences and Technology, University of Coimbra, Portugal, ^5^Laboratory of Physiology, Faculty of Medicine, University of Coimbra, Portugal, ^6^Department of Microscopy, Laboratory of Cell Biology, Institute of Biomedical Sciences Abel Salazar (ICBAS) and Unit for Multidisciplinary Research in Biomedicine (UMIB), University of Porto, Portugal (e-mail: pfobox@gmail.com) ^∗^both authors contributed equally

Diabetes mellitus (DM) is a metabolic disease that compromises male fertility through the induction of hormonal deregulation. Within the seminiferous epithelium, androgen receptors are exclusively expressed in Sertoli cells (SCs). Under culture conditions, androgens induce a metabolic shift from a Warburg-like to an oxidative Krebs cycle metabolism in SCs. Under detrimental conditions, SCs use alternative substrates as a compensatory mechanism to ensure the adequate conditions for germ cell development and to counteract the deleterious effects of DM. At the present work we aimed to study how SCs metabolism responds to reduced testosterone (T) levels induced by DM. SCs obtained from normal Wistar strain rats (n = 6) and from rodent models of prediabetes (PreD) (n = 6) and type 2 diabetic mellitus (T2DM) (n = 6) were cultured during 96 hours with sex steroid concentrations within the physiologic range (T-CTR group), PreD conditions (T-PreD group) and T2DM conditions (T-T2DM group). Metabolite secretion/consumption profile of cultured SCs was evaluated by ^1^H-NMR spectroscopy. Protein expression levels were assessed by Western blot. Intracellular glycogen content was quantified by using specific kits. Lactate dehydrogenase activity was determined using a commercial assay kit. Alanine aminotransferase activity was determined by spectrophotometric methods. Both glucose and pyruvate consumption were significantly decreased in PreD conditions, whereas T2DM conditions reversed this profile. Lactate production was not significantly altered at the end of the treatment, although the expression and activities of the lactate production-associated proteins were increasingly affected by progressive T-deficiency conditions. Alanine production was significantly increased in SCs of both groups, suggesting an alternative metabolic fuel. Notably, intracellular glycogen content was only increased in SCs of the T2DM group. These results illustrate that gradually reduced T levels, induced by progressive stages of DM, impair glycolysis favoring glycogen metabolism, with the more pronounced effects being concurrent with lower T levels. Even in the T2DM conditions, SCs were able to adapt their metabolism to sustain lactate metabolism. This report highlights the physiologic significance of T in the regulation of the glycolytic profile of SCs metabolism, in particular when associated with the progression of T2DM.

**Acknowledgements:** This work was supported by the Portuguese “Fundação para a Ciência e a Tecnologia” (FCT), co-funded by FEDER via Programa Operacional Factores de Competitividade-COMPETE/QREN [PTDC/ QUI-BIQ/121446/2010 and PEst-C/SAU/UI0709/2014]. L Rato [SFRH/BD/72733/2010], MG Alves [SFRH/BPD/80451/2011] and AI Duarte [SFRH/BPD/84473/2012] were financed by FCT. P.F. Oliveira was financed by FCT through FSE-POPH funds (Programa Ciência 2008). The authors are thankful to Milaydis Sosa Napolskij, BA, for professional English proofreading and linguistic suggestions.

### P05 – Sperm quality of pre-diabetic wistar rats is restored after a diet with white tea

Branca M Silva, PhD^1^, Gonçalo D Tomás, MSc^1^, Tânia R Dias, MSc^1^, Ana D Martins, MSc^1,2^, Luís Rato, PhD^1^, Marco G Alves, PhD^1^, Pedro F Oliveira, PhD^2^. ^1^CICS – UBI – Health Sciences Research Centre, University of Beira Interior, 6201-506 Covilhã, Portugal, ^2^Department of Microscopy, Laboratory of Cell Biology, Institute of Biomedical Sciences Abel Salazar (ICBAS) and Unit for Multidisciplinary Investigation in Biomedicine (UMIB - UID/Multi/00215/2013), University of Porto, Portugal (e-mail: bmcms@ubi.pt)

Pre-diabetes is a major risk factor for the development of type 2 diabetes mellitus. This prodromal stage encompasses some, but not all, of type 2 diabetes mellitus diagnostic criteria. Pre-diabetes has been recently associated with altered testicular function and increased testicular oxidative stress. Tea is widely consumed and its anti-hyperglycemic/antioxidant properties are well known. We aimed to evaluate if white tea consumption by pre-diabetic rats could prevent testicular OS, preserving sperm quality. The phytochemical profile of white tea infusion was evaluated by proton nuclear magnetic resonance (1H-NMR). White tea was given to 30-day-old streptozotocin-induced pre-diabetic rats during two months. Testicular antioxidant potential and oxidative stress markers, as well as sperm parameters, were evaluated using routine techniques. The white tea infusion presented a high content of polyphenols. White tea consumption improved glucose tolerance and insulin sensitivity in prediabetic rats. Testicular antioxidant potential was increased by white tea consumption, restoring protein oxidation and lipid peroxidation, although glutathione content and redox state were not altered. White tea consumption improved sperm concentration and sperm quality (motility, viability and abnormality) was restored. Overall, white tea consumption improved sperm quality of pre-diabetic rats, decreasing testicular oxidative damage. Based on our results, white tea consumption appears as a natural, economical and effective strategy to counteract the deleterious effects of pre-diabetes on male reproductive health but further studies will be needed before a definitive recommendation.

### P06 – Testicular metabolic biomarkers in men with Klinefelter syndrome

Susana P Almeida, MSc^1^, Ana D Martins, MSc^1,2^, Ivana Jarak, PhD^3^, Alberto Barros, MD, PhD^4,5,6^, Joaquina Silva, MD^4^, Mário Sousa, MD, PhD^1,4^, Pedro F Oliveira, PhD^1,2,6^, Marco G Alves, PhD^2^. ^1^Department of Microscopy, Laboratory of Cell Biology, Abel Salazar Institute of Biomedical Sciences (ICBAS) and Unit for Multidisciplinary Research in Biomedicine (UMIB), ^2^CICS – UBI – Health Sciences Research Centre, University of Beira Interior, 6201-506 Covilhã, Portugal; University of Porto, 4050-313 Porto, Portugal, ^3^CICECO, Univesity of Aveiro, 3810-193 Aveiro, Portugal, ^4^Centre for Reproductive Genetics Professor Alberto Barros, 4100-009 Porto, Portugal, ^5^Department of Genetics, Faculty of Medicine, University of Porto, 4200 - 319 Porto, Portugal ^6^Institute for Innovation and Research in Health (I3S), University of Porto, Porto, Portugal (e-mail: alvesmarc@gmail.com)

Klinefelter syndrome (KS) is the most common genetic cause of human infertility but the exact mechanisms by which it occurs remain largely unknown. KS men are reported to have alterations in body composition and high risk of developing metabolic diseases. Thus, we hypothesized that KS men present a distinct testicular metabolic profile that may alter the nutritional support of spermatogenesis. Testicular biopsies from control (46, XY) (n = 6) and KS (47, XXY) (n = 6) were collected and analyzed by proton high-resolution magic angle spinning nuclear magnetic resonance spectroscopy. The mRNA and protein expressions of crucial glycolysis-associated enzymes and transporters were determined by qPCR and Western blot, respectively. Several genes presented an upstream regulation in the testis of KS men, including those of glucose transporters, phosphofructokinase-1 and lactate dehydrogenase A expression. Our results show that testicular tissue of men with KS has a severe decrease in lactate and creatine concentration. Overall, the testicular tissue from men with KS seeking for fertility treatment present a specific testicular metabolic phenotype, when compared with 46, XY control men, with dramatic consequences to the nutritional support of spermatogenesis. We detected important biomarkers in the testis of KS men that may be associated with the infertility condition that these men face or with the onset of the condition.

**Acknowledgements:** This work was supported by the Portuguese “Fundação para a Ciência e a Tecnologia”—FCT: SPA (SFRH/BD/112012/2015), MGA (SFRH/BPD/80451/2011 and PTDC/BIM-MET/4712/2014); PFO (SFRH/BPD/108837/2015 and PTDC/BBB-BQB/1368/2014); CICS-UBI (Pest-C/SAU/UI0709/2014); UMIB (Pest-OE/SAU/UI0215/2014) co-funded by FEDER via Programa Operacional Fatores de Competitividade-COMPETE/QREN & FSE and POPH funds.

### P07 – Mutation analysis in patients with total sperm immotility and dysplasia of the fibrous sheath

Rute Pereira, MSc^1,2^, Jorge Oliveira, MSc ^3,4^, Luis Ferraz, MD^5^, Alberto Barros, MD, PhD^6,7,8^, Rosário Santos, PhD^3,4^, Mário Sousa, MD, PhD^1,3^. ^1^Laboratory of Cell Biology, Department of Microscopy, Institute of Biomedical Sciences Abel Salazar (ICBAS), University of Porto (UP), Rua Jorge Viterbo Ferreira, 228, 4050-313Porto, Portugal, ^2^Department of Biology, Faculty of Sciences, University of Porto, Rua do Campo Alegre 1021/1055, 4169-007 Porto, Portugal Portugal, ^3^Multidisciplinary Unit for Biomedical Research (UMIB - UID/Multi/00215/2013), ICBAS-UP, ^4^Molecular Genetics Unit, Centre of Medical Genetics Dr. Jacinto Magalhães (CGMJM), Hospital Centre of Porto (CHP), Praça Pedro Nunes, 88, 4099-028 Porto, Portugal, ^5^Department of Urology, Hospital Eduardo Santos Silva, Hospital Centre of Vila Nova de Gaia (CHVNG), Espinho, E.P.E., Rua Conceição Fernandes, 4434-502 Vila Nova de Gaia, Portugal, ^6^Centre of Reproductive Genetics Alberto Barros (CGR), Av. do Bessa, 240, 1° Dto. Frente, 4100-012 Porto, Portugal, ^7^Department of Genetics, Faculty of Medicine, University of Porto, Alameda Prof. Hernâni Monteiro, 4200-319 Porto, Portugal, ^8^Institute for Innovation and Health Research (I3S), UP, Porto, Portugal (e-mail: msousa@icbas.up.pt)

The axoneme is the flagellar motor of sperm. It contains nine peripheral microtubule doublets and a central pair of microtubules. Doublets are linked by nexin bridges; present dynein arms (DA) and are linked to the central microtubule pair by radial spokes (RS). The central pair, surrounding fibrilar sheath and central bridge is named the central pair complex (CPC). The axoneme is surrounded by outer dense fibres and the fibrous sheath (FS). Total sperm immotility (1:5000 men) causes male infertility and is due to genetic changes visible on ultrastructural defects in the sperm flagellum. We analysed four men with total sperm immotility associated with DFS and disruption of several axoneme structures. Using Sanger sequencing, we screened 7 genes (CCDC39 and CCDC40- DA, nexin links, RS, CPC, doublets; DNAH5 and DNAI1-DA; RSPH1-RS, CPC; AKAP3 and AKAP4-FS) involved in sperm motility and whose mutations are associated to ultrastructural axoneme defects. Sperm of Patient-1 presented Dysplasia of the Fibrous Sheath (DFS), with loss of CPC and RS. We found 5 rare variants and 1 novel variant. Rare variants in DNAH5: 2 non-pathogenic and 1 possible pathogenic. Rare variant in DNAI1: 1 possible pathogenic. Novel variant in CCDC39: 1 possible pathogenic. Sperm from Patient-2 presented DFS, with a variable number of doublets and RS. We found 3 rare variants. Rare variants in DNAH5: 1 polymorphism, 1 possible pathogenic. Variant in CCDC39: 1 possible pathogenic. Sperm of Patient-3 presented DFS, with absence of DA, nexin bridges and CPC. We found 5 rare variants and 3 novel variants. Rare variant in CCDC39: 1 non-pathogenic. Variants in CCDC40: 1 possible pathogenic, 1 new variant non-pathogenic. Variants in DNAH5: 2 variants non-pathogenic, 2 new variants non-pathogenic, 1 variant possible pathogenic. Sperm of Patient-4 presented DFS, with absence of CPC and RS. We found 4 rare variants. Variants in DNAH5: 2 non-pathogenic, 1 possible pathogenic. Variant in CCDC40: 1 non-pathogenic. No AKAP mutations were found. Variants found in these patients will be further analysed (expression analysis/functional studies) to confirm their pathogenicity and association with DFS and total sperm immotility. We also expect to perform WES analysis in order to expand findings to the whole genome.

### P08 – Leptin modulates human Sertoli cells glucose metabolism: relevance for obesity-induced male infertility

Ana D Martins, MSc^1,2,3^, Ana C Moreira, PhD^4^, Rosália Sá, PhD^2,3^, Mariana P Monteiro, MD, PhD^3,5^, Mário Sousa MD, PhD^2,3,6^, Rui A Carvalho, PhD^7^, Branca M Silva, PhD^1^, Pedro F Oliveira, PhD^2,3^ and Marco G Alves, PhD^1,7^. ^1^CICS-UBI - Health Sciences Research Centre, University of Beira Interior, Covilhã, Portugal, ^2^Department of Microscopy, Laboratory of Cell Biology, Abel Salazar Institute of Biomedical Sciences (ICBAS), University of Porto, Porto, Portugal, ^3^Unit for Multidisciplinary Research in Biomedicine, Abel Salazar Institute of Biomedical Sciences (UMIB-ICBAS), University of Porto, Porto, Portugal, ^4^Institute for Molecular and Cell Biology (IBMC) and Institute of Health Research and Innovation (I3S), University of Porto, Porto, Portugal, ^5^Department of Anatomy, Abel Salazar Institute of Biomedical Sciences (ICBAS), University of Porto, Porto, Portugal, ^6^Centre for Reproductive Genetics Professor Alberto Barros, Porto, Portugal, ^7^Department of Life Sciences, Faculty of Sciences and Technology and Center for Neurosciences and Cell Biology (CNC), University of Coimbra, Coimbra, Portugal (e- mail: alvesmarc@gmail.com)

Metabolic diseases, including obesity and type 2 diabetes may be a consequence of the current lifestyle dominated by a poor nutrient diet and a marked physical inactivity. The hormone leptin, known as the “satiety hormone”, is produced in adipose tissue and is positively correlated with fat mass. It has been suggested that the hypothalamus may be the primary target for most of leptin actions on the reproductive axis. However, the effects of this hormone on particular molecular mechanisms that control male fertility remain unexplored. Herein, we hypothesize that leptin affects spermatogenesis by altering Sertoli cells (SCs) metabolism. Primary cultures of human SCs (hSCs) were treated, during 24 h, at 33°C, 5%CO_2_, with different concentrations of leptin (5 ng/mL as reported in lean patients, 25 ng/mL as reported in obese patients and 50 ng/mL as reported in morbidly obese patients). The results were compared to a control condition (without leptin). Leptin receptor was identified by qPCR and Western blot. Western Blot was performed to determine protein levels of glucose transporters (GLUTs), phosphofructokinase, lactate dehydrogenase (LDH) and monocarboxylate transporter 4. Lipid peroxidation, protein carbonilation and nitration were determined to evaluate oxidative damages. Metabolite concentration in extracellular media was determined by ^1^H-NMR. LDH activity was evaluated by a commercial kit assay. Our results demonstrate that hSCs express the leptin receptor. GLUT2 protein levels were upregulated in hSCs treated with leptin levels found in lean patients and LDH activity was increased in hSCs treated with concentrations of leptin found in lean and obese patients. Interestingly, leptin severely decreased acetate production by hSCs. Finally, no oxidative damages were found in hSCs treated with leptin. The decreased acetate production by hSCs exposed to leptin suggests that this hormone may be essential in the control of metabolic intermediates needed for lipids synthesis and membrane remodeling in developing germ cells. This is a first report showing that leptin can affect the metabolic support of spermatogenesis by hSCs. Further studies will be needed to disclose the role of leptin in Sertoli-germ cells interaction and its relevance for male fertility.

Acknowledgments: This work was supported by the Portuguese “Fundação para a Ciência e a Tecnologia” - FCT: MG Alves (SFRH/ BPD/80451/2011; PTDC/BIM-MET/4712/2014); PF Oliveira (PTDC/BBB-BQB/1368/2014); CICS-UBI (Pest-C/SAU/UI0709/2014) and UMIB (Pest-OE/SAU/UI0215/2014) co-funded by Fundo Europeu de Desenvolvimento Regional - FEDER via Programa Operacional Factores de Competitividade - COMPETE/ QREN, FSE and POPH funds.

### P09 – Towards human endometrium decidualization: a dialogue between endocannabinoids and prostaglandins

Marta Almada, MD^1^, Fabiana Piscitelli, PhD^2^, Georgina Correia- da-Silva, PhD^1^, Vincenzo Di Marzo, PhD^2^, Natércia A. Teixeira, PhD^1^, and Bruno M. Fonseca, PhD^1^. ^1^UCIBIO, REQUIMTE, Laboratory of Biochemistry, Biological Sciences Department, Faculty of Pharmacy, University of Porto, Portugal, ^2^Endocannabinoid Research Group, Institute of Biomolecular Chemistry, Consiglio Nazionale delle Ricerche, Pozzuoli (NA), Italy (e-mail: brunofonseca@ff.up.pt)

The human endometrium is a dynamic tissue that undergoes growth, differentiation, and regression periods throughout the menstrual cycle. The process in which endometrial stromal cells proliferate and differentiate into specialized decidual cells, named decidualization, represents a tipping point to prime the uterus for implantation (1). A network of signaling molecules, hormones and cytokines is coordinated towards the development of a receptive endometrium. Amongst those, lipid molecules such as the prostaglandins (PGs) and endocannabinoids (eCBs) are frontline mediators (1). Impaired achievement of endometrial receptivity is increasingly linked to common reproductive disorders and infertility (2). Over the last years, an attempt to define and characterize endometrial receptivity, has put forward the search for new molecules, differentially expressed throughout the window of implantation. Not only it will allow the identification of putative markers, but utmost guide the clinical acquisition of a receptive endometrium. In this study, we aimed to decipher the dialogue between eCBs and PGs upon the receptive endometrium. For that, we employed two culture systems, an endometrial stromal cell line (St-T1b), and human decidual fibroblasts (HdF), isolated from human term placenta, in which undifferentiated stromal cells undergo decidualization upon stimulation. Firstly, we investigated the levels of anandamide (AEA), the main eCB, in both non-differentiated and differentiated cell types, by a LC-MS-MS method. Furthermore, we found that AEA inhibited human decidualization, by decreasing the levels of the decidual markers, IGFBP1 and PRL, by Q-PCR. These effects were partially reversed by pretreatment with the cannabinoid receptor (CB1) antagonist (AM251 and AM281). As PGs are crucial factors for decidualization, we investigated the effects of AEA upon PGS levels in human decidualization. Therefore, we have developed a UPLC-MS-MS protocol to detect PGs in human endometrial stromal cells. We have found that AEA prevented the production of PGE2 during the decidualization process, through a CB1 dependent mechanism. The importance of understanding the mechanisms that influence the production of prostaglandins in the endometrium is clinically relevant because it may shed light on the sequence of events that leads to successful embryo implantation. In sum, our results suggest that AEA may inhibit human decidual process through CB1 and modulation of PG levels. Moreover, we provide novel insights towards the crosstalk between the endocannabinoid and prostaglandins systems in decidual environment. These findings suggest that a deregulation of the intricate network that drives decidualization may implicate pregnancy and fertility. The detection of eCB and PGs levels may, in the future be translated into clinic benefits, to monitor acquisition of receptive endometrium, but also in the diagnosis of potential pregnancy disorders or infertility.

**References:** (1) Fonseca, B.M., et al., Endocrinology, 2010, 151(8): p. 3965-74

(2) Gellersen B., Brosens JJ., Endocrinology, 2014 35(6):851-905

**Acknowledgements:** This work was supported by the Portuguese “Fundação para a Ciência e a Tecnologia”—FCT: Almada M (SFRH/BD/81561/2011) and Fonseca BM (SFRH/BPD/72958/2010).

### P10 – Ultrastructural analysis of a patient with total sperm immotility due to absence of dynein arms and nexin bridges

Elsa Oliveira, BSc^1,2^, Ângela Alves, BSc^1,2^, Helena Figueiredo, MSc^3^, Luís Ferraz, MD^4^, Mário Sousa, MD, PhD^1,2,5^. ^1^Department of Microscopy, Laboratory of Cell Biology, Institute of Biomedical Sciences Abel Salazar (ICBAS), University of Porto (UP), Rua Jorge Viterbo Ferreira, 228, 4050-313, Porto, Portugal, ^2^Unit for Multidisciplinary Research in Biomedicine (UMIB), ICBAS-UP, ^3^Unit for Reproductive Medicine, Hospital Centre of Vila Nova de Gaia (CHVNG)/ Espinho, Rua Francisco Sá Carneiro, 4400-129, Vila Nova de Gaia, Portugal, ^4^Department of Urology, Hospital Eduardo Santos Silva, CHVNG/ Espinho, Rua Conceição Fernandes, 4434-502 Vila Nova de Gaia, Portugal, ^5^Centre for Reproductive Genetics, Alberto Barros, Porto, Portugal (e-mail: msousa@icbas.up.pt)

Asthenozoospermia, defined as reduced or absent sperm motility, is one of the leading causes of male infertility. The spermatozoon is divided in head and flagellum, which is composed of an axoneme and surrounding structures. The axoneme consists of nine peripheral doublets arranged around two central microtubules. Doublets are linked by nexin bridges, have dynein arms and are linked to the central pair by radial spokes. Dynein arms are responsible for motion. Total sperm immotility is associated with specific structural abnormalities, which can only be diagnosed by transmission electron microscopy (TEM). The aim of the present study was to analyze by TEM a semen sample from a patient with total sperm immotility in order to obtain a diagnosis for his infertility. Semen was washed with Sperm Preparation Medium, mixed with sodium cacodylate buffer, 0.1 M, pH 7.2 and centrifuged (10 min, 1500 rpm). The pellet was then fixed with karnovsky (2 h), washed, post-fixed with 2% osmium tetroxide in buffer (2 h, 4°C), dehydrated in a graded ethanol series followed by propylene oxide and embedded in Epon. Semithin sections were stained with aqueous azur II:methylene blue. Ultrathin sections were prepared with a diamond knife on a LKB ultramicrotome, collected on 300 mesh copper grids, double-stained with aqueous uranyl acetate and lead citrate, and observed in a JEOL 100CXII transmission electron microscope operated at 60 kV. Ultrastructural analysis revealed an abnormal head in all sperm. In the majority of the cases, the flagellum structures appeared conserved: basal plate, proximal centriole, striated columns, outer dense fibers, mitochondria sheath, annulus and fibrous sheath. The axoneme showed absence of dynein arms and nexin bridges. Electron microscopy plays a fundamental role in the diagnosis of cases with total sperm immotility. Besides giving a diagnosis, it also provides an indication of the kind of treatment the patient should follow, allows prognosis and advice regarding genetic transmission of the sperm defect to male offspring. The main abnormality detected was the absence of the dynein arms and nexin bridges, which explain sperm total immotility and the consequent infertility of the patient. To get further insight into the possible genetic causes, this patient must be screened for mutations. In this case, the diagnosis allowed us to indicate the use of intracytoplasmic sperm injection for infertility treatment.

## ENDOCRINOLOGY AND DIABETES

### CO9 – White tea consumption improves the glycolytic and oxidative profiles of cerebral cortex of prediabetic Wistar rats: a possible role for neuroprotection?

Ana R Nunes, MSc^1^, Marco G Alves, PhD^1^, Tânia R Dias, MSc^1^, Ana C Cristóvão, PhD^1^, Paula I Moreira, PhD^2^, Pedro F Oliveira, PhD^1,3^, Branca M Silva^1^, PhD. ^1^CICS-UBI – Health Sciences Research Centre, University of Beira Interior, Covilhã, Portugal, ^2^CNC – Center for Neuroscience and Cell Biology, University of Coimbra and Laboratory of Physiology, University of Coimbra, Coimbra, Portugal, ^3^UMIB-ICBAS-UP, Department of Microscopy, Laboratory of Cell Biology and Unit for Multidisciplinary Research in Biomedicine, Abel Salazar Institute of Biomedical Sciences, University of Porto, Porto, Portugal (e-mail: bmcms@ubi.pt)

Diabetes mellitus (DM) is one of the greatest threats to human health and the number of diabetic individuals is rapidly increasing. Prediabetes is a reversible prodromal stage of type 2 DM (T2DM) which encompasses some, but not all, T2DM characteristics. The brain, particularly the cortex, is very susceptible to hyperglycaemia and oxidative stress (OS). Tea (*Camellia sinensis* L.) is one of the most widely consumed beverages. Nevertheless, the biological properties of white tea (WTea) remain unexplored. We hypothesized that WTea daily consumption by prediabetic rats improved the cerebral cortex glycolytic and oxidative profiles. Prenatal male Wistar rats were divided in control group and prediabetes (PrDM) group that received a streptozotocin (STZ) injection. After one month, prediabetic rats were divided in two groups: one consumed water and the other WTea during two months. Rats were then subjected to glucose tolerance and insulin resistance tests. Cerebral cortex glycolytic and oxidative profiles were evaluated. STZ-treated neonatal rats developed mild hyperglycaemia, glucose intolerance and insulin resistance. Prediabetic state decreased lactate content and increased lactate dehydrogenase activity. Moreover, prediabetes decreased cerebral cortex antioxidant capacities, increasing lipid peroxidation and protein oxidation. Daily consumption of white tea improved glucose tolerance and insulin sensitivity in prediabetic rats. It also decreased lactate and alanine contents, and normalized the antioxidant capacity, lipid peroxidation and protein oxidation in cerebral cortex. WTea consumption ameliorated overall metabolic status of prediabetic rats and prevented DM-related effects in cerebral cortex. Thus, WTea consumption appears as an inexpensive and safe co-adjuvant in prediabetes treatment and/or prevention of prediabetes/DM-related dysfunction in cerebral cortex.

Acknowledgments: This work was supported by the “Fundação para a Ciência e a Tecnologia”–FCT co-funded by Fundo Europeu de Desenvolvimento Regional - FEDER via Programa Operacional Factores de Competitividade - COMPETE/QREN to CICS-UBI (Pest-C/SAU/UI0709/2014); TR Dias (SFRH/BD/109284/2015) PF Oliveira (PTDC/BBB-BQB/1368/2014 and SFRH/BPD/108837/2015) and MG Alves (SFRH/BPD/80451/2011 and PTDC/BIM-MET/4712/2014).

### CO10 – Akt/Smad3 signaling increases with adiposity and correlates to transforming growth factor b (TGF-b) levels in human adipose tissue

Adriana R Rodrigues, PhD^1^, Maria J Salazar^1^, Ângela Moreira^3^, Marta Guimarães, MD^4^, Mário Nora, MD^4^, Henrique Almeida, MD, PhD^1^, Mariana P Monteiro, MD, PhD^3^, Alexandra M Gouveia, PhD^1,2^. ^1^Departamento de Biologia Experimental, Faculdade de Medicina; Instituto de Investigação e Inovação em Saúde, Instituto de Biologia Molecular e Celular (IBMC), Universidade do Porto, Portugal, ^2^Faculdade de Ciências da Nutrição e Alimentação, Universidade do Porto, Portugal, ^3^Department of Anatomy, Unit for Multidisciplinary Research in Biomedicine (UMIB), Institute for Biomedical Sciences Abel Salazar (ICBAS), University of Porto, Portugal, ^4^Department of General Surgery, Centro Hospitalar de Entre o Douro e Vouga, Santa Maria da Feira, Portugal (e-mail: adrod@med.up.pt)

Besides being the main storage site for the excess of energy, white adipose tissue also has important endocrine functions and strongly contributes to the inflammatory profile of obesity. TGF-β family members are key cytokines in inflammation and have multiple effects on adipose tissue. Circulating TGF-β levels correlate with obesity in mice and humans and recently it was shown that a systemic blockade of TGF-β/Smad3 signaling protects mice from diabetes and obesity. However, the importance of TGF-β/Smad3 signaling in the adipose tissue of obese humans is largely unknown being the focus of our work. Visceral and subcutaneous adipose tissue (VAT and SAT, respectively) samples were collected from patients undergoing gastric bypass surgery or laparoscopic cholecystectomy aging from 18 to 80 years-old. Body mass index (BMI) was calculated and ranged from 20 to 50 Kg/m^2^. Real-time PCR and Western blotting was employed to study TGF-β, Smad3, AKT and ERK1/2 expression and activation. Activation of Akt and Smad3 pathways in human VAT is associated to an increased fat mass. ERK1/2 phosphorylation is enriched in the VAT of overweight (25<BMI<30) individuals but it decreases to basal levels in obese patients (BMI>30). In both VAT and SAT, TGFβ mRNA levels were positively correlated with BMI. SAT of normoponderal and overweight individuals (BMI<30) were found to present lower levels of TGFβ mRNA when compared to visceral pads. This feature is blunted in the obese state (BMI>30). Akt and Smad3 activation increases with adiposity, most probably due to an enhanced TGF-β signaling. ERK1/2 activation seems to occur independently from TGFβ pathway but should be other important mechanism during weight gain. However, ERK1/2 signaling seems to be impaired in obesity, possibly because of desensitization mechanisms that are commonly found in chronic disorders.

**Acknowledgements:** Rodrigues A.R. is supported by POPH/FSE and Fundação para a Ciência e Tecnologia (FCT) (SFRH/BPD/92868/2013). UMIB is financed by FCT (PEst-OE/SAU/UI0215/2014)

### CO11 – The anti-obesogenic effect of OBEXâ on High Fat Diet fed mice and on 3T3-F442A cells

Sara Andrade, MSc^1,2^, Marcos C Carreira, PhD^1^, Begoña Cabia, MSc^1^, Ana B Crujeiras, PhD^1^, Maria Amil^1^, Mariana P Monteiro, MD, PhD^2^, Felipe F Casanueva, MD, PhD^1,3^. ^1^Ciber Fisiopatología de la Obesidad y Nutrición CB06/03, ^2^Anatomy Department, Instituto de Ciências Biomédicas Abel Salazar, University of Oporto, Portugal, ^3^Departamento de Medicina, Universidade de Santiago de Compostela, España; Complejo Hospitalario Universitario, SERGAS (e-mail: sarasousaandrade@gmail.com).

The prevalence of obesity has been increasing exponentially worldwide which caused the World Health Organization to classify the disease as the pandemic of the XXI century with important costs for medical systems. The need to develop new treatment targets for obesity is an emerging area of research since the therapeutic resources so far available, with the exception of bariatric surgery, have shown limited results. OBEX may be a new therapeutic approach for obesity. The effect of OBEX will be evaluated as a treatment for obesity in mice and also in *in vitro* models (3T3-F442A cells). 8 weeks old C57BL6 mice were divided in 5 groups (n = 10/group): 1 – HFD control mice, 2 – high fat diet + 0.10 g OBEX/day, 3 – high fat diet + 0.25 g OBEX/day, 4 – high fat diet + 0.50 g OBEX/day and finally 5 – Lean control mice. Mice were maintained for 2 months and food intake and body weight were accessed 3 times a week. Body composition was evaluated every 2 weeks by an Echo-MRI scan. On the eighth week of treatment, interscapular temperature was measured with an infrared camera and energy expenditure, spontaneous locomotor activity and respiratory quotient were measured in a 12-cage calorimetry system. Animals were sacrificed and organs recovered for posterior analysis. 3T3 cells were treated with OBEX during the differentiation process and also after the differentiation process was complete. The differentiation levels of the cells were evaluated by Oil Red staining and qRTPCR, using specific markers. Despite the fact that there were no significant differences in food intake between groups, mice treated with OBEX gained significantly less weight. This loss of weight was a result of a loss in fat mass. Mice presented an increase in metabolic rate, with increased interscapular temperature, energy expenditure and respiratory quotient. OBEX has significant dose-dependent inhibitory effects in the proliferation of 3T3-F442A pre-adipocytes, in the differentiation of 3T3 cells and it also acts on mature adipocytes reducing the lipidic load. OBEX appears to be efficient reducing weight gain on mice fed a high fat diet, and this is due to an inferior gain in fat mass and to an increase in energy expenditure. In addition, OBEX presents a triple effect on 3T3 cells, decreasing proliferation and differentiation and partially reversing the adipocyte phenotype, all of this in a dose-dependent manner.

Acknowledgments: CIBERobn, Catalysis

### CO12 –Vitamin D deficiency and secondary hyperparathyroidism are highly prevalent in the obesity and are aggravated by bariatric surgery

Ana C Carvalho, MD, MSc^1^, Tiago Guedes, MD, MSc ^1^, Tiago Morais, MSc^1^, Marta Guimarães, MD^2^, Mário Nora, MD^2^, Mariana P Monteiro, MD, PhD^1^. ^1^Department of Anatomy and UMIB (Unit for Multidisciplinary Research in Biomedicine) of ICBAS, University of Porto, Portugal, ^2^ Department of General Surgery, Centro Hospitalar de Entre o Douro e Vouga, Santa Maria da Feira, Portugal (e-mail: mpmonteiro@icbas.up.pt)

Vitamin D deficiency is a well-known cause of calcium and bone metabolism alterations, while more recently has also been implicated in deterioration of glucose homestasis and diabetes risk. Obesity is frequently associated with vitamin D deficiency and known to be aggravated by alterations of the gastro-intestinal tract leading to lipids malabsorption, with potential consequences for the target organs. The aim of this study was to evaluate and compare the prevalence of vitamin D deficiency and secondary hyperparathyroidism in obese patients subjected to two surgical variants of the gastric bypass, classical and metabolic. Additional aims were to evaluate the correlation between vitamin D and glycemic control, as well as, to compare the two technical variants of the surgery on weight loss, calcium and glycemic metabolism. Prospective analysis of anthropometric and biochemical parameters associated with calcium and blood glucose metabolism in obese patients that underwent gastric bypass surgery (n = 553) between 2009 and 2011. Patients were subjected to classic (n = 415) or metabolic (n = 138) gastric bypass and were evaluated up to 36 months after surgery. Before surgery, the prevalence of vitamin D deficiency and secondary hyperparathyroidism was 87.1% and 10.5%, respectively, figures that increased to 91.9% and 72.5% at 36 months after the procedure, with no differences between the two surgical techniques. Both procedures were equally effective in promoting sustained weight loss and long-term improvement in glycemic control. The vitamin D was negatively correlated with PTH, but no correlation was found between vitamin D and glucose or glycated hemoglobin. Obese patients have a high prevalence of vitamin D deficiency and secondary hyperparathyroidism that, if not corrected, worsens after gastric bypass surgery, with potential adverse effects on bone metabolism. Our study does not support the association between vitamin D and glycemic control.

**Acknowledgements:** UMIB is funded by grants from FCT (UID/Multi/00215/2013); SS Pereira holds a PhD grant from FCT (SFRH/BD/89308/2012).

### CO13 – Long biliopancreatic limb gastric bypass results in improved glycaemic control despite similar weight loss compared to the classical technique

Marta Guimarães, MD^2^, Ana Carvalho, MD, MSc^1^, Tiago Guedes, MD, MSc^1^, Tiago Morais, MSc^1^, Mário Nora, MD^2^, Mariana P. Monteiro, MD, PhD^1^. ^1^Department of Anatomy and UMIB (Unit for Multidisciplinary Research in Biomedicine) of ICBAS, University of Porto, Portugal, ^2^Department of General Surgery, Centro Hospitalar de Entre o Douroe Vouga, Santa Maria da Feira, Portugal (e-mail: martafilomenaguimaraes@gmail.com)

Bariatric surgery improves metabolic control or even induces type 2 diabetes (T2DM) remission. After having shown that 200 cm long biliopancreatic limb gastric bypass results in a higher than expected T2DM control, our aim was to compare the effects of this technical variant with the classic procedure. Patients submitted to classic gastric bypass (CGB) (n = 415) or long biliopancreatic limb gastric bypass (LLGB) (n = 138) for obesity treatment were monitored periodically up to 3 years after surgery. Obese patients, non-diabetic (n = 432) subjected either to CGB (n = 375) or LLGB (n = 57), and T2DM (n = 121) subjected to CGB (n = 40) or LLGB (n = 81), were similar at baseline regarding BMI distribution, although T2DM patients were significantly older (47.2±0.8 y vs 39.3±0.5, p<0.001). After surgery, non-diabetic patients displayed a significantly higher %EBMIL at 12 and 24 months when compared to T2DM, a difference that was still evident at 36 months after surgery, although not significant (87.58±1.21% (CGB) vs long limb 90.73±3.19% (LLGB) in non-diabetic patients vs 80.47±4.48% (CGB) vs 83.75±2.84% (LLGB) T2DM, p>0.05). There was no difference in the pattern of %EBMIL between the two surgical techniques among the different groups at all-time points assessed. Before surgery, fasting glucose and HbA1c was significantly lower in non-diabetic patients (HbA1c: 5.52±0.03% (CGB) and 5.91±0.21% (LLGB) vs 7.46±0.49% (CGB) and 7.27±0.23% (LLGB) in T2DM, p<0.001), although both parameters significantly decreased after surgery in all studied groups. Furthermore, LLGB in non-diabetics resulted in a significant decrease of HbA1c when compared to CGB (5.42±0.03% (CGB) vs 5.25±0.06% (LLGB), p<0.05), while in T2DM there was no difference in HbA1c between the two techniques (6.09±0.13% (CGB) vs 5.92±0.15% (LLGB)). %EBMIL was negatively correlated with HbA1c% in both groups, although this correlation was stronger in T2DM (ρ = -0.167 vs ρ = -0.339, p<0.01). Gastric bypass induced a significant improvement of glycemic control, regardless the technique performed that was mostly determined by the degree of EBMIL, particularly in T2DM. In non-diabetic patients, LLGB despite similar weight loss resulted in an additional decrease in HbA1c, when compared to the classical technique, suggesting the existence of additional mechanisms triggering the metabolic effects observed after the procedure.

**Acknowledgements:** UID/Multi/00215/2013

### CO14 – Plasma leptin response after chronic GLP-1 exposure is modulated by the integrity of the vagus nerve

Tiago Morais, MSc^1^, Sofia S Pereira, MSc^1^, Sara Andrade, MSc^1^, Ângela Moreira, MSc^1^, Duarte Monteiro^1^, Madalena Costa, BSc^1^, Bárbara Patrício^1^, Marcos Carreira, PhD^2^, Felipe F Casanueva, MD, PhD^2,3^, Mariana P Monteiro, MD, PhD^1^. ^1^Clinical and Experimental Endocrinology, Unit for Multidisciplinary Research in Biomedicine (UMIB), ICBAS, University of Porto, Portugal, ^2^CIBER de Fisiopatologia Obesidad y Nutricion (CB06/03), Instituto Salud Carlos III, Santiago de Compostela, Spain, ^3^Department of Medicine, USC University Hospital Complex, University of Santiago de Compostela, Santiago de Compostela, Spain (e-mail: mpmonteiro@icbas.up.pt).

Incretin based therapies are now widely used in clinical setting for type 2 diabetes treatment. Autonomic diabetic neuropathy can compromise the functional integrity of the vagus nerve, thus potentially impairing the action of the gut/brain axis hormones. The aim of this study was to evaluate the role of the vagal pathways in mediating the effects of chronic GLP-1 exposure in energy homeostasis.

Male Wistar rats submitted to truncal sub-diaphragmatic vagotomy (VGX) or sham procedure (SHAM) plus pyloroplasty, were randomized to receive GLP-1 (3.5 pM/min/Kg) or saline solution intraperitoneally through osmotic mini-pumps for 28 days (n = 5/ group). An additional group of SHAM rats (n = 6) was pair fed to the VGX-Saline (PF). Food intake and body weight were monitored daily, energy expenditure measured by indirect calorimetry, while epidydimal white adipose tissue (WAT) and interscapular brown adipose tissue (BAT) were weighed. Fasting of glucose, insulin, GLP-1, leptin and ghrelin plasma levels were measured by ELISA at the end of the experiment. Gene expression of NPY, AgRP, CART and POMC on the basal hypothalamus and UCP-1 in BAT were assessed by RT-PCR. GLP-1 levels were significantly higher in SHAM-GLP1 and VGX-GLP1 rats. VGX and PF rats had significantly lower food intake, body weight gain, percentage of WAT and BAT when compared to SHAM rats, but presented no difference in energy expenditure. Glucose levels were similar in all groups, but insulin and HOMA-IR were lower in VGX and PF, while PF rats also had a significantly higher ghrelin levels compared to Sham and VGX. No changes were observed in any of these parameters as result of GLP-1 exposure. Leptin levels were significantly lower in VGX and PF when compared to SHAM, while GLP1 exposure was responsible for an additional decrease in leptin levels compared to saline in VGX rats. No significant changes were observed in gene expression, despite SHAM-GLP1 rats had a 1.5 fold increase in anorexigenic neuropeptides when compared to SHAM rats, a trend that was absent in the VGX-GLP1 group. The vagus nerve seems to participate in the regulation of leptin levels in response to chronic GLP-1 exposure. Our data suggests that the implications of autonomic neuropathy, both in the central and peripheral effects of GLP-1 based therapies, ought to be further characterized to access the role of these drugs in this particular subset of patients.

Acknowledgments: UMIB is funded by grants from FCT (UID/Multi/00215/2013); SS Pereira holds a PhD grant from FCT (SFRH/BD/89308/2012).

### CO15 – Adipocyte secreted factors of obese individuals increase the metabolic activity of the adrenocortical tumor cells

Sofia S Pereira, MSc^1,2,3^, Joana R Pereira, BSc^3,4^, Ângela Moreira, MSc^3^, Marta Guimarães, MD^5^, Mário Nora, MD^5^, Duarte Pignatelli, MD, PhD^1,2,6^, Mariana P Monteiro, MD, PhD^3^. ^1^Instituto de Investigação e Inovação em Saúde, Universidade do Porto, Porto, Portugal, ^2^Institute of Molecular Pathology and Immunology of the University of Porto (IPATIMUP), Porto, Portugal, ^3^Clinical and Experimental Endocrinology, Department of Anatomy, Multidisciplinary Unit for Biomedical Research (UMIB), ICBAS, University of Porto, Porto, Portugal, ^4^Escola Superior de Tecnologia da Saúde do Porto do Instituto Politécnico do Porto (ESTSP-IPP), Vila Nova de Gaia, Portugal, ^5^Department of General Surgery, Centro Hospitalar de Entre o Douro e Vouga, Santa Maria da Feira, Portugal, ^6^Department of Endocrinology, Hospital S. João, Porto, Porto, Portugal (e-mail: mpmonteiro@icbas.up.pt)

Obesity is a recognized risk factor for several cancers and both conditions have been increasing dramatically in the last decades. More recently, the existence of an “adipoadrenal axis” has been hypothesized and some studies have already evaluated the possible role of adipokines in the modulation of adrenal function. However, the association between obesity and adrenocortical tumors has not been yet addressed. The aim of this study was to evaluate the influence of adipose tissue secreted factors on the biological behavior of adrenocortical carcinoma cells *in vitro*. Adrenocortical tumor cells H295R were incubated with secretomes of visceral adipose tissue obtained at elective surgery of normal weight and obese patients. Cell metabolic activity was evaluated by Alamar Blue Method, cell proliferation was assessed by the incorporation of BrdU, cell migration was studied using the Wound Healing Assay, invasiveness was tested using the Transwell systems and cellular adhesion through the expression of N-Cadherin by immunofluorescence and Western Blot. Adipose tissue secretomes of obese patients increased cell metabolic activity (p < 0.01) and changed the expression of the cell adhesion protein N-Cadherin. No effects were noted after incubation with the adipose tissue secreted factors in the proliferation, migration and invasion of adrenocortical carcinoma cells. This study demonstrates, for the first time, that adipose tissue secreted factors have direct influence in the metabolic activity of adrenocortical carcinoma cells, suggesting that obesity might also have a role in the modulation of the biological behavior of adrenal tumors.

Acknowledgments: IPATIMUP integrates the i3S Research Unit, which is partially supported by FCT; UMIB is funded by grants from FCT (UID/Multi/00215/2013); SS Pereira holds a PhD grant from FCT (SFRH/BD/89308/2012).

### P11 – Browning of white adipocytes: melanocortins as essential players

MJ Salazar, MSc^1^, AR Rodrigues, PhD^1^, S Rocha-Rodrigues, MSc^2^, IO Gonçalves, PhD^2^, H Almeida, PhD^1^, J Magalhães, PhD^2^, A M Gouveia, PhD^1,3^. ^1^Departamento de Biologia Experimental, FMUP – Faculdade de Medicina da Universidade do Porto, I3S – Instituto de Investigação e Inovação em Saúde, IBMC - Instituto de Biologia Celular e Molecular, Portugal, ^2^CIAFEL – Centro de Investigação em Actividade Física, Saúde e Lazer, FADEUP – Faculdade de Desporto da Universidade do Porto, Portugal, ^3^FCNAUP - Faculdade de Nutrição e Ciências da Alimentação da Universidade do Porto, Portugal (e-mail: agouveia@med.up.pt)

The discovery of beige or brown-like cells within white adipose tissue (WAT) depots in response to specific stimuli such as chronic cold exposure or β-adrenergic stimulation set off the conversion of white into brown adipocytes as an attractive option for controlling obesity (1). In white adipocytes, melanocortin neuropeptides induce lipolysis and inhibit fatty acid re-esterification by an ERK-dependent pathway (2). In brown adipocytes, melanocortins also promote thermogenesis (3). It was not yet described their role in the transdifferentiation of white to beige/brown adipocytes, thus being the aim of this study. Fully differentiated 3T3-L1 adipocytes were stimulated with the melanocortin α-MSH (1 μM) for 4 h or 24 h. Cells were also treated with 10 μM UO126 for 30 min, before α-MSH stimuli, for ERK1/2 inhibition. The expression of the browning hallmark genes uncoupling protein 1 (UCP-1) and peroxisome proliferator-activated receptor-gamma coactivator 1 alpha (PGC-1α) and the WAT characteristic gene resistin was determined by real time PCR. To evaluate the content of mitochondria, the binding of the fluorochrome 10-N-nonyl acridine orange (NAO) to mitochondria membrane was determined. In parallel, oxygen consumption measurements were also conducted in a Clark-type electrode. Real-time PCR results revealed an upregulation of UCP-1 and PGC-1α and a decreased expression of resistin gene after α-MSH treatment of 3T3-L1 adipocytes. Cell treatment with the ERK1/2 inhibitor prior to α-MSH stimuli had no effect on UCP-1 mRNA expression but attenuated the resistin downregulation. NAO fluorescence measurements revealed that melanocortin treatment increased the number of mitochondria. α-MSH treated adipocytes presented a significant increase in basal and oligomycin oxygen consumption rates. These results suggest that α-MSH prompts uncoupled respiration, which correlates with higher UCP-1 expression levels. The melanocortin α-MSH induces characteristic beige/brown adipocyte features in 3T3-L1 adipocytes, namely UCP-1 and PGC-1α increased expression and augmented mitochondria biogenesis and oxygen consumption.

**References:** (1) Wu, J., et al. (2012). Cell, 150(2), 366-376.

(2) Rodrigues, A. R., et al. (2013). BBA - Molecular and Cell Biology of Lipids, 1831(7), 1267-1275.

(3) Iwen, K. A., et al. (2008). J Endocrinol, 196(3), 465-472.

Acknowledgments: SFRH/BPD/92868/2013 and Tanita Healthy Weight Community Trust

### P12 – Higher endogenous leptin is associated with decreased epithelial cell nuclear density in the normal prostate gland

Pedro R Pereira^1^, Sofia S Pereira, MSc^1^, Ângela Moreira, MSc^1^, Madalena M Costa, BSc^1^, Ricardo Marcos, PhD^2^, Mariana P Monteiro, MD, PhD^1^. ^1^Department of Anatomy and UMIB (Unit for Multidisciplinary Research in Biomedicine) of ICBAS, University of Porto, Portugal, ^2^Laboratory of Histology and Embryology, Institute of Biomedical Sciences Abel Salazar, University of Porto, ICBAS, University of Porto, Portugal (e-mail: pedroreisper@gmail.com)

Leptin is an adipokine produced by the adipose tissue, usually in proportion to the fat tissue mass. Leptin receptors are found in the hypothalamus, where it participates in the regulation of energy homeostasis, but are also expressed in several other locations, such as the prostate gland, with a role not yet fully characterized. Although controversial, leptin was shown to increase proliferation in prostate cancer cells, representing a potential molecular link between obesity and cancer. The aim of this study was to evaluate the effect of different endogenous leptin levels on the morphology of the normal ventral prostate of adult mice. The prostate gland of 24 adult male mice divided into 4 groups, C57Bl6 normal weight and leptin mice, C57Bl6 mice with diet induced obesity, obese hyperleptinemic db/db mice and obese leptin deficient ob/ob mice (n = 6/group), was collected and paraffin embedded. The ventral prostate of Sirius Red stained slides underwent morphometric analysis to evaluate nuclear density, epithelial height, and internal and total acini areas. The plasma leptin levels were measured by Elisa. The prostate epithelial height was similar in all studied groups. Leptin deficient ob/ob mice had a higher epithelial nuclear density when compared with the other groups (p < 0.05). Leptin levels were negatively correlated with nuclear density (r2 = –0.580; p < 0.05), and the internal and total acini areas (r2 = –0.580 and r2 = –0.503, respectively; p < 0.05). This data suggests that different endogenous levels of leptin are able to influence the morphology of the normal prostate gland, even in the absence of neoplasia.

Fundings: SFRH/BD/89308/2012 and UID/Multi/00215/2013

### P13 – Prostate cancer cells are able to interact with surrounding adipocytes inducing phenotypic alterations that can promote tumor expansion

Ângela Moreira, MSc ^1^, Sofia S Pereira, MSc^1^, Sara Andrade, MSc^1,2^, Adriana Rodrigues, PhD^3^, Madalena Santos, BSc^1^, Tiago Morais, MSc^1^, Alexandra Gouveia, PhD^3,4^, Mariana P Monteiro, MD, PhD^1^ (e-mail: angelamoreiraarc@gmail.com)

Prostate cancer is the second most common cancer. Obesity has recently been implicated in the risk of prostate cancer development, tumor aggressiveness and increased mortality. The tumor microenvironment interaction with the surrounding tissues inducing phenotypic changes is one of the mechanisms that can promote tumor expansion and disease progression. Moreover, *in vitro* studies and clinical studies have suggested that tumor- surrounding adipocytes exhibit profound phenotypic changes that could facilitate tumor cell invasion and promote metastasis. After having showed that adipocyte secreted factors were able to enhance prostate cancer cell aggressiveness, the main propose of this research was to characterize the effect of prostate cancer cells in the adipocyte phenotype. RM1 prostate cancer cells were co-cultured with 3T3-L1 adipocytes using a Transwell culture system with 0.4 μm pore size PET membrane inserts, according with the manufactures instructions. Adipocytes or RM1 cells cultured alone in similar conditions were used as controls. The expression of adipocyte molecular markers, Glut4, PPARɣ and adiponectin, was evaluated by real-time PCR to assess the phenotypical profile of adipocytes. Cell co-culture media was collected for determination of glycerol levels using a commercially available kit and cytoplasm lipid droplets content was evaluated in order to assess delipidation of co-cultivated adipocytes. The co-culture of RM1 prostate cancer cells with differentiated adipocytes led to a significant decrease of Glut4 gene expression levels in the adipocytes (p < 0.05), while adiponectin and PPARɣ gene expression remained unchanged. No change was found in the amount of glycerol present in culture media, but there was a visible reduction in the size of lipid droplets in the adipocytes, a finding compatible with a delipidation state. Our results support the hypothesis of the existence of a cross-talk between prostate cancer cells and adipocytes, which promote adipocyte phenotypic changes towards dedifferention that could enhance tumor progression.

**Acknowledgements:** SFRH/BD/89308/2012; SPEDM grant and UID/Multi/00215/2013

### P14 – Lymph node metastases in thyroid carcinoma is independent of intratumoral lymphatic vessel density and correlates with extra thyroid extension of the tumor

Filipe Pereira, MSc^1^, Sofia S. Pereira, MSc^1^, Marta Mesquita, BSc^2^, Tiago Morais, MSc^1^, Madalena Costa, BSc^1^, Pedro Quelhas, PhD^3,4^, Carlos Lopes, MD, PhD^5^, Mariana P. Monteiro, MD, PhD^1^, Valeriano Leite, MD, PhD^6,7^. ^1^Clinical and Experimental Endocrinology, Unit for Multidisciplinary Research in Biomedicine (UMIB), ICBAS, University of Porto, Portugal, ^2^Serviço de Anatomia Patológica, Instituto Português Oncologia de Lisboa Francisco Gentil, Portugal, ^3^Instituto de Investigação e Inovação em Saúde (I3S), Universidade do Porto, Portugal, ^4^Instituto de Engenharia Biomédica (INEB), Universidade do Porto, Portugal, ^5^Instituto de Ciências Biomédicas Abel Salazar, Universidade do Porto, Portugal, ^6^Faculdade de Ciências Médicas, Universidade Nova de Lisboa, Portugal, ^7^Serviço de Endocrinologia, Instituto Português de Oncologia de Lisboa, Francisco Gentil, Portugal

Blood or lymph vascular invasion by tumor cells are well recognized markers of aggressiveness, as these are the common routes used for tumor spread and metastization. In the thyroid gland, different tumors tend to metastasize trough different vascular routes and with distinctive biological behavior, representing a good model to study the mechanisms of angiogenesis and lymphangiogenesis associated with the tumor progression, although its role has not yet been clarified. The aim of this study was to evaluate the lymph vessel density in the intra-tumoral tissue, peri-tumoral tissue and surrounding tumor-free thyroid tissue of patients harboring thyroid tumors, and their correlation with the pathological parameters and clinical characteristics of the patients. Histological sections of medullary carcinomas (n = 34) and papillary carcinomas, of the classic (n = 48) and follicular (n = 16) variants were stained for the lymphatic marker D2-40 by IHC. The stained area was quantified using a computer software for morphometric analysis (Image J – Fiji). The intra-tumoral lymphatic density was similar in the different studied groups and significantly lower than in the surrounding tumor-free thyroid tissue (p<0.001). There was no difference in the intra-tumoral lymphatic density between tumours with or without lymph node metastases. Medullary carcinomas presented a higher lymphatic density in the peri-tumoral and surrounding tumor-free thyroid tissue when compared to the classic variant of papillary carcinomas (p < 0.05). No significant differences were found in the peri-tumoral and surrounding tumor-free thyroid tissue lymphatic density between the two variants of papillary tumors. No correlations were found between the lymphatic density and the presence of extra-thyroid extension of the tumor that could justify the distinctive pattern of tumor spread observed for these two tumor variants. Lymph node metastases in both papillary and medullary thyroid carcinomas does not correlate with Intra-tumoral lymphatic vessel density, and is likely more dependent on the ability of the tumor to invade the abundant lymphatic vessel network than surrounds the tumor.

Fundings: SFRH/BD/89308/2012 and UID/Multi/00215/2013

### P15 – Effect of chronic glp-1 exposure on the morphology of pancreatic islets of intact and truncal vagotomized rats

Tiago Morais, MSc^1^, Bárbara Patrício, BSc^1^, Sofia S Pereira, MSc^1^, Madalena Costa, BSc^1^, Ângela Moreira, MSc^1^, Sara Andrade^1^, Duarte Monteiro^1^, Marcos Carreira, PhD^2^, Felipe F Casanueva, MD, PhD^2,3^, Mariana P Monteiro, MD, PhD^1^. ^1^Clinical and Experimental Endocrinology, Unit for Multidisciplinary Research in Biomedicine (UMIB), ICBAS, University of Porto, Portugal, ^2^CIBER de Fisiopatologia Obesidad y Nutricion (CB06/03), Instituto Salud Carlos III, Santiago de Compostela, Spain, ^3^Department of Medicine, USC University Hospital Complex, University of Santiago de Compostela, Santiago de Compostela, Spain (e-mail: mpmonteiro@icbas.up.pt)

GLP-1 based therapies have been introduced more than a decade ago in the clinical practice for the treatment of T2DM with proven metabolic benefits. However, recent data has suggested that despite the good side effect profile, these drugs could have unpredicted effects in the pancreatic morphology towards increased risk of neoplasia, raising concerns about their safety. Male Wistar rats (n = 30) were subjected to truncal sub-diaphragmatic vagotomy plus pyloroplasty surgery (VGX n = 7) or simulated vagotomy surgery plus pyloroplasty (SHAM n = 5). VGX and SHAM animals were randomized to receive exogenous administration of GLP-1 (3.5 pM/min/Kg) for 28 days delivered through a peritoneal implanted mini-pump (SHAM GLP-1 n = 5 and VGX GLP-1 n = 5) or serve as controls. An additional group of SHAM rats was pair fed (PF) to the VGX rats (n = 6). Food intake and body weight were monitored daily. At the end of the experiment, fasting levels of glucose, total and active GLP-1, insulin, leptin and ghrelin were measured by ELISA. Pancreas were collected, routinely processed for histology and IHQ stained for insulin, glucagon and ki-67 and IF stained for glucagon and ki-67. Langerhans islets were identified, photographed and the specific antigen staining was quantified using computer software for morphometric analysis (Image J). GLP-1 plasma levels of GLP-1 pump implanted SHAM rats was significantly higher than controls. Although food intake and body weight gain were lower in VGX when compared to SHAM rats, and similar to PF rats, GLP-1 exposure did not alter food intake in either of the groups. There were no differences in fasting glucose levels between the groups, but insulin and HOMA-IR were lower in PF rats. Compared to SHAM rats, VGX rats had lower leptin levels and PF rats had higher ghrelin levels. Morphologic analysis of the pancreatic tissue revealed no abnormalities in neither of the studied groups. The size of the Langerhans islets and the percentage of stained area for insulin, glucagon and ki-67 were similar in all experimental groups. Chronic low dose GLP-1 exposure showed no evidence of morphologic alterations in the pancreatic tissue of intact or truncal vagotomized non- diabetic rats.

Fundings: SFRH/BD/89308/2012; POCTI/FEDER Fcomp-01-0124-FEDER- 015896 and UID/Multi/00215/2013

## GENETICS

### CO16 – Long-term Follow-up of 132 Patients With Phenylketonuria: potential effects of Phe levels on neuropsychological development

Carla M Carmona, PhD^1,2^, Manuela F Almeida, BSc^1,2^, Ana M Fortuna, MSc^1,2^. ^1^Centro de Genética Médica Doutor jacinto Magalhães - Centro Hospitalar do Porto, EPE, Porto, Portugal, ^2^Unit for Multidisciplinary Research in Biomedicine, Abel Salazar Institute of Medical Sciences, University of Porto, UMIB/ICBAS/UP, Porto, Portugal (e-mail: carla.carmona@chporto.min-saude.pt)

Neurological disability caused by untreated Phenylketonuria (PKU) can be largely prevented by an early and adequately dietary treatment. However, a slight intellectual quotient (IQ) decrease together with impairments in specific cognition aspects, including executive function deficits, may persist even in well treated patients. The main objective of this study is to characterize our PKU patients in terms of their neuropsychological development and to understand the way that this chronic condition can affect their performance in different contexts of life. We studied 132 early diagnosed patients aged 1 to 34 years. Neonatal screening blood phenylalanine (Phe) concentrations, used to classify the disease severity, and the quality of dietetic control (QDC) defined as the annual medians of blood Phe, were considered independent variables. Patient's outcome was evaluated according to the global DQ/IQ value at different group of ages, IQs subscales profile, educational level, as well as their professional career and socio-affective behaviour. In this patient group, the results of IQ/DQ evaluation showed global values below the reference population norm in all age groups considered. We also found a specific profile of neurocognitive and behavioural difficulties. These difficulties were both negatively and significantly correlated with the QDC and influenced their school progress, professional success and treatment adherence. The results of school performance and professional career reflected these results with patients with bad dietetic control having a greater need for adapted *curriculum* and lower educational and professional formation levels. We found a specific neurocognitive profile and difficulties in their psychosocial behaviour suggesting the need of a special supervision throughout life. This study illustrates the advantage of a multidisciplinary team involved in the treatments of PKU patients, in order to greatly improve their quality of life.

### CO17 – Origin of normal-size *FMR1* alleles without AGG interspersions

Nuno Maia, MSc^1,2∗^, Joana Loureiro, MSc^1,3∗^, Bárbara Oliveira, MSc^2,4^, Isabel Marques, PhD^1,2^, Rosário Santos, PhD^1,2^, Sandra Martins, PhD^5^, Paula Jorge, PhD^1,2^. ^1^Unidade de Genética Molecular, Centro de Genética Médica Jacinto de Magalhães, Centro Hospitalar do Porto, CHP, E.P.E., Porto, Portugal, ^2^Unidade Multidisciplinar de Investigação Biomédica – UMIB – FCT, ^3^I3S- Instituto de Investigação e Inovação em Saúde/IBMC-Instituto de Biologia Molecular e Celular, Universidade do Porto, Porto, Portugal, ^4^Max-Planck-Institut für Experimentelle Medizin Klinische Neurowissenschaften, Göttingen, Germany, ^5^i3S-Instituto de Investigação e Inovação em Saúde/IPATIMUP-Institute of Molecular Pathology and Immunology of the University of Porto, Porto, Portugal (e-mail: nuno.maia@chporto.min-saude.pt) ^∗^Equal contributors

Fragile-X Syndrome (FXS), the most common known cause of inherited intellectual disability, is mainly caused by a hypermethylation and expansion of the *FMR1* CGG repeat to over 200 triplets (full-mutation). Owing to the heterogeneity of full mutations in somatic cells, size and methylation mosaics, incomplete/absence of methylation and the lyonization phenomenon of females, the FXS molecular diagnosis is not always a straightforward task, is methodologically very demanding and requires expertise in result interpretation. The presence of alleles with various *FMR1* CGG repeat sizes or differences in the extent of methylation, resulting in genetically heterogeneous cellular populations, are phenomena believed to occur post-zygotically, and known as size- or methylation-mosaics, respectively. Although it has been previously described, mosaicism of two *FMR1* alleles in males is a particularly rare finding. Even rarer is the occurrence of more than two alleles or the simultaneous presence of expanded and normal alleles, in a unique tissue. In the course of the routine *FMR1* analysis, four mosaic cases with a normal and a full-mutation allele were identified in blood samples of 4 male patients. In a preliminary analysis, these normal-sized alleles revealed no AGG interruptions and seem to have been originated through a postzygotic retraction event. Herein, arguments in favour and against the hypothesis that uninterrupted normal-sized alleles can be originated on full-mutation contraction will be explored. Further 212 control and 123 unrelated FXS male samples were studied for haplotype analysis and comparison. Polymorphic markers flanking the *FMR1* repeat were used, including four STRs: DXS998, DXS548, FRAXAC1 and FRAXAC2 and three intragenic SNPs: rs971000, rs29282 and rs25715. Although some variability was found on each SNP lineage, T-T-T (present in 62.5% of control alleles and 10.5% of FXS alleles) was the only consistent with an ancestral origin for FXS. Normal alleles in mosaic cases have different CGG triplet-sizes and belong to two SNP lineage: (CGG)_18_-C-C-C, (CGG)_26_-T-T-T, (CGG)_35_-T-T-T and (CGG)_39_-C-C-C. Interestingly two of the four independent contraction events from expanded alleles have occurred on the same haplotypic background (A21). Our results suggest that the most frequent origin for normal pure alleles does not seems to be by contraction of expanded alleles but this hypothesis cannot be ruled out in case a single ancestral event occurred.

### CO18 – Morphological and Proteomic Studies on Smith-Lemli-Opitz syndrome: further evidence that cholesterol deficiency causes autophagy

ML Cardoso MSc^1,2^, R Vitorino PhD^3^, R Pinto Leite PhD^4^, R Arantes-Rodrigues PhD^5,6^, R Fernandes PhD^2^, C Amaral PhD^1^, C Bernardo MSc^7^, H Reguengo MSc^1,8^, R Ferreira PhD^3^, F Amado PhD^3^, F Marques PhD^1,2^. ^1^Department of Biological Sciences, Faculty of Pharmacy, University of Porto, Portugal, ^2^Institute for Molecular and Cell Biology (IBMC), University of Porto, Portugal, ^3^QOPNA, Mass spectrometry center, Department of Chemistry, University of Aveiro, Portugal, ^4^Genetic Service, Cytogenetic Laboratory, Hospital Center of Trás-os-Montes e Alto Douro, ^5^Department of Veterinary Sciences, University of Trás-os-Montes and Alto Douro, Vila Real, Portugal, ^6^Center for the Study of Animal Sciences (CECAV), University of Trás-os-Montes and Alto Douro, Vila Real, Portugal, ^7^Experimental Pathology and Therapeutics Group, Research Center, Portuguese Oncology Institute, Porto, Portugal, ^8^Department of Clinical Chemistry, Porto Hospital Center, Portugal (e-mail: marialuis.cardoso@gmail.com)

Cholesterol is an important structural component of cellular membranes and it was found to play an essential role on embryogenesis and development. In the present investigation we used fibroblasts from Smith-Lemli-Opitz syndrome patients (SLOS) - a metabolic genetic disease affecting cholesterol biosynthesis pathway as a model to study the effect of cholesterol deficiency on human cells. Fibroblasts from SLOS patients and normal human controls were cultivated simultaneously in both standard conditions and cholesterol depleted media. Cells were submitted to optical and electron microscopy, monodansylcadaverine staining, evaluation and quantification of acridine orange acidic vacuoles, LC3 immunocytochemistry, TUNEL assay and proteomic analysis by iTRAQS LC-MALDI-TOF/TOF–MS. Morphological studies showed that when endogenous synthesis of cholesterol is inadequate (SLOS) and there is no appropriate supply to overcome cellular needs (cholesterol depleted media), cell proliferation *in vitro* becomes impaired and autophagy is activated. It is known that many of the postnatal clinical problems of patients with SLOS are considered as direct consequence of the inability to produce large amounts of cholesterol needed for growth. It is possible that during some periods of life autophagy plays an important role promoting tissue turnover and keeping a slower growth velocity. In fact, an idiopathic hypermetabolism was identified in some patients that could be related with the catabolic process of autophagy. Since activation of autophagy seems to be a self-rescue mechanism of the SLOS cells, we further investigated if there was also changes in protein expression which support surviving cell adaptive modifications. Such cells in cholesterol depleted medium showed an overexpression of a set of proteins. Mainly, there was an increase in MnSOD expression to combat oxidative stress derived from the accumulation of 7-dehydrocholesterol, (caused by the inherited enzymatic deficiency) and thus control of cell proliferation, whereas heat shock 70 kDa protein 4, an autophagic protein (Atg2), also presents a cytoprotective activity and inhibits apoptosis. The mechanism by which SLOS fibroblasts handle their metabolic deficit involves autophagy which plays an important role in cell survival.

**Acknowledgements:** This work was supported by Portuguese Foundation for Science and Technology (FCT), European Union, QREN, FEDER and COMPETE for funding the QOPNA research unit (project PEst-C/QUI/UI0062/2011) and CENTRO-07-ST24- FEDER-002034.

### CO19 – Mandibulofacial Dysostosis (Treacher Collins Syndrome) – novel de novo mutation in TCOF1

Márcia Martins, MD^1^, Pedro Botelho, DS^2^, Marta Souto, MSc^2^, Regina Arantes, PhD^2^, Osvaldo Moutinho, MD^1^, Rosário Pinto-Leite, PhD^2^. ^1^Genetics Clinic, Centro Hospitalar Trás-os-Montes e Alto Douro, Vila Real, Portugal, ^2^Cytogenetics Laboratory, Centro Hospitalar Trás-os-Montes e Alto Douro, Vila Real, Portugal, ^3^Gynecology/Obstetrics Service, Centro Hospitalar Trás-os-Montes e Alto Douro, Vila Real, Portugal (e-mail: marciam@chtmad.min-saude.pt)

Mandibulofacial Dysostosis or Treacher Collins syndrome (TCS) is a well known syndrome resulting from a congenital disorder of craniofacial development with a combination of bilateral symmetrical oto-mandibular dysplasia and various head and neck defects. The facial dysmorphism is characteristic and includes bilateral and symmetrical hypoplasia of the malar bones and infra-orbital rim (80% of cases) and of the mandible (78%) (retrognathia, retrogenia). External ear malformation such as microtia or anotia, atresia of the external auditory canal and anomalies of the ossicular chain are often present (60%) with bilateral conductive hearing loss. First described in 1900, the estimated incidence is 1/50000 live births, with 60% of the cases resulting from a *de novo* mutation. *TCOF1* gene mutations, with an autossomic dominant transmission, are the most common cause of the disorder, accounting for 81 to 93 % of all cases. *POLR1C* and *POLR1D* gene mutations are responsible for an additional 2 % of cases. The present work describes a new *TCOF1* mutation in a 26 yr old TCS patient, in a study done in order to provide prenatal diagnosis in a future pregnancy. PCR amplification and DNA analysis through sequencing of all exons of *TCOF1* gene and the contiguous intronic sequences were performed. A new mutation c.2507del(p.Pro836Glnfs^∗^37) in heterozygosity in *TCOF1* gene was detected. A review of etiology, clinical features, differential diagnosis and genetic counselling is presented.

### CO20 – Medical Students’ Opinions concerning Genetic Tests, Genetic Diagnosis and related Bioethical Problems: a survey during the academic year 2014-2015

Natália Oliva-Teles, PhD^∗1,2^, Carla Carmona, PhD^∗1,2^, Rui Nunes, MD, PhD^3^, Ana Fortuna, MSc, MD^1,2^. ^1^Centro de Genética Médica Doutor Jacinto Magalhães (CGMJM)/Centro Hospitalar do Porto, E.P.E. (CHP), Porto, Portugal, ^2^Unidade Multidisciplinar de Investigação Biomédica (UMIB), Porto, Portugal, ^3^Department of Social Sciences and Health (DCSS), Faculty of Medicine, University of Porto, Portugal (e-mail: natalia.teles@chporto.min-saude.pt) ^∗^shared authorship

The extensive diversity of available genetic tests (GTs), which contribute to the analysis of hereditary characteristics, employs several techniques and is used for the diagnosis of many genetic abnormalities. These techniques, of growing sophistication and detail, allow increasingly accurate clarification of unclear medical conditions but frequently produce “unexpected results” (URs) or variants of unknown clinical significance. The diverse ethical problems that relate to GTs, their results, and deciding who may, and/or should be informed of any findings represent serious challenges to health professionals. Medical students (197) attending the fourth year of a six year *Integrated Master degree In Medicine,* from the Faculty of Medicine of the University of Porto, Portugal, volunteered to provide their opinions on 13 questions of a self-administered questionnaire concerning genetic tests, genetic diagnosis and related bioethical issues, while also supplying their demographic/social data. The statistical analysis of results was done using SPSS, version 20. Main questionnaire results showed: genetic counselling both before (70.6%) and after (77.0%) GTs was considered very important; it was important to further investigate URs in all situations (64.2%), although only sometimes (58.4%) would a more precise clinical situation be beneficial; URs should more importantly be transmitted to users in case of Mendelian disease (42.1%) and affected carriers (37.9%). Also, the main concern as future professionals dealing with URs is users’ anxiety (82.7%) and the user, if a competent adult, should decide if or when to carry out genetic tests (70.3%). The mean age of students was 22,3 [20-42], mostly females (65.8%). With this longitudinal investigation made during the 2014/2015 academic year, the authors (a multidisciplinary team including medical doctors, specialists in genetics, bioethicists and psychologists) emphasize the importance of bioethics studies as useful tools for medical training, particularly on theoretical case scenarios, in order to help students on decision-making in their future medical practice. Further research on these subjects, using similar questionnaires, will also be completed by a variety of health care professionals, with and without specialized genetic background, to assess whether there are significant differences before and after formal education in genetics and bioethics.

### CO21 – The genetics of hereditary myopathies revisited by massive parallel sequencing

Jorge Oliveira, MSc^1,2^, Conceição Egas, PhD^3^, José C. Machado PhD^4,5^, Mário Sousa MD, PhD^2,6,7∗^, Rosário Santos PhD^1,2,8∗^. ^1^Unidade de Genética Molecular, Centro de Genética Médica Dr. Jacinto Magalhães, Centro Hospitalar do Porto. Porto, Portugal, ^2^Unidade Multidisciplinar de Investigação Biomédica (UMIB), Instituto de Ciências Biomédicas Abel Salazar (ICBAS), Universidade do Porto. Porto, Portugal, ^3^Unidade de Sequenciação de Genomas, Centro de Neurociências e Biologia Celular da Universidade de Coimbra, UC-Biotech, Parque Tecnológico de Cantanhede, Cantanhede, Portugal, ^4^Instituto de Patologia e Imunologia Molecular da Universidade do Porto. Porto, Portugal, ^5^Faculdade de Medicina da Universidade do Porto. Porto, Portugal, ^6^Departamento de Microscopia, Laboratório de Biologia Celular, Instituto de Ciências Biomédicas Abel Salazar (ICBAS), Universidade do Porto. Porto, Portugal, ^7^Centro de Genética da Reprodução Prof. Alberto Barros. Porto, Portugal, ^8^UCIBIO/REQUIMTE, Departamento de Ciências Biológicas, Laboratório de Bioquímica, Faculdade de Farmácia, Universidade do Porto. Porto, Portugal (e-mail: jorgemsmoliveira@gmail.com) ^∗^These authors contributed equally to this work

Over the last few years the unremitting increase of new *loci* associated with hereditary myopathies (HM) had a profound effect on classification and diagnostics. Massive parallel sequencing (MPS) significantly contributed towards these changes by providing new alternatives to the conventional approaches, enabling higher throughput and lower costs. Further developments in MPS are still required for its full translation into the clinic, not only in technological aspects but essentially in data analysis and interpretation. An overview of our current research in HM using MPS is presented. Two different MPS strategies were used for mutation screening: i) a gene panel to study 20 *loci* implicated in congenital myopathies and ii) whole-exome sequencing (WES) analyzing ∼20,000 genes. A total of 15 genetically unsolved patients with HM were selected for the study: ten cases were analyzed by the CM gene panel and five by WES (two trios - patient and both parents - and three single exomes). Bioinformatic tools were used for variant annotation, filtering and homozygosity mapping. Expression analysis at the RNA level was performed to clarify the impact of new variants. Overall, ∼118 000 distinct sequence variants were generated in this project; 7% are not listed in dbSNP (Single Nucleotide Polymorphism Database). Thirteen mutations in the *CHKB*, *NEB*, *RYR1*, *SEPN1* and *TTN* genes were successfully identified in eight patients (seven analyzed by the gene panel and one by WES). Eight mutations were not previously reported. Four variants required further studies and were found to have an effect on mRNA splicing. Only three patients analyzed by the CM gene panel had no mutations identified. WES analysis of four cases is still being carried out. Technical and analytical pitfalls of MPS that may give rise to false negative results were also identified. This research contributed towards the molecular characterization of patients with rare HM, some remained undiagnosed for several years. An additional important aspect is the enormous amount of genetic data generated. Considering that there is no national consortium committed to identify rare genetic variants specific to our population, this data is a highly valuable asset for research and diagnostic purposes. Besides promoting such pioneering work within our research unit, the short-term goal is to lay the foundations for new sequencing capacities, future genomics research and improved diagnostics.

**Acknowledgements:** To all clinicians that collaborated in this work, especially Dr. Ricardo Taipa, Dr. Manuel Melo Pires, Dr. Manuela Santos and Dr. Teresa Coelho (Centro Hospitalar do Porto), and Dr. Luís Negrão and Dr. Isabel Fineza (Centro Hospitalar Universitário de Coimbra). Also thanks to Hugo Froufe (Biocant) and Dr. José Luís Costa (IPATIMUP). JO is a recipient of a PhD scholarship attributed by “Fundo para a Investigação e Desenvolvimento do Centro Hospitalar do Porto”. The work was financed by the Institutions of the authors and in part by UMIB, which is funded by National Funds through FCT Foundation for Science and Technology, under the Pest-OE/SAU/UI0215/2014.

### CO22 – Difficult genetic counseling in a subtelomeric syndrome

Rosário Pinto-Leite, PhD^1^, Marta Souto, MSc^1^, Pedro Botelho, DS^1^, Regina Severo, MD^2^, Osvaldo Moutinho, MD^2^, Márcia Martins, MD^3^. ^1^Cytogenetics Laboratory, Centro Hospitalar Trás-os-Montes e Alto Douro, Vila Real, Portugal, ^2^Gynecology/Obstetrics Service, Centro Hospitalar Trás-os-Montes e Alto Douro, Vila Real, Portugal, ^3^Genetics Clinic, Centro Hospitalar Trás-os-Montes e Alto Douro, Vila Real, Portugal (e-mail: mlleite@chtmad.min-saude.pt)

The 3p deletion syndrome (OMIM 613792) is a rare disorder caused by deletions of varying lengths in the 3p25→pter region. The phenotypes of individuals with these deletions range from normal to severe. Despite the low number of patients described (less than 50), different phenotypes have been described, such as mental retardation, developmental delay, intrauterine growth restriction, micro and brachycephaly. Most cases are *de novo*, although a few familial cases have been reported. Intense research to determine which genes in the regions 3p25 and 3p26 cause the features of the syndrome has been performed but it remains unknown. The authors present a family (mother and two children) with a 3p deletion. The 3p deletion was detected in a prenatal diagnosis of the third child. Cytogenetic analysis showed a female karyotype with a subtelomeric deletion on the short arm of chromosome 3, confirmed by FISH technique (subtelomeric probes for chromosome 3). Cytogenetic and FISH study were carried out to the couple and another child. The same subtelomeric deletion was found in the mother and son and was confirmed by array analyses that revealed a deleted segment of 7,4Mb in size involving 18 genes (*CHL1, CNTN6, CNTN4, CNTN4-AS2, IL5RA, TRNT1, CRBN, LRRM1, SETMAR, SUMF1, ITPR1, EGOT, LOCI00507582, BHLHE40, ARL8B, EDEM1, MIR4790, IGRM7*) in 3p26.3p26.1, in the proband, mother and child. The mother has no health problems and no learning difficulties and the daughter (6 years old) shows a normal development. The son at 11 years old presented a mild mental retardation. This family is followed at genetic consultation. This is another family with a heredity del 3p26, that presents the clinical variability of syndrome 3p, ranging from the complete absence of phenotypic changes until mild mental retardation. Every new case should be reported in order to improve the genetic counseling.

### CO23 – Beware of fabry disease diagnosis – insights from an atypical portuguese family

Isaura Ribeiro, MSc.^1,2^, Elisabete Silva, BSc.^1^, Eugénia Pinto, MSc.^1^, Fernanda Pinto, BSc.^1^, Sónia Rocha, MSc.^1^, Célia Ferreira, BSc.^1^, Helena Ribeiro, BSc^1^, Sandra Jacinto, MD^3^, Diana Antunes, MD^4^, Ana C. Ferreira, MD^5^, Dulce Quelhas, MSc.^1,2^, Francisco Laranjeira, MSc.^1^, Carla Caseiro, MSc.^1^, Lúcia Lacerda, PhD^1,2^. ^1^Centro de Genética Médica Doutor Jacinto Magalhães, Centro Hospitalar do Porto, EPE, Porto, Portugal, ^2^Unidade Multidisciplinar de Investigação Biomédica, Instituto de Ciências Biomédicas Abel Salazar, Universidade do Porto, ^3^Serviço de Neurologia Pediátrica, Hospital Dona Estefânia, Centro Hospitalar de Lisboa, Central, EPE, Lisboa, Portugal, ^4^Serviço de Genética Médica, Hospital Dona Estefânia, Centro Hospitalar de Lisboa Central, EPE, Lisboa, Portugal, ^5^Unidade de Doenças Metabólicas, Hospital Dona Estefânia, Centro Hospitalar de Lisboa Central, EPE, Lisboa, Portugal (e-mail: isaura.ribeiro@chporto.min-saude.pt)

Fabry disease (FD, MIM # 301500) is a treatable X-linked inherited lysosomal storage disorder of glycosphingolipid catabolism, due to deficient or absent activity of the lysosomal enzyme α-galactosidase A. The enzymatic defect leads to progressive lysosomal accumulation of the neutral sphingolipids, mainly globotriaosylceramide and globotriaosylshingosine, in most organs. FD is a chronic progressive condition with symptoms such as pain in the extremities, heat and cold intolerance, corneal changes and angiokeratoma, often beginning in childhood. Progressive renal insufficiency and cardiovascular involvement are the main causes of premature death. Reported male patients have partial reduction or absence of α-galactosidase A activity. In female carriers α-galactosidase A activity may range from zero to control values, due to X-linked inheritance phenomena so, molecular genetic tests are mandatory for FD diagnosis in females. Over 770 pathogenic mutations found in *GLA* gene have been associated with FD. This work highlights the need for a multidisciplinary approach in the diagnosis of FD providing clinical, biochemical and molecular tests results. FD diagnosis underlay in three approaches: α-galactosidase A activity measured in capillary dried blood spots, peripheral blood plasma and total leukocytes; GB3 urinary excretion measured in 24 hour urine and genotype analysis by *GLA* gene exons and exon-intron boundaries sequencing. This work reports clinical, biochemical and molecular data of an atypical Fabry family, in which affected males, hemizygous for a pathogenic mutation of *GLA* gene, c.827G > A (p.S276N), have reduced levels of α-galactosidase A activity in leukocytes, however they present normal α-galactosidase A activity in plasma. FD diagnosis is not straight forward through α-galactosidase A enzymatic activity, and frequently requires a combination of different technical approaches, even in male patients. Caution must be taken regarding screening methods, such as those conducted on plasma α-galactosidase A activity, even in patients with early onset of the disease, because of their pitfalls. Preliminary results indicate that p.S276N is associated with the classic phenotype of FD. Identification of a α-galactosidase A mutation associated with a clinically relevant phenotype would be extremely useful for disease progression evaluation, as well as for enzyme replacement therapeutic decisions.

**Acknowledgements:** Clementina Maia for secretarial assistance.

### CO24 – Lack of association of asap1 gene and tuberculosis in chinese

Nelson Tang, MD, FRCPA^1^, Amy Wang, Mphil^1^, CC Leung, MBBS, FHKAM, FFPH^2^, KC Chang, MBBS, MSc, FHKAM^2^, M Hui, MD, FRCPath^3^, CY Chan, PhD. ^1^Department of Chemical Pathology, The Chinese University of Hong Kong, ^2^Department of Health, Hong Kong SAR Government, ^3^Department of Microbiology, The Chinese University of Hong Kong (e-mail: nelsontang@cuhk.edu.hk)

Susceptibility to tuberculosis (TB) varies between different people. Genetics may play an important role in this susceptibility as single gene defects had been documented in familial TB. However, it has been more difficult to identify genes accounting for it in the general population. Recently, several candidate gene studies and genome wide association studies have been reported. For example, we and our collaborators (1) found that CISH gene encoding for cytokine inducible SH2-containing protein were associated with risk of TB. In the latest GWAS, ASAP1 gene was reported to account for susceptibility to TB. Therefore, we attempted to replicate these findings in Chinese patients. A sample of 1147 Chinese TB patients and 1191 Chinese controls were genotyped for a tagging SNP in the ASAP1 gene (rs4733775). It is a frequent SNP with allelic frequency of 0.38 in Chinese population. Analysis of both allelic (MAF 0.4 in both case and control groups) and genotype frequencies showed that there was no association with TB. Although ASAP1 was reported to have a robust association with TB in the original paper studying Russian patients, its association in the African sample was weak. Here, we suggest that the role of this gene in the Chinese population is also weak. We are continuing to genotype additional SNPs in this gene to confirm this point.

**References:** (1) Khor CC, et al. CISH and susceptibility to infectious diseases. N Engl J Med. 2010 362(22):2092-101.

**Acknowledgements:** This work was supported by Health and Medical Research Fund 14130282 of the HKSAR Government

### P16 – Beta choline kinase deficiency: a rare cause of muscular dystrophy, cardiomyopathy and intellectual disability

Jorge Oliveira, MSc^1,2^, Luís Negrão MD, PhD^3^, Isabel Fineza MD^4^, Ricardo Taipa MD^5^, Manuel Melo-Pires MD, PhD^5^, Ana Rita Gonçalves MSc^1,2^, Emília Vieira MSc^1,2^, Márcia E. Oliveira PhD^1,2^, Ana Maria Fortuna MD^2,6^, Mário Sousa MD, PhD^2,7,8∗^, Rosário Santos PhD^1,2,9∗^. ^1^Unidade de Genética Molecular, Centro de Genética Médica Dr. Jacinto Magalhães, Centro Hospitalar do Porto. Porto, Portugal, ^2^Unidade Multidisciplinar de Investigação Biomédica (UMIB), Instituto de Ciências Biomédicas Abel Salazar (ICBAS), Universidade do Porto. Porto, Portugal, ^3^Consulta de Doenças Neuromusculares, Hospitais da Universidade de Coimbra, Centro Hospitalar Universitário de Coimbra. Coimbra, Portugal, ^4^Unidade de Neuropediatria, Centro de Desenvolvimento da Criança Luís Borges, Hospital Pediátrico de Coimbra, Centro Hospitalar Universitário de Coimbra. Coimbra, Portugal, ^5^Unidade de Neuropatologia, Centro Hospitalar do Porto. Porto, Portugal, ^6^Serviço de Genética Médica, Centro de Genética Médica Dr. Jacinto Magalhães, Centro Hospitalar do Porto. Porto, Portugal, ^7^Departamento de Microscopia, Laboratório de Biologia Celular, Instituto de Ciências Biomédicas Abel Salazar (ICBAS), Universidade do Porto. Porto, Portugal, ^8^Centro de Genética da Reprodução Prof. Alberto Barros. Porto, Portugal, ^9^UCIBIO/REQUIMTE, Departamento de Ciências Biológicas, Laboratório de Bioquímica, Faculdade de Farmácia, Universidade do Porto. Porto, Portugal (e-mail: jorgemsmoliveira@gmail.com) ^∗^These authors contributed equally to this work

Muscular dystrophies (MD) are a diagnostically challenging group of muscle diseases, especially considering the expanding clinical and genetic heterogeneity (57 different *loci* associated). Over the last two decades, we successfully identified genetic defects in several hundred patients/families contributing towards a genetic epidemiological sketch of MD in Portugal. Considering the remarkable developments in massive parallel sequencing technology, we aim to increase the knowledge on MD genetics and to translate this research into future diagnostics. A patient [with a childhood-onset MD, intellectual disability (ID) and dilated cardiomyopathy] and her parents were subjected to whole-exome sequencing (WES), on a non-optical semi-conductor sequencing system. Data analysis resorted to different bioinformatic algorithms to filter and weigh the impact of novel variants. To obtain further insight into the epidemiology of beta choline kinase (Chkb) deficiency, a further 8 patients presenting both MD and ID were selected from our laboratory cohort, as follows: 3 with congenital MD (out of 45 undiagnosed cases), 3 with limb-girdle MD (out of 250 cases) and 2 with Duchene/Becker MD (out of 186 cases). WES analysis, focusing on the genes implicated in myopathies and MD, and assuming an autosomal recessive disease model, identified a single novel silent homozygous variant in the choline kinase beta gene (*CHKB*). Further data analysis obtained for *CHKB* gene, led to the identification of a donor splice site mutation: c.1031+3G > C. This mutation was confirmed by Sanger sequencing in the patient (homozygous) and parents (heterozygosity). Additional studies performed at the mRNA level demonstrated its pathogenicity; no normal *CHKB* transcripts were detected in the patient. Analysis of the other 8 selected patients failed to identify mutations in the *CHKB* gene. The rare association between ID and MD has been previously linked to defective alfa-dystroglycan glycosylation pathway and also a subset of Dystrophin (*DMD*) gene mutations affecting the brain *DMD* isoforms. As shown here, Chkb deficiency should be considered as an additional culprit. Our study, although restricted to a small number of patients, suggests that *CHKB* mutations are an extremely rare cause of MD in Portugal, which is consistent with the scarcity of cases reported worldwide.

**Acknowledgements:** The authors wish to thank Hugo Froufe and Dr. Conceição Egas (Biocant). JO is a recipient of a PhD scholarship attributed by “Fundo para a Investigação e Desenvolvimento do Centro Hospitalar do Porto”. The work was financed by the Institutions of the authors and in part by UMIB, which is funded by National Funds through FCT Foundation for Science and Technology, under the Pest-OE/SAU/UI0215/2014.

### P17 – Screening for *MED12* variants in Opitz-Kaveggia and Lujan-Fryns syndromes

Cathy Paulino, BSc^1,2,∗^, Isabel Marques, PhD^1,∗^, Raquel Chaves, PhD^2^, Paula Jorge, PhD^1^, Rosário Santos, PhD^1^. ^1^Unidade de Genética Molecular, Centro Genética Médica Doutor Jacinto Magalhães, Centro Hospitalar do Porto - EPE, Porto, Portugal; Unidade Multidisciplinar de Investigação Biomédica (UMIB), Instituto de Ciências Biomédicas Abel Salazar (ICBAS), Universidade do Porto, Porto, Portugal, ^2^University of Trás-os-Montes and Alto Douro (UTAD), Department of Genetics and Biotechnology (DGB), Laboratory of Cytogenomics and Animal Genomics (CAG), Vila Real, Portugal (e-mail: isabel.medeiros.marques@gmail.com) ^∗^Equal contributors.

Opitz-Kaveggia (FGS; OMIM#305450) and Lujan-Fryns syndromes (LFS; OMIM#309520) are rare forms of X-linked Intellectual Disability (XLID), mostly affecting males. Patients show a distinctive “peculiar” facial appearance, with high and prominent forehead, frontal hair upsweep and small prominent ears with simplified helical pattern characteristic of FGS and tall narrow face typical of LFS. LFS patients can also have tall marfanoid stature, macrocephaly, long hands with hyperextensible digits and mild general hypotonia. Mild to moderate intellectual disability and several behavioural problems, e.g. hyperactivity, affability, and excessive talkativeness, are commonly present in both syndromes. *MED12* belongs to the trinucleotide repeat containing family of genes and is localized at Xq13. This gene encodes for the mediator of RNA polymerase II transcription subunit 12, an essential subunit of this mediator complex implicated in various processes that are relevant for transcription, including the organization of chromatin architecture. MED12 protein is implicated in different developmental pathways and is involved in the regulation of neuronal gene expression. As MED12 malfunction can alter cell-fate decisions, it is not surprising that mutations can lead to a variety of pathologic conditions including cancer and developmental disorders. Few *MED12* mutations were found to be the cause of LFS and FGS. In LFS a single recurrent mutation c.3020A > G in exon 22 has been reported, while in FGS two frequent mutations both in exon 21, have been described: c.2873G > A and c.2881C > T. Recently, a novel *MED12* missense mutation, c.5922G > T, was implicated in non-syndromic XLID in three brothers. The aim of this work was the screening for *MED12* gene variants in 43 male patients by PCR-amplification followed by Sanger sequencing. In order to determine the significance of the variants found, in-silico studies using bioinformatics tools were carried out. Results reveal the presence of several variants of uncertain significance (VUS), but none of the documented recurrent mutations. Furthermore, the majority of found variants are located in intron regions, being only four exonic variants. In order to determine the pathogenicity of the thirteen VUS found, several analysis were carried out. Here we discuss the putative pathogenic effect of such variants. Co-segregation studies and mRNA analysis were also performed in order to study the impact on protein expression.

**Acknowledgements:** This work was supported by Portuguese Foundation for Science and Technology (FCT) and the Department of Education, Training and Research from Centro Hospitalar do Porto (DEFI-CHP), Portugal.

### P18 – Case report: A double translocation found in prenatal diagnosis

Rosário Pinto-Leite, PhD^1^, Pedro Botelho, DS^1^, Marta Souto, MSc^1^, Regina A Rodrigues, PhD ^1^, Cátia Carnide, MD^2^, Osvaldo Moutinho, MD^2^, Márcia Martins, MD^3^. ^1^Cytogenetics Laboratory, Centro Hospitalar Trás-os-Montes e Alto Douro, Vila Real, Portugal, ^2^Gynecology/Obstetrics Service, Centro Hospitalar Trás-os-Montes e Alto Douro, Vila Real, Portugal, ^3^Genetics Clinic, Centro Hospitalar Trás-os-Montes e Alto Douro, Vila Real, Portugal (e-mail: mlleite@chtmad.min-saude.pt)

Constitutional balanced rearrangements are present in approximately 0.2% of the population. The most frequent are translocations with an incidence of 1 person in 500. They may pass undetected through generations, but miscarriages, infertility or the birth of a child with an unbalanced form of translocation usually reveals the existence of a familial chromosomal translocation. The improvement of new techniques, such as array Comparative Genomic Hybridization (aCGH), has increased the resolution of novel or rare microdeletions/microduplications, but chromosome analysis remains the gold standard to detect structural chromosome rearrangements. The authors present a case of a 45-year-old pregnant woman referred for the first time to our prenatal center. She had a previous history of ten miscarriages, not investigated, and five healthy children. Amniotic fluid (two cultures) and parent's blood cultures were performed according to routine protocols established in the laboratory. Oligonucleotide array-CGH was applied. Cytogenetic analysis of amniotic fluid revealed a double translocation: t(1;2)(p34.1;p23)mat and a *de novo* t(1;5)(q10;q10). aCGH analysis did not detected any gain or loss involving the translocated segments and the ultrasound parameters were normal. The couple decided to continue the pregnancy. Cases of unrelated double translocations are extremely rare, there are only seven cases described and six of them were *de novo*. The presence of an inherited and a new translocation detected in a prenatal diagnosis implies a more careful approach in the pregnancy follow up. Currently prenatal diagnosis can benefit from the advances of the molecular techniques that are very useful for the precise characterization of chromosomal anomalies.

**Acknowledgements:** The authors will present a review of the previous described cases and compared with this case.

### P19 – Importance of cytogenetic study in multiple myeloma

Rosário Pinto-Leite, PhD^1^, Marta Souto, MsC^1^, Pedro Botelho, DS^1^, Manuel Cunha, MD^2^, Osvaldo Moutinho, MD^3^, Márcia Martins, MD^3^. ^1^Cytogenetics Laboratory, Centro Hospitalar Trás-os-Montes e Alto Douro, Vila Real, Portugal, ^2^Hematology Service, Centro Hospitalar Trás-os-Montes e Alto Douro, Vila Real, Portugal, ^3^Gynecology/Obstetrics Service, Centro Hospitalar Trás-os-Montes e Alto Douro, Vila Real, Portugal, ^4^Genetics Clinic, Centro Hospitalar Trás-os-Montes e Alto Douro, Vila Real, Portugal (e-mail: mlleite@chtmad.min-saude.pt)

In Multiple Myeloma (MM) there is a high genetic instability resulting in a wide variety of genetic and chromosomal abnormalities. MM is a malignancy disorder resulting from the proliferation of a single clone of plasma cells, derived from B cells in the bone marrow. Cytogenetics abnormalities are considered an important prognostic indicator and are observed in 30-50% of the cases. Monosomy or deletions of chromosome 13, translocations involving the immunoglobulin heavy chain locus and ploidy status are the most frequent findings. The present report concerns the results of a cytogenetic study in a seventy three years old male patient with MM. Bone marrow cell culture and GTL banding was done according to routine protocols in the laboratory. Cytogenetic analysis was performed following the standard cytogenetic guidelines. FISH painel (13q-, 17p-, t(4;14), t(11;14)) was applied. The karyotype revealed a 47, XY, +8, t(6;19)(p21.3:p13.3), t(11;14)(q13;q32) [16] / 46, XY [3]. FISH was positive to t(11;14) (88%). In this patient besides the characteristic anomaly of MM (t(11;14)), trisomy 8 - a rare event in lymphoid neoplasia, and a translocation not described in the literature, were also found. FISH is a sensitive method to investigate cytogenetics of MM but it should always be performed along with conventional cytogenetics analysis. They are important to predict treatment responses and prognosis.

### P20 – FMR1 gene-zygosity discrimination: the use of hrMCA in female samples

Stephanie Salgado, BSc^1^, Nuno Maia,MSc^1,2^, Eduardo Cruz, BSc^3^, Isabel Marques, PhD^1,2^, Rosário Santos, PhD^1,2^, Paula Jorge, PhD^1,2^. ^1^Unidade de Genética Molecular, Centro de Genética Médica Jacinto de Magalhães, Centro Hospitalar do Porto, CHP, E.P.E., Praça Pedro Nunes, 88, 4099-028, Porto, Portugal, ^2^Unidade Multidisciplinar de Investigação Biomédica – UMIB/ICBAS/UP, ^3^Bioportugal Quimico-Farmacêutica, LDA, Rua do Campo Alegre, 1306, 2° - sala 208, 4150-174, Porto, Portuga (e-mail: jorgpaula@gmail.com)

Fragile X syndrome (FXS; MIM #600624) is the most common form of inherited intellectual disability and autism, very frequently caused by an expansion to over 200 CGG repeats, located in the 5’ UTR of the *FMR1* gene. This expansion, named full mutation, causes *FMR1* gene silencing due to methylation of its CGG repeats and upstream CpG islands, and consequent absence of its protein product. Several PCR based kits have been developed to aid in diagnosis of fragile X syndrome and fragile X associated disorders, e.g. tremor and ataxia syndrome (FXTAS; MIM #300623) and primary ovarian insufficiency (FXPOI), through determination of CGG repeat length up to 200 CGG. For detection of alleles greater than 200 CGG and assessment of methylation status, Southern Blot is still considered the gold standard technique. Nevertheless, both methodologies have limitations: Southern Blot is very time-consuming and requires a large amount of intact, high-molecular weight DNA and PCR-based methods hardly candiscriminate between an homozygous (>20% female samples) and a carrier of a premutation or full mutation due to preferential amplification of the smaller (normal-sized) allele. The current workflow for fragile X clinical testing at our laboratory includes a PCR screening in blood samples collected on FTA filter paper (internal quality control) in parallel to the routine methodology. The aim of this work was to test the High Resolution Melting Curve Analysis (hrMCA) technique in gDNA obtained from dried blood spots, using the FastFraX FMR1 Identification Kit (Biofactory). For this work we tested ten gDNA samples belonging to females previously characterized at the molecular level in our laboratory: normal homozygous (n = 5), fully mutated (n = 4), pre-mutated (n = 1). As anticipated, this kit cannot determine the number of CGG repeats in the *FMR1* gene, neither the DNA methylation state, or distinguish between pre-mutated and fully mutated alleles, but can accurately identify (homo)zygosity in female samples through the analysis of a characteristic melting curve profile. Consequently, hrMCA seems a suitable technique for large scale routine testing, as a rapid and inexpensive screening approach. Although using a very small number of samples, these preliminary results allow the tempting conclusion that hrMCA can be used as an alternative to replace cumbersome Southern Blot in zygosity discrimination.

### P21 – Familial subtelomeric rearrangement involving chromosomes 2 and 16 detected by array-cgh and molecular cytogenetics.

AR Soares, MD^1^, G Soares, MD^1^, C Candeias, MSc^2^, MM Freitas, MSc^2^, NO Teles, PhD^2^, AM Fortuna, MSc^1^, ML Silva, MSc^2^. ^1^Department of Medical Genetics, Centro de Genética Médica Dr. Jacinto de Magalhães/Centro Hospitalar do Porto, Porto, Portugal, ^2^Cytogenetics Unit, Centro de Genética Médica Dr. Jacinto de Magalhães/Centro Hospitalar do Porto, Porto, Portugal (e-mail: ana.rita.soares@chporto.min-saude.pt)

Several molecular and cytogenetic techniques have been used in order to help doctors with genetic diagnosis, namely in developmental delay and dysmorphic syndromes. We present a case of a 13 months-old girl referred to our Centre with global developmental delay and dysmorphisms, in whom two pathogenic CNV were detected by array-CGH resulting from a cryptic chromosomal rearrangement in the mother. Clinical case: 13 months-old infant female, only child of an unrelated couple, with two previous pregnancy losses (one miscarriage and one termination of a foetus with Trisomy 21). No other relevant family history was referred. Although the pregnancy was uncomplicated, prenatal diagnosis was performed due to maternal age and the foetal karyotype was normal (46,XX). The child presented with hypotonia, global developmental delay, facial dysmorphisms (high forehead, small upslanting palpebral fissures, small nose and mouth, flat philtrum), cerebral ventricular dilatation and malrotation of the left kidney. Because clinical findings were non-specific array-CGH was requested. Array-CGH: two copy number alterations found in the proband were known to be associated with developmental delay and dysmorphisms - 16p13.3 duplication (involving the CREBPP gene) and 2q37.3 deletion. These anomalies were confirmed by MLPA techniques in the patient. Parents’ cytogenetic studies, including FISH, were performed and showed a balanced reciprocal translocation between 2q37.3 and 16p13.3 subtelomeric regions in the mother; the father had normal results. Array-CGH is very useful as the first line study for children presenting with developmental delay and dysmorphisms. However, this technique can only detect quantitative chromosomal anomalies. This case reveals the importance of complementing array-CGH with molecular cytogenetic techniques (namely FISH) in cases where a familial structural rearrangement is suspected. The detection of structural rearrangements has important implications in genetic counselling of families as they increase the recurrence risk for chromosomal imbalances.

### P22 – Genetically engineered construction and creation of a recombinant adenovirus as an approach for gene therapy of dysferlinopathies.

Starostina IG, MSc^1^, Solovyova VV, PhD^1^, Rizvanov AA, PhD^1^, Dr. Sci, Deev RV, PhD^2^. ^1^KFI – Kazan (Volga region), Federal University, OpenLab “Gene and Cell Technology”, 420008, Kazan, Russia, ^2^KFI – Kazan (Volga region), Federal University, Institute of Fundamental Medicine and Biology, 420008, Kazan, Russia (e-mail: fairin@mail.ru)

Dysferlinopathies are a heterogenous group of autosomal recessive inherited muscular dystrophies caused by mutations in DYSF gene. They include a wide spectrum of muscle diseases. The onset of the disease is in the late teens or early adulthood. Dysferlinopathy is characterized by muscle weakness, high serum creatine kinase levels and a prominent inflammatory infiltrate. Dysferlin is mainly expressed in skeletal muscle and in monocytes, and patients display a severe reduction or absence of this protein in both tissues. There are no effective treatments for dysferlinopathy nowadays. The potential to restore defective protein by introducing into the cell a functional wild-type gene is a promising method of gene therapy of muscular dystrophies. Since the size of codon-optimized dysf gene is large (6243 bp) adenoviral vectors are considered suitable for creation genetic constructions. The vectors are capable to deliver a large amount of recombinant genetic information in dividing and nondividing cells, and to provide high expression level of transgenes. The aim of our work was the creation of recombinant adenovirus encoding a codon-optimized human dysferlin gene and analysis of the protein expression in cell culture *in vitro*. During the work there were performed different methods which were mainly composed of: preparation of the genetic construction pAd-Dysf by subcloning codon-optimized cDNA of dysf gene into a recombinant adenoviral vector pAd/CMV/V5-DEST, transfection of cells HEK293A with plasmid pAd-Dysf. Immunofluorescent analysis of HEK293A cells showed a positive reaction with a rabbit polyclonal antibody to dysferlin. Western blot analysis of protein lysates revealed the presence of bright strips corresponding to the expected molecular weight of dysferlin protein (237kDa). It was obtained a recombinant replication-defective adenovirus Ad5-Dysf and to obtain the virus in high concentrations was also carried out a ultracentrifugation of Ad5-Dysf in cesium gradient. Onwards, was performed genetic modification of HEK293A with obtained recombinant adenovirus. Further immunoassays of transduced cells showed a positive reaction with a rabbit polyclonal antibody to dysferlin protein. Obtained plasmid and viral constructions will be applied as an approach of gene therapy for dysferlinopathy. In prospect, we planned different experiments with peripheral blood monocytes (PBMs) and experiments on animal models for this kind of orphan diseases.

## IMMUNITY AND INFLAMMATION

### CO25 – DODAB: Monolein based liposomes as novel nanocarriers for vaccine design against *Candida albicans* infections

Catarina Carneiro, PhD^1^, Alexandra Correia, PhD^2^, Tânia Lima, BSc^1^, Manuel Vilanova, PhD^2,3^, Célia Pais, PhD^1^, Andreia C Gomes, PhD^1,5^, M Elisabete CD Real Oliveira, PhD^4,5^, Paula Sampaio, PhD^1^. ^1^Centre of Molecular and Environmental Biology (CBMA), Department of Biology, University of Minho, Braga, Portugal, ^2^Instituto de Investigação e Inovação em Saúde, Universidade do Porto, and IBMC - Instituto de Biologia Molecular e Celular, Universidade do Porto, Porto, Portugal, ^3^Instituto de Ciências Biomédicas de Abel Salazar (ICBAS), Universidade do Porto, Porto, Portugal, ^4^Centre of Physics (CFUM) University of Minho, Campus of Gualtar, Braga, Portugal, ^5^NanoDelivery I&D in Biotecnology, Biology Department, Campus of Gualtar, Braga, Portugal (e-mail: psampaio@bio.uminho.pt)

Prevention of systemic infections caused by *Candida* species has become of paramount importance since this type of infection represents the fourth leading cause of nosocomial bloodstream infection in modern hospitals. Formulating protein antigens into nanocarriers has emerged as one of the most promising strategies to trigger an immune response against the target antigens. Here, we describe the preparation and characterisation of Antigen Delivery Systems (ADS) composed by DODAB: Monoolein (MO)-based liposomes loaded with *C. albicans* cell wall surface proteins (CWSP) and demonstrate their adjuvant potential and use in antigen delivery. DODAB: MO liposomes were prepared using the lipid film hydration method followed by incubation with DTT extracted CWSP from *C. albicans* yeast cells. BALB/c bone marrow derived dendritic cells (BMDC) were stimulated with ADS, CWSP or empty liposomes (EL) for 6 or 24 h and surface activation markers were evaluated. BALB/c mice were injected thrice subcutaneously, either with ADS, CWSP or EL, with a 2-week intervening period, and 3 weeks after the last immunization mice were infected intravenously (i.v.) with C*. albicans* yeasts or sham-infected with PBS and humoral and cellular immune responses were evaluated. These ADS assembled as stable negatively charged spherical nanoparticles with a mean size of 280 nm. ADS caused significantly higher activation of BMDC than CWSP alone, as revealed by enhanced expression of activation surface markers. In the mouse model, immunization with ADS induced strong antibody responses and cell-mediated immunity. Moreover, in contrast to immunization with CWSP alone, ADS induced polarization towards a Th17-type immune response, known to be protective in candidiasis, and led to the production of specific antibodies recognizing different proteins from those induced by CWSP immunization. Moreover, ADS immunization conferred protection against a lethal *C. albicans* systemic infection. In conclusion, DODAB:MO-based liposomes loaded with *C. albicans* proteins have an excellent immunogenic potential and can be explored for the development of an immunoprotection strategy against *Candida* infections.

**Acknowledgements:** This work was supported by FEDER through POFC – COMPETE and by national funds from FCT through grants PEst-OE/BIA/UI4050/2014, PEst-C/FIS/UI0607/2013 (CFUM) and PTDC/QUI/69795/2006, while Catarina Carneiro holds a fellowship SFRH/BD/69068/2010. We acknowledge NanoDelivery-I&D em Bionanotecnologia, Lda. for access to their DLS.

### CO26 – Distinct immune response but similar innervation and molecular profiles in human hip osteoarthritis and implants aseptic loosening

Daniel M Vasconcelos, MSc1,2,3^∗^, Manuel R Silva, MD^1,2,4,5∗^, António Mateus, MD^4,5^, Juliana Alves, PhD^1,2^, Gil C Machado, MSc^1,2^, Joana M Santos, MSc^1,2^, Diogo P Carvalho, MSc^1,2^, Inês S Alencastre, PhD^1,2^, Rui Henrique, MD, PhD^3,6^, Gilberto Costa, MD^4,5^, Mário A Barbosa, PhD^1,2,3^, Meriem Lamghari, PhD^1,2∗^. ^1^Instituto de Investigação e Inovação em Saúde, Universidade do Porto, Portugal, ^2^Instituto de Engenharia Biomédica, Universidade do Porto, Portugal, ^3^Instituto Ciências Biomédicas Abel Salazar, Universidade do Porto, Portugal, ^4^Serviço de Ortopedia e Traumatologia, Centro Hospitalar São João, Portugal, ^5^Faculdade de Medicina, Universidade do Porto, Portugal, ^6^Instituto Português de Oncologia do Porto, Portugal (e-mail: lamghari@ineb.up.pt) ^∗^These authors contributed equally to this work

Major efforts from clinical research, tissue engineering field and material sciences to fundamental bone biology are undertaken to provide solutions to overcome hip joint failure. Osteoarthritis and aseptic loosening share inflammation and pain as sign and symptom. Although both conditions have been widely addressed, the immune and innervation profiles in these hip pathologies remain unclear and their interplay is poorly explored. Herein, inflammation and tissue innervation as well as associated local mediators were simultaneously studied in defined hip joint microenvironment underlying osteoarthritis and aseptic loosening. Tissue organization, innervation, immune cell distribution and profile were analyzed in synovial tissues retrieved from osteoarthritis (n = 15) and aseptic loosening patients (n = 21). In the latter, the accumulation of prosthetic debris in synovial-like tissues was also evaluated. The local mediators profile was assessed analyzing the gene expression levels of inflammatory cytokines and innervation markers. The hypothesis that local biological response modulates circulating immune cell populations and systemic TGF-β1 levels was evaluated by pre-operative leukograms and ELISA. Histopathological analysis revealed differences regarding tissue architecture and immune cell profile in synovial tissues of osteoarthritis and aseptic loosening patients. Interestingly, both scenarios presented similar nerve fibers density and anatomical distribution. Moreover, among prosthetic debris, ZrO_2_ particles were highly present in cemented loosed implants but no correlation was found between particles type and macrophage inflammatory profiles. Remarkably, osteoarthritis and aseptic loosening patients presented similar gene expression patterns but a significant decrease of TGF-β1 was found in synovial-like tissues but not in the serum of aseptic loosening patients. Hip osteoarthritis and aseptic loosening share similar patterns of innervation and of inflammatory mediators’ expression, despite the differences at tissue and cellular levels. These findings highlight the need to study other markers likely to be involved in these pathologies, and which may constitute promising candidates for therapies to improve hip joint lifetime and pain relieve.

**Acknowledgements:** This work was supported by Portuguese funds through FCT in the framework of project PTDC/BIM-MED/1047/2012 to DCP and post-doc fellowships SFRH/BPD/63618/2009, SFRH/BPD/75285/2010 and SFRH/BD/87516/2012 to CJA, ISA and DMV, respectively.

### CO27 – New insights into the impaired t-cell function in diabetic foot ulcerations

João Moura, PhD^1^, João Rodrigues, MSc^2^, Marta Gonçalves, BSc^2^, Cláudia Amaral, BSc^2^, Margarida Lima, MD, PhD^2^, Eugénia Carvalho, PhD^1^. ^1^Center for Neuroscience and Cel Biology (CNC), Coimbra, Portugal, ^2^Hospital de Santo António, Centro Hospitalar do Porto (HSA-CHP), Porto, Portugal (e-mail: jmouraalves@gmail.com)

Chronic diabetic wounds do not progress through the different phases of wound healing in synchrony due to diabetes-associated neuropathy, microangiopathy and impaired immune function. Because we still do not clearly understand how the immune response is impaired in diabetic foot ulcers (DFU), treatment has relied on lower limb amputation and occasional new strategies with limited results. Prevention is still essential for DFU care, but has been limited because, despite having already identified various DFU risk factors, we are still unable to predict the development of DFU and are thus in urgent need of a good biomarker that signals individuals at risk or in the earliest DFU stages. Using a different approach to this problem, we analyzed how diabetes in general and DFU in particular affect TCR diversity, by PCR based studies, and the relative distribution of the most representative T-cell populations (naive, activated/memory and effector), by flow cytometry. Our results show that diabetes has a profound impact on the circulating T-cell pool, lowering naïve T-cell numbers and potentiating the accumulation of effector T-cells to a point where TCR diversity may become an issue to the immune response. TNF-α, that is largely secreted by effector T-cells, has been shown to have various harmful effects on wound healing, from the inhibition of fibroblast and keratinocyte proliferation and migration to the induction of apoptosis in endothelial cells and pericytes, through the increase in the expression of the FOXO1 transcription factor. In conclusion, our results may explain the self-sustaining systemic inflammatory environment that is driven by the accumulation of effector cells. Since the accumulation of effector T-cells seems to be in the center of DFU pathology, immunotherapeutic strategies should be devised in order to diminish T-cell activation and tissue accumulation. Moreover, effector T-cell numbers and the overall TCR-Vβ repertoire diversity seem to have prognostic value in the treatment of DFU and possibly other diabetes-associated complications. A larger study is being devised to ascertain the potential of the use of T-cell population numbers and TCR-Vβ repertoire diversity as a biomarker of DFU.

### P23 – Flow cytometry for the diagnosis of hereditary deficiency of platelet dense granules

Catarina Lau, MD^1,3^, José Carlos Ferreira^1,4^, Marta Gonçalves, BSc^1,3^, Marco Sampaio, MD^2,3^, Sara Morais, MD^2,3^, Manuel Campos, MD^2,3^, Margarida Lima, MD, PhD^1,3^. ^1^Laboratory of Cytometry and ^2^Thrombosis and Haemostasis Unit, Department of Clinical Hematology, Hospital de Santo António, Centro Hospitalar do Porto, Porto, Portugal, ^3^UMIB/ICBAS/UP, ^4^Biochemistry student, FCUP (e-mail: mcatarinalau@gmail.com)

Platelet storage pool diseases (PSPD) are rare disorders characterized by a variable reduction in the number or the contents of platelet dense and alpha-granules, as well as combined defects. The most common disorder is isolated dense granule deficiency, which may occur as a single abnormality or as part of inherited syndromes such as the Chediak-Higashi or Hermansky-Pudlak Syndrome (HPS). The standard tests to diagnose are lumi-aggregometry (LA) and electron microscopy (EM), the latter being time consuming and often not available in clinical laboratories. Mepacrine is a fluorescent acridine derivative which binds with high affinity to adenine nucleotides and is rapidly and selectively taken up by platelet dense granules. Here, we tested the possibility of diagnose PSPD by evaluating the platelet mepacrine-uptake, using flow cytometry. We first studied the platelet mepacrine uptake in 8 healthy individuals (blood donors). Acquisition was made immediately after incubation (T0), 1.5 hours (T1) and 3 hours thereafter (T2). Next, we studied two patients with the diagnosis of HPS confirmed by LA and EM and two other patients with platelet dysfunction without deficiency of platelet dense granules, as evaluated by EM. A dual labelling (mepacrine and anti-CD42b-PE) protocol, in whole blood, was used. Mean fluorescence intensity (MFI) of mepacrine-associated fluorescence was evaluated. Results are expressed as mean ± standard deviation of the MFI. The MFI decreased from T0 (6.88 ± 2.22) to T1 (5.63 ± 1.97) and almost stabilized from T1 to T2 (5.43 ± 1.71). The time point chosen for analysis was T1. Platelets of the 2 patients with HPS (decreased number of platelet dense granules on EM) showed less mepacrine uptake (2.70 and 2.19) then platelets from healthy individuals (n = 6) analyzed in the same day (4.60 ± 0.43). The other two patients studied, who did not have deficiency of platelet dense granules on EM, showed a normal mepacrine uptake (4.13 and 3.97). Our results suggest that platelet mepacrine-uptake evaluated by flow cytometry is a simple, easy and rapid method for the identification of platelet dense granule deficiencies. Further studies are necessary in order to validate the use of this method for the diagnosis of these rare platelet disorders in clinical practice, which can potential be done in resting or stimulated platelets, identifying both patients with defects in number or release of platelets dense granules contents.

### P24 – Mechanical isolation and flow cytometry characterization of endothelial cells

Cláudia Torres^1,2^, Rui Machado, MD^3^, Margarida Lima, MD, PhD^2,4^. ^1^PhD student in Biomedical Sciences, ICBAS/UP, Portugal, ^2^UMIB/ICBAS/UP, ^3^Department of Angiology and Vascular Surgery and ^4^Laboratory of Cytometry, Department of Clinical Hematology, Hospital de Santo António, Centro Hospitalar do Porto, Porto, Portugal (e-mail: torres.cb@gmail.com)

Endothelial cells (EC) line the inside of the blood vessels, having vital functions in the various physiological events, and being involved in different pathologies. Most of the studies on EC have been made by histology and immunohistochemistry on veins, or performing cultures of EC extracted using enzymatic methods. It is known that these techniques have disadvantages, sometimes masking the expression of cellular receptors. The use of fixatives and enzymes can change the chemical properties of cells, wielding deleterious effects on their receptors. The purpose of this study is to describe a simple and efficient mechanical method to isolate EC from human veins, and to identify them by flow cytometry. Segments of saphenous veins with length varying from 1.5 to 4 cm were obtained from 10 patients submitted to varicose vein surgery. Veins were opened longitudinally, the vascular surface lumen was exposed upwards, and their extremities were fixed with needles to a square of Styrofoam previously covered with Parafilm^®^, being added drops of phosphate buffered saline (PBS, pH 7.2) containing 2% (w/v) bovine serum albumin (BSA) (PBS-2%BSA). Afterwards, the EC were mechanically removed with two serrated forceps, by gently squeezing in continuous movement from the vessel wall. Then the cells were suspended in PBS-2%BSA, aspirated with a Pasteur pipette and transferred into a 15 ml conical polypropylene tube. After the isolation procedure, cells were stained with anti-CD146, anti-CD45 and anti-CD31 mouse anti-human monoclonal antibodies (mAbs), using a wash-stain-lyse-no-fix-no-wash procedure. Furthermore, to confirm the correct identification of EC, additional stains were made using anti-CD309, anti-CD105 and anti-CD144 mAbs. Endothelial cells were easily identified as being CD45-CD31+CD146+. The median percentage of EC found in the 10 samples analyzed was 9.6% (range: 8.0% to 27.8%) of total events. Additional stain for the CD309, CD105 and CD144 molecules assured the correct identification of the EC. With this method we were able to isolate and to characterize a well-defined EC population. In the future we intend to use these mechanically isolated EC to make a broad type of studies, such as a meticulous phenotypic characterization of the EC for the expression of molecules involved in activation, angiogenesis and apoptosis, as well as *ex-vivo* functional studies aimed to test the effect of different stimuli on the EC.

**Acknowledgements:** The authors thank to the medical doctors, nurses and technicians from the Laboratory of Cytometry / Department of Hematology and Department of Angiology and Vascular Surgery that contributed for this study. They also thank to Enzifarma for sponsor of the anti-CD31 antibody and to the Unit for Multidisciplinary Research in Biomedicine of the Institute for Biomedical Sciences Abel Salazar, University of Porto (UMIB/ICBAS/UP) for financial support.

### P25 – A decrease in inflammation associates with an improvement in iron status in obese children and adolescents

Susana Coimbra, PhD^1,2^, Cristina Catarino, PhD^1,3,4^, Henrique Nascimento, PhD^1,3,4^, Ana Inês Alves^3,4,5^, Ana Filipa Medeiros^5^, Elsa Bronze-da-Rocha, PhD^1,3,4^, Elísio Costa, PhD^1,3,4^, Petronila Rocha-Pereira, PhD^6^, Luísa Aires, PhD^5,7^, André Seabra, PhD^5^, Jorge Mota, PhD^5^, Helena Ferreira Mansilha, MD^8^, Carla Rêgo, MD, PhD^9^, Alice Santos-Silva, PhD^1,3,4^, Luís Belo, MD, PhD^1,3,4^. ^1^UCIBIO\REQUIMTE, Department of Biological Sciences, Laboratory of Biochemistry, Faculty of Pharmacy, University of Porto, Porto, Portugal, ^2^CESPU, Institute of Research and Advanced Training in Health Sciences and Technologies (IINFACTS), Gandra-PRD, Portugal, ^3^IBMC-Instituto de Biologia Molecular e Celular (Institute for Molecular and Cell Biology), Universidade do Porto, Porto, Portugal, ^4^Instituto de Investigação e Inovação em Saúde (Institute for Research and Innovation in Health), Universidade do Porto, Porto, Portugal, ^5^Research Centre in Physical Activity, Health and Leisure – CIAFEL, Faculty of Sport, University of Porto, Porto, Portugal, ^6^Health Science Research Centre, University of Beira Interior, Covilhã, Portugal, ^7^University Institute of Maia – ISMAI, S. Pedro Avioso, Portugal, ^8^Childhood and Adolescence Department / Pediatric Service of Oporto Hospital Centre, Porto, Portugal, ^9^Children and Adolescent Centre, CUF Hospital, Center for Health Technology and Services Research (CINTESIS), Faculty of Medicine, University of Porto, Porto, Portugal (e-mail: ssn.coimbra@gmail.com)

Epidemiological data confirms that obesity associates with iron deficiency in children and adolescents. Several mechanisms have been proposed to explain this association. One of these explanations suggest that this state of hypoferremia is a consequence of obesity-related inflammation that increments the production of the acute-phase protein hepcidin, the major regulator of iron availability. Iron supplementation has been proven to be of little effectiveness in obese children, not being, apparently, a good approach to correct hypoferremia. We aim to clarify the relationship between inflammation, hepcidin and iron status in obese children and adolescents. Thirty-four obese children and adolescents were involved in a physical exercise (PE) program over a period of 8 months. Besides regular PE classes at school 3 times a week, participants were enrolled in an extra-activity PE program twice a week, resulting in a total of 5 hours per week of moderate to vigorous PE. Anthropometric characterization and clinical evaluation was performed before and after the program intervention. Biochemical analysis included determination of hepcidin, interleukin (IL)-6, iron and soluble transferrin receptor (sTfR), a biomarker that reflects the functional iron compartment, increasing when the iron functional pool is depleted. At baseline, IL-6 correlated significantly and positively with hepcidin (r = 0.491; *P* = 0.004). At the end of the study, it was observed a significant decrease in the values of body mass index (BMI; *P* = 0.034) and BMI z-score (*P* < 0.001). These anthropometric alterations were accompanied by a significant decrease in the values of IL-6 (*P* = 0.039), hepcidin (*P* = 0.039) and sTfR (*P* = 0.027), as well as a significant increase in iron levels (*P* = 0.006). The decline in inflammation, manifested in a decrease of IL-6 levels, was associated with a reduction of hepcidin and an improvement in iron status. Indeed, sTfR, considered the more reliable and sensitive marker of iron status, decreased and this change was accompanied by an increase in iron levels. Our data confirms the association between inflammation and iron status in obesity. Moreover, the reduction of the inflammatory process, accomplished by BMI decrease, seems to be a good approach to correct hypoferremia in obese children and adolescents.

**Acknowledgements:** This work was funded by European Regional Development Fund (FEDER, Europe) funds through the Operational Competitiveness Programme (COMPETE, Portugal) and by National Funds through Fundação para a Ciência e a Tecnologia (FCT, Portugal) under the project FCOMP-01-0124-FEDER-028613 (PTDC/DTP-DES/0393/2012).

### P26 – Determination of macrophage activation profile during *Toxoplasma gondii* infection and implications on pregnancy outcome using the mice model

C Brito, BSc^1,2^, A Gonçalves, MD^1^, TM Silva, PhD^3^, N Teixeira, PhD^4^, M Borges, PhD^4^. ^1^Department of Biological Sciences, Faculty of Pharmacy, University of Porto, Portugal, ^2^Department of Biology, University of Aveiro, Portugal, ^3^Institute for Molecular and Cell Biology, University of Porto, Portugal, ^4^UCIBIO/REQUIMTE, Department of Biological Sciences, Faculty of Pharmacy, University of Porto, Portugal (e-mail: mborges@ff.up.pt)

*Toxoplasma gondii* is one of the most widespread opportunistic protozoan parasites and *T. gondii* infections are generally asymptomatic in animals or immunocompetent humans. However, a primary infection occurring during human pregnancy, particularly during the 1^st^ trimester, can cause unfavourable pregnancy outcomes such as miscarriage, stillbirth, or fetal abnormalities. The mechanisms playing a role in the induction of pathology during infection are not clear but are potentially associated with disruption of normal homeostatic immunological mechanisms including macrophage activation. Human and animal studies support that aspects of both classical and alternative activation occur in macrophages responding to *T. gondii*. Thus, while classical macrophage activation can control replication through induction of inducible (type 2) nitric oxide synthase (iNOS), alternative activation can control parasite replication through induction of arginase 1 (Arg-1) and depletion of arginine. Our study focused on the effects of *T. gondii* infection on Arg-1 and iNOS expression at fetomaternal interface and at systemic level (peritoneal exudate cells and spleen cells) using the mice model. Infection of pregnant BALB/c and C57Bl/6 mice with a type II strain of *T. gondii* allowed the follow-up of pregnancy. Morphometric analysis of decidua and placenta was performed using hematoxilin-eosin stained sections of the fetoplacental units. The evaluation of parasite loads in the organs was done by quantitative Real Time-PCR using Taqman probes. The presence and the activation profile of macrophages at the fetomaternal interface were determined by the evaluation of Arg-1 and iNOS expression by RT-PCR, immunohistochemistry and Western Blotting. It was observed a significant decrease in the placental and decidual areas of infected B6 compared to control, but not of Balb/c mice. Immunohistochemical analysis indicated an increased expression of Arg-1 and a decreased expression of iNOS in the decidua from infected compared to control animals from both strains of mice. This indicates that infection interferes with the process of placentation and delay decidualization in B6 mice, which is correlated with altered expression of Arg-1 and iNOS. This work not only aims at understanding the role of macrophage activation in congenital infection by *T. gondii,* but should provide valuable information regarding the role of macrophages during healthy pregnancy and pregnancy complicated by disease.

## NEPHROLOGY

### CO28 – Prospective evaluation of plasmatic advanced glycation endproducts (age) after pancreas-kidney transplantation: – carboxymethyllisine significantly decreased during the first year

LaSalete Martins, MD, PhD^1,3^, José C Oliveira, MD^2^, Isabel Fonseca, PhD^1,3^, António C Henriques, MD^1,3^, Anabela Rodrigues, MD,PhD^1,3^. ^1^Nephrology and ^2^Clinical Pathology Departments, Hospital Santo António – CHP, Porto, Portugal, ^3^Unit for Multidisciplinary Research in Biomedicine - Institute of Biomedical Sciences Abel Salazar and University Hospital de Santo António, University of Porto, Porto, Portugal (e-mail: lasalet@gmail.com)

Diabetes mellitus (DM) leads to increased Advanced-Glycation Endproducts (AGE) formation, which has proved to be associated with micro and macrovasular diabetic complications. Type-1 DM patients undergoing simultaneous pancreas-kidney transplantation (SPKT) can restore normoglycemia and renal function, eventually decreasing AGE accumulation. There is a lack of knowledge in this field. To prospectively study the evolution of plasmatic AGES after SPKT. Circulating AGE were assessed in 20 patients, through blood samples collection, at time 0 (day of transplantation - T0), 3 (T3), 6 (T6) and 12 (T12) months after successful SPKT. Global AGE and carboxymethyllisine (CML) were analyzed using specific ELISA kits. We also analyzed advanced oxidation protein products (AOPP), using a specific colorimetric kit. All patients maintained both grafts with normal function. Global AGE mean values were 16.83 ± 6.39 μg/mL at T0; 17.14 ± 3.76 μg/mL at T3; 17.46 ± 5.64 μg/mL at T6; and 15.99 ± 5.17 μgl/mL at T12. CML mean values were 0.94 ± 0.36ng/mL at T0; 1.11 ± 0.48ng/mL at T3; 0.99 ± 0.42ng/mL at T6; and 0.78±0.38ng/mL at T12. AOPP mean values were 130.09 ± 76.83 μMol/L at T0; 137.25 ± 110.60 μMol/L at T3; 116.39 ± 51.20 μMol/L at T6; and 106.40 ± 57.93 μMol/L at T12. CML variation was statistically significant (*P* = 0.022); AOPP variation nearly significant (*P* = 0.076). Several potential interfering factors with the dynamic process of AGE formation were also analyzed. With the exception of renal function and euglycemia restoration, we could not find factors (diabetes time, glycated hemoglobin, age, hypertension, lipid profile) significantly associated with the variation of these markers. Time on dialysis was the single factor with a nearly significant positive correlation with CML values (*P* = 0.071). CML decrease could be observed during the first year, meaning that the glycoxidation process can possibly be halted or even reverted in these patients with both grafts functioning; and that this process may start early after SPKT. Studies in larger samples with prolonged follow-up are needed to confirm these data.

### CO29 – Kidney transthyretin amyloidosis after domino liver transplantation: second report

Ana Rocha, MD^1,2^, Josefina Santos, MD^2,3^, Ramón Vizcaíno MD^4^, Helena Pessegueiro MD^5^, Cristina Alves MD^1^, Luísa Lobato, PhD^1,2,3^. ^1^Unidade Corino de Andrade, Centro Hospitalar do Porto – CHP, Porto, Portugal, ^2^Unit for Multidisciplinary Research in Biomedicine-UMIB, Instituto de Ciências Biomédicas Abel Salazar-ICBAS, University of Porto, Porto, Portugal, ^3^Department of Nephrology, CHP, Porto, Portugal, ^4^Department of Pathology, CHP, Porto, Portugal, ^5^Liver Transplant Unit, CHP, Porto, Portugal (e-mail: acrisbraga@gmail.com)

Hereditary transthyretin (ATTR) amyloidosis is an autosomal-dominant disease caused by a point mutation in the *TTR* gene. The phenotype of the most common mutation, V30 M, is a progressive sensorimotor and autonomic neuropathy accompanied by the involvement of the heart, eye, gastrointestinal tract and kidney. The most effective treatment for ATTR amyloidosis is liver transplantation, where the source organ of variant TTR production is exchanged by another producing wild type only. To ameliorate organ shortage, domino liver transplantation (DLT) has been proposed, using the explanted variant TTR producing liver for transplantation of another patient. As neuropathy usually begins in the thirties, it was expected that recipients would die before symptomatic amyloidosis. However, DLT recipients can develop symptoms 7–8 years after surgery and amyloid have been found in tissues 3 years after. We present a case of *de novo* amyloidosis in a 65-year-old man, who underwent DLT 10 years earlier, confirmed by salivary gland, kidney and explanted liver biopsy. He had viral hepatitis C cirrhosis with hepatocellular carcinoma. The recipient started typical neuropathy 9 years after DLT with rapid deterioration and required a pacemaker implantation. In the presence of progressive renal dysfunction (eGFR by MDRD 25 mL/min) without proteinuria and considering the possibility of liver retransplantation, a renal biopsy was performed. Congo red staining and immunohistochemistry of kidney specimen showed interstitial, arteriolar, peritubular and tubular basement membrane ATTR amyloid deposits; ATTR was scarce in glomeruli. Amyloid deposition in salivary gland was also demonstrated. The subsequent option was isolated liver retransplantation using a deceased donor. Pathologic examination of the explanted liver revealed a thick layer of amyloid within the walls of the portal vessels. Five years later, the patient ameliorated his neurologic status; eGFR recovered to 35 mL/min and maintains absence of albuminuria, without signs or symptoms of other organ system involvement. This is the second description of acquired ATTR kidney amyloidosis. On contrary to the other report, the decision was isolate liver retransplantation and not a liver-kidney procedure. Renal histological analysis contributed for deciding which organs should be transplanted. Systematic follow-up of DLT recipients should include, besides a sequential neurological evaluation, a careful assessment of kidney function.

### CO30 – Admissions to a nephrology ward in patients undergoing chronic dialysis: impact of diabetes mellitus

Vanda Guardado, MD^1^, Lígia Bessa, MD^1^, Carla Leal Moreira, MSc^2^, Jorge Malheiro, MD^2^, Josefina Santos, MD^2^, António Cabrita, MD^2^. ^1^Hospital Militar de Luanda, ^2^Hospital Geral de Santo Antonio –Centro Hospitalar do Porto CHP (e-mail: vandateixeira04@hotmail.com)

Diabetes mellitus (DM) is the leading cause of end-stage chronic kidney disease (ESRD) worldwide, and both conditions are associated with complications demanding in-hospital admission. We aimed to study the impact of DM on the characteristics and outcomes of chronic dialysis patients’ admissions. We analyzed admissions to our unit in the 1st semester of 2012 (n = 144). We selected for this analysis those patients undergoing chronic dialysis (n = 55). A comparative analysis between patients with (n = 19) and without (n = 36) DM was performed. No significant difference was observed, between patients without vs with DM, for age (median 62 vs 67 years, P = 0.117), gender (male:female, 20:16 vs 11:8, P = 0.868) and body mass index (22.1 vs 23.5 Kg/m2; P = 0.178), respectively. Patients with DM presented a higher prevalence of heart failure (79% vs 42%; P = 0.014), coronary (42% vs 19%; P = 0.073), cerebrovascular (58% vs 17%; P = 0.002) and peripheral arterial (53% vs 25%; P = 0.040) disease in comparison with those without DM. The use of platelet antiaggregation agents was more common in diabetic patients (74% vs 36%; P = 0.005). Dialysis modality was similar between groups (hemodialysis:peritoneal dialysis, DM 16:3 vs no DM 26:10 patients; P = 0.506). Infectious complications were the main cause (53%) of admission, particularly in diabetic patients (DM 68% vs no DM 44%; P = 0.090). Infections were mainly respiratory (DM 62% vs no DM 53%), followed by soft tissues/articular (DM 46% vs no DM 18%) and dialysis access-related (DM 23% vs no DM 25%). No difference in overhydration-related admissions was observed (DM 16% vs no DM 14%; P = 1.0). Frailty Charlson score was significantly higher in diabetic (median 9; P25-P75 8-12) in comparison with non-diabetic (median 7; P25-P75 5-9) patients (P = 0.002). Three (8%) non-diabetic and 2 (11%) diabetic patients died while admitted (P = 1.0). Readmission at 30 days post discharge was identical between diabetic (21%) and non-diabetic (28%) patients (P = 0.749). One-year hospitalization rate after index admission was 8.65 episodes in diabetic and 7.28 episodes per 100 patients-month in non-diabetic patients (P = 0.557). Diabetes in patients undergoing chronic dialysis seems to be associated with a higher prevalence of vascular comorbidities and frailty score. Infections-related admissions are also more common in these patients. Still, no significant impact on mortality and rehospitalization rates was observed.

### CO31 – The impact of Diabetes Mellitus in hospitalized patients with non-end stage renal disease or acute kidney injury

Carla Moreira, MSc^1^, Lígia Bessa, MD^2^, Vanda Guardado, MD^2^, Jorge Malheiro, MD^1^, Josefina Santos, MD^1^, António Cabrita, MD^1^. ^1^Hospital Geral de Santo António, Centro Hospitalar do Porto, ^2^Hospital Militar de Luanda (e-mail: moreira.l.s.carla@gmail.com)

Diabetes Mellitus (DM) is a major cardiovascular risk factor; in the US, 35% of adults who have diabetes also have chronic kidney disease (CKD). When associated with kidney disease, diabetes can lead to end stage renal disease, contribute to the development of acute kidney injury (AKI) and increase the risk for hospitalization. With this study we intend to describe the contribution of DM in patient's hospitalization characteristics and as a predictor for rehospitalization. We conducted a retrospective study at our nephrology ward. A univariate analysis was performed using demographic features and laboratory data from 89 randomly selected non-end stage renal disease patients hospitalyzed in our ward from 2012 to 2013. Based on the univariate analysis results we conducted a multiple logistic regression analysis and poisson regression. A total of 89 patients were included in this study, 67.4% (n = 60) of males, with a median age of 68yo (IQ 55-81). The prevalence of diabetes in this group of patients was 40.4% (n = 36). The median HbA1c level was 6.40% (IQ 5.6-7.0). Diabetic patients were significantly older (73yo vs 62yo, p = 0.004), with higher body mass index (28.0 vs 24.7 Kg/m2, p = 0.017) and were associated with more urgent admissions (88.9% vs 71.7%, p = 0.052), higher prevalence of heart failure (44.4% vs 20.8%, p = 0.017), ischemic cardiomyopathy (30.6% vs 13.2%, p = 0.046) and periphery arterial disease (33.3% vs 13.2%, p = 0.034) than non-diabetic patients. Diabetic patients were also more likely to be admitted for cardiac related causes (13.9% vs 1.9%, p = 0.038). We found no statistical significant association between DM and CKD, AKI, infection or site of infection, but DM was associated with higher number of dysfunctions in sepsis (1.86 vs 0.75 in media, p = 0.001). Diabetic patients were significantly associated with higher 30-day rate of readmission (OR 3.45, p = 0.03), higher rate of rehospitalization at three-months (7.44 vs 22.55 admissions per 100 patient-month, p = 0.003), at six-months (5.27 vs 16.44 admissions per 100 patient-month, p < 0.001) and at one-year (4.65 vs 10.57 admissions per 100 patient-month, p = 0.001). When adjusting for the main cause of admission, DM maintains a trend for significance in association with readmission (OR 3.18, p = 0.057). DM is an important predictor for 30-day readmission and rehospitalization until one year.

### CO32 – Pregnancy in renal transplant recipients: obstetric outcomes and risk of allosensitization

Sofia Santos, MD^1^, Jorge Malheiro, MD^1,2^, Andreia Campos, MD^1^, Sofia Pedroso, MD^1^, Manuela Almeida, MD^1^, La Salete Martins, PhD^1,2^, Leonídio Dias, MD^1^, Castro Henriques, MD^1^, António Cabrita, MD^1^. ^1^Nephrology Department, Centro Hospitalar do Porto - Hospital Santo António, Porto, Portugal, ^2^Unit for Multidisciplinary Research in Biomedicine – ICBAS, Porto, Portugal (e-mail: sofia.fersantos@gmail.com)

Kidney transplantation improves women's chances of becoming pregnant and of having a live birth. Donor specific anti-HLA antibodies are responsible for antibody-mediated rejection and reduced kidney allograft survival. After pregnancy, woman may develop anti-HLA antibodies after having encountered HLA alloantigens of the fetus inherited from the father. The aim was to describe the outcomes of pregnancies and analyze its impact on *de novo* allosensitization. A retrospective study of all pregnancies occurring in our center since 2010 to 2014, in women with a functioning graft was made; miscarriages during the first trimester were excluded. We assessed maternal and fetal complications: late miscarriage (2nd and 3th trimesters), preeclampsia, fetal malformations, preterm birth, and low birth weight (<2.5Kg). Anti-HLA antibodies were screened before and after pregnancy (solid-phase assay). After pregnancy we examined the evolution and episodes of acute rejection. Within that period, 11 pregnancies occurred in 10 women. We excluded 3 because of missing data. Overall, the live birth rate was 88% (7/8 pregnancies). One pregnancy was interrupted during second trimester because of severe fetal malformation (heart). Preeclampsia occurred in 2 patients and a threat of premature delivery occurred in one case. 3 newborns were premature (<37 weeks) and 2 had low birth weight (<2.5Kg). Delivery was done by caesarean in 6 of the 7 live births. In all cases GFR remained relatively stable during and after delivery. Before pregnancy, no anti-HLA antibodies were detected in any women. After pregnancy, *de novo* allosensitization was detected in 2 of them: they developed *de novo* anti-HLA antibodies, at 9 and 20 months post-delivery. In the 2nd patient, the development of *de novo* anti-HLA antibodies did not have impact on the graft. In the 1st patient, the biopsy performed showed acute antibody-mediated rejection and also acute T-cell-mediated rejection with mild to moderate intimal arteritis (Banff IIA). In spite of the treatment she progressed with renal function decline, having lost her graft function and entering dialysis after 11 months post-delivery. In conclusion, despite complications, the outcomes for pregnancy and kidney allografts are good. In our cohort 1(/8) case of *de novo* allosensitization after pregnancy leading to antibody mediated rejection and consequent graft loss was observed. Careful screening of anti-HLA antibodies emergence after pregnancy in kidney transplant patients is advisable.

### CO33 – Risk Factors for Mortality in End-Stage Kidney Disease Patients Under Online-Hemodiafiltration: Three-Year Follow-Up Study

Pedro Sousa-Martins, MD^1^, Alexandra Moura, PhD^2^, José Madureira, MD^3^, Pablo Alija, MD^3^, José Gerardo Oliveira, MD^4^, Martin Lopez, MD^5^, Madalena Filgueiras, RN^6^, Leonilde Amado, RN^7^, Maria Sameiro-Faria, MD^7^, Vasco Miranda, MD^7^, Edgar Mesquita, MSc^8^, Laetitia Teixeira, PhD^1^, Alice Santos-Silva, PhD^9,10^, Luísa Lobato, MD, PhD^11,12^, Elísio Costa, PhD^9,10^. ^1^Instituto de Ciências Biomédicas Abel Salazar, Universidade do Porto, ^2^Instituto de Ciências da Saúde, Universidade Católica Portuguesa, Porto, Portugal, ^3^Clínica de Hemodiálise NefroServe, Barcelos, Portugal, ^4^Centro Hospitalar do Porto, Porto, Portugal, ^5^Clínica de Hemodiálise de Felgueira, Felgueiras, Portugal, ^6^Clínica de Hemodiálise de Gondomar, Gondomar, Portugal, ^7^Clínica de Hemodiálise NephroCare, Maia, Portugal, ^8^Núcleo de Estudantes de Estatística, Universidade do Minho, Braga, Portugal, ^9^Laboratório de Bioquímica, Departamento de Ciências Biológicas, Faculdade de Farmácia, Universidade do Porto, Portugal, ^10^UCIBIO, REQUIMTE, Universidade do Porto, Portugal, ^11^Instituto de Ciências Biomédicas Abel Salazar, Universidade do Porto,^12^Serviço de Nefrologia & Departamento de Ensino, Formação e Investigação, Centro Hospitalar do Porto, Portugal (e-mail: emcosta@ff.up.pt)

End-stage kidney disease (ESRD) patients under dialysis have high mortality rate. Inflammation, poor nutritional status and disturbances in erythropoiesis and iron metabolism have been reported in these patients. Moreover, there is a growing concern about the health related quality of life (HRQOL) in the context of ESRD. The aim of this work was to study the predictive value of these disturbances, dialysis adequacy and of HRQOL for mortality risk, by performing a three-year follow-up study. Clinical, socio-demographical and analytical data (dialysis adequacy, nutritional status, hematological data, lipid profile, iron metabolism and inflammatory markers) were obtained from 236 patients (61.02% male; 67.50 [56.00-75.00] years old) under online-hemodiafiltration. Patient's reported HRQOL score was assessed by using the Kidney Disease Quality of Life-Short Form (KDQOL-SF). 54 patients died during the 3 years follow-up period. Our data showed that mean cell hemoglobin concentration (MCHC), transferrin and albumin are significant predictors of mortality. The risk of death was higher in patients presenting lower levels of MCHC (Hazard ratio [HR] = 0.70; 95% confidence interval [CI] = 0.500-0.984), transferrin (HR = 0.99; 95% CI = 0.982 – 0.998), and albumin (HR = 0.96; 95% CI = 0.938-0.994). Our study showed that poor nutritional status and an inflammatory-induced iron depleted erythropoiesis are important factors for mortality in these patients. MCHC, transferrin and albumin may provide useful biomarkers of risk in ESRD patients under OL-HDF.

### CO34 – Impact of de novo donor-specific anti-hla antibodies in kidney graft failure: a case-control analysis

Jorge Malheiro, MD^1,3^, Sandra Tafulo, MD^2,3^, Leonidio Dias, MD^1^, La Salete Martins, MD, PhD^1,3^, Idalina Beirão, MD, PhD^1,3^, António Castro-Henriques, MD^1,3^. ^1^Nephrology & Kidney Transplantation Unit – Hospital de Santo António, Centro Hospotalar do Porto (HAS-CHP), ^2^CSTP - IPST, ^3^Unit for Multidisciplinary Research in Biomedicine (UID/Multi/00215/2013) UMIB – ICBAS (e-mail: jjorgemalheiro@gmail.com)

Data on the prevalence and impact of *de novo* donor-specific anti-HLA antibodies (dnDSA) in the process leading to kidney graft (KG) failure are still scarce. We investigated 56 patients transplanted between 2000-2010 with KG failure (cases) and 56 patients with a functioning graft (controls) matched for: donor type, transplant number and year, recipient age/gender, donor age/gender, HLA mismatches and dialysis vintage. All patients had at least one serum collected 1 year before failure or end of follow-up (Dec2014), in which dnDSA detection was performed. Data on the KG pathology was collected considering the last available sample. Mean KG survival years were 9.6 and 4.7 for controls and cases respectively. dnDSA was present in 54% of cases versus 16% of controls (P < 0.001). dnDSA against class I, II and I+II was detected in 27%, 13% and 14% of cases, and 5%, 9% and 2% of controls, respectively (P < 0.001). Delayed graft function was more common in cases (48%) than in controls (21%) (P = 0.003), as was acute rejection (21% in cases, 9% in controls; P = 0.065). At 6-years, 75.5%, 27.8%, 53.8% and 37.5% of grafts survived in patients with no DSA, dnDSA I only, dnDSA II only and dnDSA I+II, respectively (P < 0.001). Cox regression showed that delayed graft function (HR 1.927, P = 0.019) and dnDSA (HR 2.656, P = 0.001) were independent predictors of graft failure, adjusted for confounding factors (acute rejection and type of induction). Forty-two cases and 11 of controls had at least one indication biopsy. Transplant glomerulopathy was present in 18 cases and 4 controls (P = 0.001). Acute antibody mediated rejection was present in 8 cases and 1 control (P = 0.032). Considering only 36 biopsies performed at the same time of dnDSA detection, 22 were done in dnDSA+ patients and 14 in DSA- patients. Transplant glomerulopathy was present in 14 DSA+ and 3 DSA- patients (P = 0.013). Acute antibody mediated rejection was present in 5 DSA+. Graft function in controls at last visit was significantly lower in dnDSA+ (34 ml/min) than in dnDSA- (52 ml/min) patients (P = 0.041). Proteinuria > 0.5 g/g occurrence was similar between groups (dnDSA- 28%, dnDSA+ 39%, P = 0.504). dnDSA presence was associated with antibody-mediated graft injury observed in our histological data. dnDSA was also associated with worse graft function in control patients. Posttransplant emergence of dnDSA was clearly associated with graft loss, independently from HLA class.

## NEUROCIENCES AND AUTOIMMUNITY

### CO35 – Microangiopathy in Systemic Sclerosis: role of endothelial dysfunction and microvascular damage

I Silva, MD^1^, A Teixeira, PhD^2^, J Oliveira, MD^3^, R Almeida MD^4^, C Vasconcelos MD, PhD^5^. ^1^Angiology and Vascular Surgery Service and Clinical Immunology Unit, Centro Hospitalar do Porto, Portugal, ^2^CINTESIS - Center for Research in Health Technologies and Information Systems Porto, Portugal, ^3^Clinical Pathology Department, Centro Hospitalar do Porto, Portugal, ^4^Angiology and Vascular Surgery Service, Abel Salazar Biomedical Health Institute, Porto, Portugal, ^5^Clinical Immunology Unit, Multidisciplinary Unit for Biomedical Investigation (UMIB), University of Porto, Porto, Portugal (e-mail: heitor.ivone@gmail.com)

Raynaud's phenomenon (RP) and digital ulcers (DU) are frequent among systemic sclerosis (SSc) patients. Up to 30−50 % of SSc patients will experience one or more DU throughout the course of their disease. Our aim was to investigate endothelial dysfunction and microvascular abnormalities in nailfold videocapillaroscopy (NVC) and explore their diagnostic and predictive value for digital ulcers (DU) in patients with RP. Endothelial dependent flow-mediated dilatation (FMD) and serum levels of Endothelin-1 (ET-1) and ADMA were analysed in a cohort study of 77 SSc patients. Patients were divided into two groups: (i) naïve DU patients (39) and (ii) active DU at baseline (38 patients) and followed for 3 years. Main outcome was a new DU event. Late NVC late scleroderma pattern (AUC: 0.846 95%CI: 0.760-0.932), lower values of FMD (AUC: 0.754 95%CI: 0.643-0.864), increased serum levels of ET-1 (AUC: 0.758 95%CI: 0.649-0.866), ADMA (AUC: 0.634 95%CI: 0.511-0.757) and endoglin were significantly associated to new DU events in the 3-year follow-up. Additionally, telangiectasia (*p < 0.001*), sclerodactily (*p < 0.001*), diffuse disease subset (*p = 0.001*) and positive Allen test (*p < 0.001*) were significantly more frequent in patients with active DU at enrolment. Univariate Cox regression analysis showed that FMD > 9.41% (HR: 0.37 95%CI: 0.14-0.99); ET-1 >11.85 *pmol/L* (HR: 3.81 95%CI: 1.41-10.26) and late NVC pattern (HR: 2.29 95%CI 0.97-5.38) were predictors of DU recurrence during follow-up. Multivariate Cox regression confirmed all except FMD as independent predictors. When estimating the probability of occurrence of first DU in naïve DU patients, in univariate analysis, only late NVC pattern (HR: 12.66 95%CI: 2.06-77.89) was an independent predictor confirmed by multivariate Cox regression. Endothelial dysfunction assessed by FMD, ET-1 and severe microangiopathy damage, as evidenced in late NVC patterns, were found to be independent predictors of DU recurrence in a 3-year follow-up. Nailfold capillaroscopic findings are the best available and reproducible tool to identify SSc patients at risk for DU development.

Acknowledgments: This research was supported by a PhD research grant from Teaching and Research Department (Departamento de Ensino e Investigação) of Oporto Hospital Center (reference 054/10 (036-DEFI/051-CES) and FCT postdoc grant SFRH/BPD/86383/2012.

### CO36 – Transthyretin promotes amyloid beta peptide clearance through the low-density lipoprotein receptor-related protein 1 in the liver

Mobina Alemi, MSc^1,2,3^, Cristiana Gaiteiro, MSc^1,3^, Carlos Alexandre Ribeiro, PhD^1,3^, Sandra Marisa Oliveira, PhD^1,2,3^, Maria João Saraiva, PhD^1,3^, Isabel Cardoso, PhD^1,3^. ^1^IBMC – Instituto de Biologia Molecular e Celular, 4150-180 Porto, Portugal, ^2^FMUP – Faculdade de Medicina, Universidade do Porto, 4200-319 Porto, Portugal, ^3^I3S – Instituto de Investigação e Inovação em Saúde, Universidade do Porto, Portugal (e-mail: mobina.alemi@ibmc.up.pt)

Transthyretin (TTR) is a protective molecule in Alzheimer's disease (AD) and is decreased in cerebrospinal fluid (CSF) and plasma of AD patients. AD transgenic mice with only one copy of the TTR gene show increased amyloid beta peptide (Aβ) brain and plasma levels when compared to animals with two copies of the gene, leading to the hypothesis that TTR is involved in Aβ brain efflux and peripheral clearance. The rapid peripheral clearance of Aβ is mediated mainly by hepatic low-density lipoprotein receptor-related protein 1 (LRP1), which can be blocked by RAP. TTR, mainly synthesized by the liver, also suffers hepatic degradation and its receptor has been characterized as a RAP-sensitive receptor. Thus it is possible that TTR participates in Aβ clearance through hepatic LRP1. We evaluated plasma Aβ_1-42_ levels in young AD/TTR mice with different genotypes for TTR by ELISA, and showed that the lack of TTR results in early plasma Aβ_1-42_ impairment, as 3 months-old AD/TTR-/- mice presented higher plasma Aβ_1-42_ levels than AD/TTR+/+ animals, further supporting a role for TTR in Aβ peripheral elimination. To assess TTR effect in Aβ uptake by the liver we evaluated FAM labeled Aβ_1-42_ internalization by flow cytometry in SAHep cells (a hepatoma cell line that does not express TTR) incubated with or without human recombinant TTR and in primary hepatocytes from AD and from non-transgenic mice, with different genotypes for TTR. Our results showed that the presence of TTR promoted Aβ_1-42_ internalization, both in SAHep cells and in primary hepatocytes. Finally, and to investigate possible differences in the expression of the LRP1 gene, we performed real time RT-PCR (qRT-PCR) and immunocytochemistry or western blot, in SAHep cells incubated with or without TTR and also in the livers of mice with different TTR backgrounds. As our results indicated, LRP1 levels, both mRNA and protein, were reduced in SAHep cells grown in the absence of TTR and in TTR-/- livers, as compared to SAHep cultivated in the presence of TTR and to TTR+/+ livers, respectively. In conclusion, our results indicate that TTR promotes Aβ_1-42_ uptake by hepatocytes, probably through LRP1, therefore preventing Aβ accumulation in blood, which in turn will preclude Aβ brain influx and its consequent accumulation in the brain, as it happens in AD.

**Acknowledgements:** We thank Maria Coelho for her help with the primary hepatocytes and Diana Martins and Paula Magalhães for their support in qRT-PCR. This work was funded by national funds through FCT and FEDER within the partnership agreement PT2020 related with the research unit number 4293, and by projects FCOMP-01-0124-FEDER-037277, PTDC/QUI-BIQ/118076/2010, PTDC/SAU-ORG/116645/20109 and by Fundació La Marató, Spain (20140330-31-32-33-34). Isabel Cardoso works under the Investigator FCT Program HPOP type 4.2, Mobina Alemi is recipient of a Master Fellowship (BIM) from IBMC funded by project of Fundació La Marató, Spain. Carlos Alexandre Ribeiro was a recipient of the fellowship PEST-LA1101401-BPD.

### CO37 – Calculating pre-deliric model with saps ii for early detection of delirium in icu patients.

Marco Sampaio, MD^1^, Teresa Cardoso, MD, PhD^2^, Irene Aragão, MD^2^. ^1^PhD student at Centro Hospitalar do Porto, ^2^Hospital de Santo António - Centro Hospitalar do Porto (HSA-CHP), Unidade de Cuidados Intensivos Polivalente (e-mail: marcosampaiopinto@gmail.com)

Delirium is a common and serious disorder, prevalent in Intensive Care Units (ICU) patients, associated with negative clinical outcomes, namely higher morbidity and mortality. Accurate diagnosis is limited. General preventive measures in all ICU patients are time and resources consuming without consensual results. Predicting delirium enables physicians to focus on high-risk patients with non-pharmacological preventive measures and drug interventions resulting in better outcomes. The risk of delirium can be assessed through PRE-DELIRIC, but it uses APACHE II; there are no studies using SAPS II, although most European ICUs use SAPS II instead of APACHE II as physiological severity score to estimate death risk. The purpose of this study was to validate PRE-DELIRIC model using SAPS II score in sedated patients. Prospective cohort study performed between September 2012 and February 2013 in all adult patients, admitted for a minimum period of 48 hours and sedated for at least 24 hours. Setting: Twelve-bed mixed ICU at an University-affiliated, tertiary care Hospital. Main outcome measure: To compare PRE-DELIRIC scores calculated with APACHE II and SAPS II. One-hundred patients were included in the study. The APACHE II score calculated through a mathematical model from SAPS II was compared with the SAPS II for each patient and presented an Intra-class Correlation Coefficient (ICC) of 0.93. PRE-DELIRIC calculated with APACHE II was compared with the one calculated with SAPS II and presented an ICC of 0.99. It is possible to use SAPS II in the calculi of risk of delirium through PRE-DELIRIC score, allowing identification of high-risk patients and early institution of preventive measures.

### CO38 – Animal model of cuprizone-induced demyelination: the beginning of a story with possible clinical applications

Filipe Palavra, MD^1,2,3^, Sara Nunes, MSc^1,2^, Lorena Petrella, PhD^3^, João Dinis^1,2^, Frederico C Pereira, PhD^1,2^, Luís Almeida, MD, PhD^1,2^, António F. Ambrósio, PhD^1,2^, Miguel Castelo-Branco, MD, PhD^2,3^, Flávio Reis, PhD^1,2^. ^1^Laboratory of Pharmacology and Experimental Therapeutics, Institute for Biomedical Imaging and Life Sciences (IBILI), Faculty of Medicine, University of Coimbra; Coimbra; Portugal, ^2^CNC.IBILI, University of Coimbra; Coimbra; Portugal, ^3^Institute of Nuclear Sciences Applied to Health, University of Coimbra; Coimbra; Portugal (e-mail: fpalavra@fmed.uc.pt)

Cuprizone is a copper chelator that produces a reversible oligodendrocytopathy in animals, similar to human multiple sclerosis. This model is attractive to study remyelination, but some of its fundamental properties are not yet fully characterized. The objective was to characterize, in behavioural and imaging terms, the animal model of cuprizone-induced demyelination. A 0.2% solution of cuprizone was orally administered to 16 C57BL/6 mice during 5 weeks. After this time (W5), 8 animals were evaluated using Rotarod test (15–25 rpm, 100 s each) and 6 of them were submitted to Magnetic Resonance Imaging (BioSpec94/20 USR, 9.4T), using T2-weighted sequences and Diffusion Tensor Imaging (DTI). The protocol was repeated using the remaining animals, 2 weeks later (W7). At W5, animals exposed to cuprizone had a significant reduction in time spent on Rotarod device, versus controls (138.3 s *vs*. 164.8 s, p = 0.03). At W7, no significant differences were found, between groups (147.9 s *vs.* 162.7 s, p = 0.44). Comparing image data at W5 and W7, no differences were found in terms of total brain volume (p = 0.56) and of corpus callosum (CC) volume (p = 0.16), but a significant increase in cortical volume was detected (p = 0.03). From W5 to W7, there was an increase in T2-wheigted signal intensity of CC, after normalization with the thalamus (p = 0.03), and a correlation between volume and intensity of CC at W7 (p = 0.001). In an analysis performed by voxel-based morphometry and DTI, a trend towards statistical significance was observed considering the differences measured in CC, anterior commissure and cerebral cortex, between W5 and W7. Although preliminary, these data are consistent with differences in various relevant parameters recorded at the peak of demyelination (W5) and at an early stage of spontaneous remyelination (W7), in this animal model. The study will continue with a biochemical, molecular and immunohistochemical analysis, drawn with pharmacotherapeutic intention.

**Acknowledgements:** Portuguese Foundation for Science and Technology (Strategic Projects Pest C/SAU/UI3282/2013 and UID/NEU/04539/2013), COMPETE-FEDER and Biogen.

### CO39 – “Mind the method!” – A longitudinal assessment of methodological soundness and quality of reporting of als research on the sod1g93a mouse

Franco, NH, PhD^1,2^, JG Fernandes, MSc^1,2^, AJ Grierson, PhD^3^, AJ Furley, PhD^4^, IAS Olsson, PhD^1,2^. ^1^i3S - Instituto de Investigação e Inovação em Saúde, Universidade do Porto, Portugal, ^2^IBMC - Instituto de Biologia Molecular e Celular, Universidade do Porto, ^3^The University of Sheffield; SITraN, Sheffield, UK, ^4^The University of Sheffield, Department of Biomedical Science, Sheffield UK (e-mail: nfranco@ibmc.up.pt)

Persistent translational failure of candidate drugs for Amyotrophic Lateral Sclerosis (ALS) has raised criticism over the predictive value of current animal models. However, it has been argued that clinical trials are being based on false-positive results from poorly designed, underpowered animal studies (e.g. Perrin,2014. Nature 507:423-425; Scott et al, 2008. Amyotroph Lat Scl 9(1):4-15). The ALS research community responded by issuing guidelines, in 2007 (Ludolph et al. Amyotroph Lat Scl 8(4):217-223) and 2010 (Ludolph et al. Amyotroph Lat Scl 11(1-2): 38-45), for carrying out and reporting animal studies. We present results from a systematic review of scientific and reporting standards of ALS studies published both before and after publication of said guidelines. A sample of 382 studies – published in 2005, 2009, 2011 and 2013 – on the SOD1 mouse model of ALS was retrieved. Studies were classified as either “preclinical” (n = 72) or “proof-of-concept” (n = 310), and reporting of relevant research parameters and experimental design was assessed. Preclinical studies reported significantly more details on central variables than proof-of-concept studies, namely sex of the animals (69% vs. 46%, p < 0.001), number of transgene copies (78% vs. 62%, p < 0.05) and genetic background (86% vs. 74%, p < 0.05). Reporting of measures to minimize bias was also higher for preclinical studies, namely random assignment of animals (46% vs. 6%, p < 0.001) and blinding of observers (44% vs. 24%, p < 0.001). Only 53% of all studies used animals of both sexes. Most preclinical studies (N = 71) used fewer ( 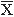 = 13.6; SD = 6.7) animals than the minimum of 24 recommended in the guidelines. No differences were found for these parameters between before and after the issuing of guidelines. Biases in animal data resulting from poor quality research prevent a fair assessment of the actual translational value of ALS models. Results must be reproducible in the same species before moving to humans, which can only be achieved if animal studies comply with best practice (e.g. through blinding and randomization). While our results suggest preclinical studies are more compliant with best practice than proof-of-concept studies, guidelines for ALS did not appear to have had an effect yet, suggesting more needs to be done to improve the planning, execution and reporting of mouse studies, a central requisite for making preclinical trials more reliable.

### CO40 – Effects of wine polyphenols in the neuroprotective role of transthyretin in alzheimer's disease

Luís Miguel Santos, MSc^1,2^, Daniela Rodrigues, BSc^1,2,3^, Mobina Alemi, MSc^1,2,4^, Carlos Alexandre Ribeiro, PhD^1,2^, Maria João Saraiva, PhD^1,2^, Nuno Mateus, PhD^5^, Isabel Cardoso, PhD^1,2^. ^1^IBMC – Institute for Molecular and Cellular Biology, Porto, Portugal, ^2^i3S – Instituto de Investigação e Inovação em Saúde (Institute for Innovation in Health Research), Porto, Portugal, ^3^ESTSP – School of Allied Health Sciences, Porto, Portugal, ^4^FMUP – Faculty of Medicine of University of Porto, Porto, Portugal, ^5^FCUP – Faculty of Sciences of University of Porto, Porto, Portugal (e-mail: luis.santos@ibmc.up.pt)

Wine polyphenols (PPs) (such as resveratrol, catechin, and malvidin) have been gaining importance in health and in their multiple biological effects, namely neuroprotective, as shown by their ability to attenuate Alzheimer's Disease (AD)-like symptoms in AD mouse models. Transthyretin (TTR), which binds Abeta (Aβ) peptide, avoiding its aggregation and toxicity, is decreased in AD patients, contributing to disease development. We previously showed that TTR tetrameric stabilizers improve TTR/Aβ affinity, leading to reduced Aβ brain and plasma levels and, consequently, to decreased peptide accumulation in the brain. Interestingly, resveratrol improves TTR/Aβ interaction *in vitro*, and its intake increases TTR protein levels in mice. Together, these results encouraged us to investigate the effect of red wine in AD, in particular of PPs and their relation with TTR, as an avenue for AD therapeutics. Firstly, we screened compounds for their ability to bind and to stabilize TTR, using a ^125^I-T_4_ binding assay and a TTR stability assay, respectively. Then, to assess TTR/Aβ binding in the presence of PPs, a competition assay was performed, showing that besides resveratrol, wine polyphenol extract was also able to improve TTR/Aβ binding, suggesting a synergistic effect of PPs. Importantly, resveratrol administrated to TTR/AD mice in their diet resulted in decreased amyloid plaque burden and Aβ_1-42_ brain levels, therefore confirming its benefits in AD. Furthermore, our results also indicated that resveratrol produces stronger effects when administrated in early stages of disease development, as deduced by the higher levels of Aβ_1-42_ in the formic acid-soluble fraction of the 10 months-old mouse, as compared to the 8-months old mice. Finally, we reproduced and optimized a cellular model for Aβ amyloid plaque formation using THP-1 differentiated cells. Here we showed that co-incubation with TTR resulted in reduced amyloid plaque number and size, and thus this model constitutes a valuable tool to screen compounds that enhance protection conferred by TTR in AD. In conclusion, our results confirm the benefits of some PPs, namely resveratrol, and prompt the use of combinatory administration of these compounds to produce synergistic enhanced neuroprotection.

**Acknowledgements:** to Fátima Macedo for THP-1 cells. This work was funded by national funds through FCT (Fundação para a Ciência e a Tecnologia)/MEC (Ministério da Educação e Ciência) and when applicable co-funded by FEDER funds within the partnership agreement PT2020 related with the research unit number 4293, and by FEDER funds through the Operational Competitiveness Programme– COMPETE and by National Funds through FCT –Fundação para a Ciência e a Tecnologia under the projects FCOMP-01-0124-FEDER-037277 (PEst-C/SAU/ LA0002/2013), FCOMP-01-0124-FEDER-020916 (PTDC/QUI-BIQ/118076/2010 and PTDC/SAU-ORG/116645/20109) and also by Fundació La Marató, Spain, through project 20140330-31-32-33-34. Cardoso I works under the Investigator FCT Program co-financed by the European Social Fund (ESF) through the Human Potential Operational Programme (HPOP), type 4.2 - Promotion of Scientific Employment. Santos LM is a recipient of a fellowship from OIV and Alemi M is a recipient of a fellowship (BIM) from IBMC funded by project of Fundació La Marató, Spain. Ribeiro CA was a recipient of a post-doc fellowship PEST-LA1101401-BPD.

### CO41 – Expression of inflammation-associated microRNAs in epilepsy – a preliminary study

Bárbara Leal, MSc^1,2^, Cláudia Carvalho,MSc^1,2^, Ricardo Ferreira, BSc^2^, João Chaves, MD^1,3^, Andreia Bettencourt, MSc^1,2^, Joel Freitas, MD^3^, João Lopes, MD^4^, João Ramalheira, MD^4^, António Martins da Silva, MD, PhD^1,4^, Paulo Pinho e Costa, MD, PhD^1,5^, Berta Martins da Silva, PhD^1,2^. ^1^Unit for Multidisciplinary Research in Biomedicine, Abel Salazar Institute of Biomedical Sciences, University of Porto – UMIB/ICBAS/UPorto. Rua Jorge Viterbo Ferreira, 228.4050-313, Porto, Portugal, ^2^Immunogenetics Laboratory, Abel Salazar Institute of Biomedical Sciences, University of Porto, ICBAS/UPorto. Rua Jorge Viterbo Ferreira, 228.4050-313, Porto, Portugal, ^3^Serviço Neurologia, Hospital Santo António – Centro Hospitalar do Porto. Largo Prof. Abel Salazar, 4099-001 Porto, Portugal, ^4^Serviço Neurofisiologia, Hospital Santo António – Centro Hospitalar do Porto. Largo Prof. Abel Salazar, 4099-001 Porto, Portugal, ^5^Instituto Nacional de Saúde Dr Ricardo Jorge (INSA), Delegacção do Porto. Rua Alexandre Herculano, 321, 4000-055 Porto, Portugal (e-mail: baguerraleal@gmail.com)

MicroRNAs (miRNAs) are emerging as new players in the epileptogenic mechanisms. MiRNA, small non-coding RNA molecules, function as post-transcriptional regulators of gene expression controlling diverse biological processes, including immune responses. Evidences, both in patients and animal studies, have demonstrated an abnormal brain expression of miR-146a and miR-155 in Mesial Temporal Lobe Epilepsy, the most refractory epilepsy type. Recently an overexpression of circulating miR-146a in patients with genetic generalized epilepsy (GGE), was also observed. Our aim was to characterize miR-146a and miR-155 expression in serum of MTLE and GGE patients. Expression levels of miR-146a, miR-155 and RNU48 (reference gene) were quantified by Real-Time PCR in serum of 34 patients (18 GGE and 16 MTLE). A group of 24 healthy individuals was used as control. Relative expression values were calculated using the 2-ΔΔCt method. The expression of miR-146a was similar in GGE patients when compared to controls but was 6 fold higher in MTLE patients. Expression of mir-155 was 3 fold lower in GGE patients but 2 fold higher in MTLE patients when compared to controls. Our results, although preliminary, show that miRs miR-146a and miR-155 may be suitable biomarkers for epileptogenesis. The different expression patterns in GGE and MTLE may reflect different pathways involved in seizures’ generation and propagation in these two epileptic syndromes. The comprehension of the role of these miRNAs in epilepsy's pathophysiological mechanisms may provide the development of new therapeutic strategies.

**Acknowledgements:** Financial Support by a BICE Tecnifar Grant 2013

### CO42 – MicroRNA-146a and Multiple Sclerosis

Andreia Bettencourt, MSc^1,2^, Rita Brás, BSc^2^, Cláudia Carvalho, MSc^1,2^, Bárbara Leal, MSc^1,2^, Raquel Samões, MD^3^, Ernestina Santos, MD^1,3^, Paulo Pinho e Costa, MD, PhD^1,4^, Ana Martins da Silva, MD, PhD^1,3^, Berta Martins da Silva, PhD^1,2,4^. Unit for Multidisciplinary Research in Biomedicine, Abel Salazar Institute of Biomedical Sciences, University of Porto – UMIB/ICBAS/UP, Porto, Portugal; Immunogenetics Laboratory, Abel Salazar Institute of Biomedical Sciences, University of Porto – ICBAS/UP, Porto, Portugal; Neurology Department, Centro Hospitalar do Porto- Hospital de Santo António, Porto, Portugal; Instituto Nacional de Saúde Dr Ricardo Jorge (INSA), Porto, Portugal (e-mail: ambettencourt@icbas.up.pt)

Multiple Sclerosis (MS) is an autoimmune, inflammatory neurodegenerative disorder of the central nervous system. MicroRNAs (miRNAs) are a class of small noncoding RNAs which have recently been discovered to be regulatory modulators of gene expression, controlling different biological processes, including immune responses. MicroRNA-146a (miRNA-146a) is considered an anti-inflammatory mediator as it represses NF-κB activity in various cell types, including monocytes, T cells and astrocytes. The aim of this study was to analyze the expression levels of circulating miRNA-146a in the serum of MS patients. The study included 43 MS patients (35 Relapsing-Remitting (RR) and 8 Secondary Progressive (SP)) from the outpatient Neuroimmunology Clinic of Centro Hospitalar do Porto – Hospital de Santo António - and 24 healthy controls (HC). All MS patients were in remission (no relapses within the previous 3 months). RNA extraction from serum samples was done using the miRNeasy Serum/Plasma Kit. MiRNA-146a gene expression was detected with a TaqManâ single miRNA assay. Relative expression values were calculated using the 2-ΔΔCt method. Results showed that the serum concentration of miRNA-146a was significantly lower in MS patients compared with HC (p = 0.002). No differences were found in the expression levels in RRMS as compared with SPMS. Circulating miRNAs have emerged as potential biomarkers for several human diseases including MS. Two previous studies have described an increased expression of miRNA-146a in peripheral mononuclear cells of RRMS patients. Another study reported that this miRNA was up regulated in active MS lesions. In this study we found decreased serum levels of miRNA-146a in MS patients. The discrepancy observed between this study and the ones already published are probably due a) to the type of biological sample analyzed (sera vs peripheral mononuclear cells) and b) the fact that in the present study all patients were in the remission phase, unlike in the other studies. Therefore, the reported up regulation of this miRNA, may reflect the acute phase of the disease, where inflammatory events are predominant.

### P27 – Apolipoprotein E polymorphisms and susceptibility to Genetic Generalized Epilepsies (confirmado em 2/11/2015)

Ricardo Ferreira, BSc^1^, Bárbara Leal, MSc^1,2^, João Chaves, MD^3^, Cláudia Carvalho, MSc^1,2^, Andreia Bettencourt, MSc^1,2^, Joel Freitas, MD^3^, João Lopes, MD^3^, João Ramalheira, MD^3^, Paulo Pinho e Costa, MD, PhD^1,2,4^, António Martins da Silva, MD, PhD^3^, Berta Martins da Silva, PhD^1,2^. ^1^Immunogenetics Laboratory, Abel Salazar Institute of Biomedical Sciences, University of Porto – ICBAS/UP, Porto, Portugal, ^2^Unit for Multidisciplinary Research in Biomedicine, Abel Salazar Institute of Biomedical Sciences, University of Porto – UMIB/ICBAS/UP, Porto, Portugal, ^3^Hospital de Santo António - Centro Hospitalar do Porto Porto, Portugal, ^4^Instituto Nacional de Saúde Dr Ricardo Jorge (INSA), Porto, Portugal (e-mail: baguerraleal@gmail.com)

Epilepsy is a chronic neurologic disorder that has a prevalence of 65 million people worldwide. Many factors such as neurotransmitters, voltage gated ionic channels, non-neural proliferation and inflammatory molecules have been suggested to be major players in epilepsy development. Apolipoprotein E (ApoE) is the main lipoprotein secreted in brain. It has a critical immunomodulatory function, influences neurotransmission and it is involved in repairing damaged neurons. ApoEε4 is an isoform of ApoE with altered protein function, previously associated with refractoriness and early onset epilepsy. These studies focused on animal models or patients with focal epilepsies. There is a limited knowledge on ApoE's role in Genetic Generalized Epilepsies (GGE). This study was undertaken to determine if ApoE isoforms are risk factors for GGEs. A group of 128 GGE patients (85F, 43 M, mean age = 29 ± 14 years) was studied and compared with a group of 342 healthy individuals in a case-control genetic association study. ApoE genotyping was performed with PCR-RFLP methodology. No differences in ApoE allelic frequencies between GGE patients and controls or between JME and controls were observed. The present results do not provide evidence that ApoE isoforms confer susceptibility to GGE at least in the studied population. This may be due to the limited sample size and consequent lack of statistical power to detect small genetic effects usual in complex polygenic disease such as GGE. To the best of our knowledge this is the first study on the role of ApoE in GGE susceptibility.

**Acknowledgements:** Financial Support by a BICE Tecnifar Grant 2013

## ONCOLOGY

### CO43 – Gastric cancer in the elderly: the impact of old age on gastrectomy

Isabel Mesquita, MD^1^, Carlos Nogueira, MD^1^, Jorge Santos, PhD^1^, Mário Marcos, MD^1^, Eduarda Santos, PhD^2^. ^1^Surgery Department, Hospital Santo António-Centro Hospital Porto, Oporto, Portugal, ^2^Instituto Biomédicas Abel Salazar, Oporto University, Oporto, Portugal (e-mail: mesquita.imm@gmail.com)

Old age is usually regarded as a risk factor for major abdominal surgery due to the presence of associated diseases and lack of functional reserve. Gastric cancer in the elderly represents, as we see it, an entity with specific characteristics and also specific therapeutic considerations and implications. This study aims to evaluate the impact of old age on surgical outcome when we propose a gastrectomy. We reviewed data on 880 patients (335 cases aged ≥ 70 years old defined as the elderly group, 545 patients aged < 69 years old) operated between January 1990 and December 2010. The parameters analyzed were demographic, comorbidities, surgical procedure, main postoperative complications and mortality. Statistical analysis was performed using IBM SPSS version Statistics 20 for Windows. A p value lower than 0.05 was considered statistically significant. In the last years we see, increased number of older people with gastric cancer, and also surgery is proposed more often. The ASA classification, comorbidities were higher in the elderly group (p < 0.001) particularly respiratory and cardiovascular. Elderly patients underwent more distal gastric resections than total gastrectomy (p < 0.001) and more D1 lymphadenectomy dissection than D2 dissection. The reconstruction procedure were mainly Roux-en-Y gastrojejunostomy or esophagojejunostomy. Univariate analyses showed that patient age was predictive for respiratory postoperative complications. The mortality rate was more elevated in the oldest group (p < 0.035). On the basis of our data and the results published, we may conclude that age should not be a limiting factor early ad for surgical treatment for gastric cancer. We can do as safe as for the younger patients the same procedures since no severe postoperative complication rate was observed with a serious impact on surgical outcomes.

### CO44 – Expression of iron-related proteins in lymphocytes and macrophages of the breast microenvironment

Oriana Marques, MSc^1,2,3,4^, Graça Porto, MD, PhD^2,3.4,5^, Alexandra Rêma, BSc^2^, Fátima Faria, BSc^2^, Arnaud Cruz Paula, MSc^2,6^, Maria Gomez-Lázaro, PhD^4,7^, Paula Silva, MsC^4,8,9^, Berta Martins da Silva, PhD^1,2^, Carlos Lopes, MD, PhD^2,6^. ^1^Unit for Multidisciplinary Biomedical Research (UMIB), Institute of Biomedical Sciences Abel Salazar (ICBAS), University of Porto, Porto, Portugal, ^2^Pathology and Molecular Immunology Department, Institute of Biomedical Sciences Abel Salazar (ICBAS), University of Porto, Porto, Portugal, ^3^Basic and Clinical Research on Iron Biology, Instituto de Biologia Molecular e Celular (IBMC), University of Porto, Porto, Portugal, ^4^Instituto de Investigação e Inovação em Saúde (i3S), University of Porto, Porto, Portugal, ^5^Hematology Service, Hospital de Santo António, Centro Hospitalar do Porto, Porto, Portugal, ^6^Department of Pathology, Portuguese Oncology Institute (IPO), Porto, Portugal, ^7^Instituto Nacional de Engenharia Biomédica (INEB), University of Porto, Porto, Portugal, ^8^Faculty of Medicine of University of Porto (FMUP), Porto, Portugal, ^9^Institute of Molecular Pathology and Immunology, University of Porto, Porto, Portugal (e-mail: oamarques@icbas.up.pt)

Despite critical advances in basic research, biomarkers and personalized therapy, breast cancer is still the leading cause of cancer death in women. Alterations in iron homeostasis have been frequently implicated in breast cancer development and progression. However, while the deregulation of iron homeostasis in breast epithelial cells is acknowledged, iron-related alterations in stromal inflammatory cells from the tumor microenvironment have not been explored. A total of 131 blocks from breast paraffin-embedded tissues (58 invasive ductal carcinomas, 16 ductal carcinomas in situ and 57 aesthetic surgery specimens) were prepared for tissue microarray construction and immunohistochemistry for the following iron-related proteins: hepcidin (HAMP), ferroportin 1 (FPN1), transferrin receptor 1 (TFR1) and ferritin (FT). Additionally, 26 paraffin-embedded axillary lymph node samples randomly chosen from the initial invasive ductal carcinoma cohort (14 non-metastized and 12 metastized) were submitted to the same immunohistochemical analysis in order to dissect the iron-profiles of epithelial cells, lymphocytes and macrophages in primary breast tissues and preferential metastatic niches. We confirm previously published results by showing that breast cancer epithelial cells present an ‘iron-utilization phenotype’ with an increased expression of HAMP and TFR1, and decreased expression of FT. On the other hand, lymphocytes and macrophages infiltrating primary tumors and from metastized lymph nodes display an ‘iron-donor’ phenotype, with increased expression of FPN1 and FT, concomitant with an activation profile reflected by a higher expression of TFR1 and HAMP. A higher percentage of breast carcinomas, as compared to control mastectomy samples, present iron accumulation in stromal inflammatory cells, suggesting that these cells may constitute an effective tissue iron reservoir. In addition, we found that not only the deregulated expression of iron-related proteins in epithelial cells, but also on lymphocytes and macrophages, are associated with clinicopathological markers of breast cancer poor prognosis, such as negative hormone receptor status in ductal carcinomas in situ and tumor size. Overall, the present results reinforce the importance of analyzing the tumor microenvironment in breast cancer, extending the contribution of immune cells to local iron homeostasis in the tumor microenvironment context.

**Acknowledgements:** OM and ACP acknowledge Fundação para a Ciência e Tecnologia (FCT, Portugal) for PhD grants.

### CO45 – Carcinogenic compounds in cooked meat: intake, bioavailability and mitigation strategies

Isabel M.P.L.V.O. Ferreira, PhD^1^, Olga Viegas, PhD ^1,2^, Armindo Melo, PhD ^1^, Miguel Faria, PhD ^1^, Olívia Pinho, PhD^1,2^. ^1^LAQV/REQUIMTE, Departamento de Ciências Químicas, Laboratório de Bromatologia e Hidrologia, Faculdade de Farmácia - Universidade do Porto, Portugal, ^2^Faculdade de Ciências da Nutrição e Alimentação - Universidade do Porto, Portugal (e-mail: isabel.ferreira@ff.up.pt)

The consumption of meat cooked at high temperatures, such as grilling, appears to confer an increased risk of a number of common cancers such as colorectal, pancreatic or prostate cancer (1). Most relevant carcinogenic compounds generated during meat cooking are heterocyclic aromatic amines (HAs) and polycyclic aromatic hydrocarbons (PAHs). Its formation in meat depends on type of meat, cooking method, time, and temperature, and addition of ingredients, thus mitigation strategies to reduce the intake of those carcinogenic compounds are needed to inform consumers about healthy practices. Additionally, *in vitro* studies about influence of diet patterns on bioacessibility and bioavailability of most representative HAs and PAHs, after simulated digestion is also of major relevance for risk assessment. The goals of this work were focused on (i) estimation of intake of HAs and PAHs from cooked meat (pan-fried and charcoal grilled beef, loin pork and chicken) by its quantification in meat samples cooked at home and taken from restaurants; (ii) search for cooking conditions that mitigate the formation of those carcinogenic compounds; (iii) investigate the stability of most relevant HAs and PAHs under gastro-intestinal conditions and their release from food matrix by simulation of an *in vitro* digestion; (iv) assess the influence of diet pattern on their absorption using an intestinal transport through a Caco-2 cell monolayer model. HAs and PAHs were quantified by high performance liquid chromatography/DAD. A standardised static *in vitro* digestion method was applied (2). Transport experiments were performed using Transwell inserts and a Caco-2 human colon cancer cell line (ATCC37-HTB). Results indicate that high amounts of HAs and PAHs can be taken in a single meal of charcoal grilled meat, ranging from 576 to 1898 and from 322 to 2497 ng/100 g of cooked meat, respectively. Keeping the meat away from the charcoal heat or using electrical griddle equipment mitigates the formation of HAs and PAHs. Meat marinating with dark beer before grilling can reduce 90% the formation of HAs and 53% the formation of PAHs. *In vitro* digestion indicate that the most abundant HA (PhIP) was less bioaccessible than the PAH representative bezo[a]pirene (BaP). The two different food matrices tested influenced the bioacessibility of BaP but had no significative effect in the transport through the Caco-2 monolayer.

**References:** (1) Zheng, W. & Lee, S. A. Nutr Cancer 2009, 61 (4), 437−446. (2) Minekus M. et al. Food Funct 2014, 5, 1113-1124

### CO46 – Fasting glucose but not metabolic syndrome or other components is frequently elevated in digestive neuroendocrine tumors

Santos Ana P, MSc^1^, Santos Ana C, PhD^2^, Castro C, MD^3^, Raposo L, MD^4^, Torres I, MD^1^, Henrique R PhD^5^, Monteiro MP, PhD^6^, Cardoso MH, PhD^7^. ^1^Dpt. of Endocrinology Dpt./Clinical Research Unit, Research Center of IPO-Porto, ^2^EPIUnit - Instituto de Saúde Pública da Universidade do Porto/Departamento de Epidemiologia Clínica, Medicina Preditiva e Saúde Pública da Faculdade de Medicina da Universidade do Porto, ^3^Dpt. of Epidemiology of IPO-Porto; 4 Dpt. of Endocrinology, IPO-Porto, ^2^EPIUnit - Instituto de Saúde Pública da Universidade do Porto, ^5^Dpt of Pathology of IPO-Porto, ^6^Clinical and Experimental Endocrinology, Unit for Multidisciplinary Research in Biomedicine, ICBAS, University of Porto, Portugal, ^7^Endocrinology, CHP/Clinical and Experimental Endocrinology, Unit for Multidisciplinary Research in Biomedicine, ICBAS, University of Porto. Porto, Portugal (e-mail: anapaulasantos@ipoporto.min-saude.pt)

Digestive neuroendocrine tumors (DNET) are rare neoplasms which incidence has increased 5-fold in the last 40y. Although occasionally can be familial, most are sporadic and their etiology is unknown. The link between visceral obesity, metabolic syndrome (MS) and cancer has been recently demonstrated for several types of neoplasia; however the association with NET has not so far been reported. To study the possible relationship between obesity, metabolic syndrome and NET. DNET tumors were classified according to localization, hormonal secretion, TNM stage and grading. Patients with DNET (n = 90) were cross matched with a control group (n = 90) of the general population derived from the PORMets study, according to age, gender and geographic location. Anthropometric and MS parameters according to JIS criteria were analyzed and compared between both groups. Biochemical assays were obtained prior to somatostatin analogues or PRRNT treatments. Statistical analysis was performed with SPSS 21.0; a level of significance of 0.05 was adopted. DNET patients had a mean age of 60.4 ± 12.1y with a slightly preponderance of males 5.3%. Tumors were either gastro-intestinal 46.7% (GI-NET) or pancreatic 24.4% (pNET), while 48.1% were functioning (45.2% carcinoid syndrome; 5.5% gastrinomas). According to grading, 70% were G1, 22.2% G2 and 1.1% NEC (grading unknown in 6.7%), yielding a prevalence of 35.5% of localized, 12.2% of locoregional and 46.7% of disseminated disease. Comparing DNET cases and controls, no differences were found in mean body weight (p = 0.816), body mass index (p = 0.216), waist circumference (p = 0.790) or obesity grade (p = 0.598). There were also no significant differences concerning the individual JIS criteria for hypertension (p = 0.999), triglycerides (p = 0.595); waist circumference (p = 0.999) and MS (p = 0.255). Only fasting glucose ≥ 100 mg/dL was significantly higher in DNET patients compared to controls (p = 0.013); independently of the localization of PT (p = 0.302), hormonal secretion (0.855), grading (p = 0.825) and staging (p = 0.601). Carbohydrate metabolism abnormalities, namely abnormal fasting glucose (FG) and type2 DM (T2-DM) were present in 56.5% of pts. Vs 25.8% of controls (p = 0.001). Patients with DNET have significantly higher frequency of carbohydrate abnormalities and fasting glucose levels above 100 mg/dL. These occur in DNETs independently from the localization of PT, hormonal secretion, WHO grading and extent of the disease, which suggests this finding is intrinsic to all DNETS and not only to pNETs.

### CO47 – The effects of endocannabinoids anandamide (AEA) and 2-arachidonoylglycerol (2-AG) in the endometrial cancer cell lines

BM Fonseca, PhD^1^, G Correia-da-Silva, PhD^1^, NA Teixeira, PhD^1^. ^1^UCIBIO, REQUIMTE, Laboratório de Bioquímica, Departamento Ciências Biológicas, Faculdade de Farmácia da Universidade do Porto, Porto, Portugal (e-mail: natercia@ff.up.pt, brunofonseca@ff.up.pt)

Endometrial cancer (EC) is the fourth most-prevalent cancer among women in Western Europe (1). Based on clinical and endocrine features, EC can occur in two forms: type 1 is the most common cancer that is classified as being estrogen-dependent, whereas type 2 is estrogen independent with a poorer prognosis (2). Recent evidence shows that derivatives of *Cannabis sativa* and its analogs may exert a protective effect against different types of oncologic pathologies. As the main active ingredient of cannabis, Δ9-tetrahydrocannabinol (Δ9-THC), endocannabinoids (eCBs) are bioactive lipids that have a range of interesting activities mediated by cannabinoid receptors (CB1 and CB2) (3). The two best characterized eCBs identified to date are anandamide (AEA) and 2-arachidonoylglycerol (2-AG), which are finely modulated under physiological and pathological conditions and elevated eCBs levels were reported in several types of tumors (4). Here we report the biochemical characterization of the endocannabinoid system in two cell lines representing type I and type II human EC: Ishikawa and Hec50co cells. We also examined the effects of eCBs in cell viability and morphology by MTT, LDH release, Giemsa and Hoescht staining. Both EC cell lines expresses several constituents of the endocannabinoid system including the CB1 receptor and the metabolic machinery responsible for synthesis and hydrolysis of endocannabinoids. Using the MTT assay, we showed that 10 μM of AEA treatment induced a significant reduction in cell viability in both Ishikawa and Hec50co cells. However, 2-AG only induced a reduction in cell viability of Hec50co cells for concentrations higher than 10 μM. These effects were demonstrated to be dose-dependent. We further demonstrate that AEA-induced cell death in Ishikawa cells resulted in significant increase in LDH release, though without significant effects on cell morphology. Moreover, it was observed cromathin condensation in Hec50co cells induced by both eCBs for concentrations higher than 10 μM as revealed by Hoescht staining. Data presented here indicates that EC cell lines express all the endocannabinoid system machinery and are sensitive to eCBs. Understanding the exact signaling by which eCBs regulate differently signaling pathways will eventually lead to targeted clinical approach.

Reference: 1. Torre LA, Bray F, Siegel RL, Ferlay J, Lortet-Tieulent J, Jemal A. Global cancer statistics, 2012. CA: a cancer journal for clinicians. 2015; 2. Murali R, Soslow RA, Weigelt B. Classification of endometrial carcinoma: more than two types. Lancet Oncol. 2014;15(7):E268-E78; 3. Fonseca BM, Costa MA, Almada M, Correia-da-Silva G, Teixeira NA. Endogenous cannabinoids revisited: a biochemistry perspective. Prostaglandins & other lipid mediators. 2013;102-103:13-30; 4. Hermanson DJ, Marnett LJ. Cannabinoids, endocannabinoids, and cancer. Cancer metastasis reviews. 2011;30(3-4):599-612.

Acknowledgments: This work was funded by Fundação para a Ciência e Tecnologia (FCT) under the project UID/Multi/04378/2013 and a post-doctoral grant (SFRH/BPD/72958/2010) to BM Fonseca.

### CO48 –Protein Profile in Human Prostate Cancer

Maria João Freitas, MSc^1^, Joana V Silva, MSc^1^, Juliana Felgueiras, MSc^1^, António Patrício, MD^2^, Nuno Maia, MD^2^, Steven Pelech, MD^3^, Margarida Fardilha, PhD^1^. ^1^Laboratory of Signal Transduction, Institute for Research in Biomedicine and Health Sciences Department, University of Aveiro, Campus de Santiago, 3810-193, Aveiro, Portugal, ^2^Infante D. Pedro Hospital, Av. Artur Ravara, 3814-501, Aveiro, Portugal, ^3^Kinexus Bioinformatics Corporation, Vancouver, British Columbia, Canada (e-mail: mfardilha@ua.pt)

Prostate cancer (PCa) incidence has been increasing in developed countries. In Portugal, PCa is the most common cancer and the third most deadly (behind lung cancer and colorectum) among men. Therefore, it is imperative to detect the disease and its malignancy at early and treatable stages. PCa diagnosis is mainly based on the quantification of the prostate specific antigen (PSA), but its sensitivity and specificity for diagnosis and prognosis is controversial. In fact, it has been suggested that PSA screening leads to unnecessary biopsies, overdetection and overtreatment. Hence, identifying specific molecules and signaling pathways involved in prostate carcinogenesis can pinpoint potential new biomarkers for PCa. This study aims to identify differentially expressed proteins that allow discrimination between normal and PCa tissues. Prostate biopsies from 8 patients (malignant and adjacent benign tissue) were pooled per group (normal and tumor). An antibody microarray was employed to analyze the expression and activation status of 800 signaling proteins in the two groups. To profile the proteins differently expressed between normal and tumor conditions, a bioinformatics analysis was performed: tissue expression, phenotype association, biological process, molecular function and cellular component. A comprehensive protein network was then built to identify the relations between the differentially expressed proteins. From the 40 proteins identified as differentially expressed between tumor and normal tissue, 17 were up-regulated, 23 were down-regulated and 13 presented altered activity in the tumor group. Interestingly, in tumor tissue, transcription factors and kinases suffer the most prominent alterations, being generally down-regulated and up-regulated, respectively. This study empowers the current knowledge on human PCa proteomics. Moreover, we identified potential new biomarkers, which may contribute to the improvement of PCa clinical management.

**Acknowledgements:** This work was supported by FEDER through the ‘Programa Operacional Fatores de Competitividade – COMPETE (FCOMP-01- 0124-FEDER-020895)’ and through national funds of ‘FCT – Fundação para a Ciência e Tecnologia to MF (PTDC/QUIBIQ/118492/2010), JVS (SFRH/BD/81458/2011) and MJF (SFRH/BD/84876/2012).

### CO49 – Estrogen-Dependent breast cancer: Autophagy as a potential mechanism of exemestane-acquired resistance

Cristina Amaral, PhD^1^, Georgina Correia-da-Siva, PhD^1^, Fernanda MF Roleira, PhD^2,3^, Elisiário Tavares da Silva, PhD^2,3^, Natércia Teixeira, PhD^1^. ^1^UCIBIO, REQUIMTE, Laboratory of Biochemistry, Department of Biological Sciences, Faculty of Pharmacy, University of Porto, Porto, Portugal, ^2^Pharmaceutical Chemistry Group, Faculty of Pharmacy, University of Coimbra, Coimbra, Portugal, ^3^CNC.IBILI, University of Coimbra, Coimbra, Portugal (e-mail: cristinamaralibd@gmail.com)

Aromatase inhibitors (AIs) is one of the therapeutic approaches used for estrogen-dependent (ER+) breast cancer. Despite their success, acquired resistance may occur after AI-treatment, causing tumor re-growth. Thus, AIs-resistance is the major drawback in endocrine therapy. Therefore, our group has been studying the effects of Exemestane (Exe), a third-generation steroidal AI used in clinic, in sensitive and resistant breast cancer cells in an attempt to find new targets to improve breast cancer treatment. We have previously shown that Exe induces apoptosis and autophagy in ER^+^ breast cancer cells (MCF-7aro), being autophagy a pro-survival process that protects cells from mitochondrial-mediated apoptosis (1). In order to understand the involvement of autophagy in Exe-acquired resistance, in this work it was investigated the effects of an autophagic inhibitor (3-methyladenine, 3-MA) in resistant breast cancer cells (LTEDaro) treated with Exe. It was evaluated the effects in cell viability, mitochondrial membrane potential (Δψm), caspases activities, ROS production and the formation of acid vesicular organelles (AVOs). The results indicate that Exe in LTEDaro cells increases the formation of AVOs, suggesting the occurrence of autophagy, as in sensitive cells. Although, Exe has no effects in LTEDaro cell viability, in Δψm, caspases activation and ROS production. In contrast, the addition of 3-MA to Exe-treated LTEDaro cells causes a reduction in cell viability, a decrease in AVOs formation, a loss of Δψm, activation of caspases-9, -8 and -7 and an increase in ROS production, effects similar to MCF-7aro cells. These results suggest that 3-MA sensitize LTEDaro cells to Exe by promoting cell death by apoptosis. Moreover, the autophagic inhibition in Exe-treated resistant cells enables cells to have a similar behavior as sensitive cells. So, autophagy besides being a pro-survival mechanism in Exe-treated ER^+^ breast cancer cells may also be a mechanism of Exe-acquired resistance. Therefore, the inhibition of autophagy may play a role in re-sensitize resistant cells to AIs and may have clinical benefits in breast cancer treatment. This work contributes to the understanding of the link between autophagy and AIs-resistance and will highlight new targets to improve breast cancer treatment.

**References:** (1) Amaral C., et al., 2012. “Apoptosis and autophagy in breast cancer cells following exemestane treatment.” PLoS One;7(8):e42398.

**Acknowledgements:** The authors are grateful to Fundação para a Ciência e Tecnologia (FCT) for the Post-Doc grant attributed to C. Amaral (SFRH/BPD/98304/2013) and to Dr. Shiuan Chen (Beckman Research Institute of the City of Hope, USA) for kindly supplying MCF-7aro and LTEDaro cells.

### CO50 – Excess adiposity correlates with the expression levels of MYC and PTEN on the mammary gland of a diet-induced obesity rat model

Begoña Cabia, MSc^1,2^, Sara Andrade MSc^1,2,4^, María Amil^1,2^, Marcos C Couselo PhD^1,2^, Felipe F Casanueva MD, PhD^1,2,3^, Ana B Crujeiras PhD^1,2^. ^1^Laboratory of Molecular Endocrinology, Instituto de Investigación Sanitaria (IDIS), Complejo Hospitalario Universitario de Santiago (CHUS), Santiago de Compostela, Spain, ^2^CIBER de Fisiopatología, Obesidad y Nutrición (CIBERobn), Madrid, Spain, ^3^Medicine Department, University of Santiago de Compostela, Spain, ^4^Anatomy Department, Instituto de Ciências Biomédicas Abel Salazar, University of Porto, Portugal (e-mail: begona.cabia@usc.es)

Obesity is increasing worldwide and is associated with higher risk for some tumors, such as breast cancer. Adipose tissue is an important endocrine organ that presents a dysfunctional production of several molecules in obese patients. Part of the research regarding this topic is focused on the role that factors produced by obese adipose tissue play on gene expression, as they can influence tumor development. The objective of this work was to study the association between excess body adiposity and the expression of PTEN, a tumor suppressor gene, and MYC, an important oncogene, on the mammary gland of Diet-Induced Obesity rat model. Three week-old female Sprague-Dawley rats were fed a high fat diet (DIO: 60% fat, n = 15) or standard diet (Lean: 5.5% fat, n = 5) for 10 weeks, with *ad libitum* access to food and water. Body weight and food intake were measured weekly. After sacrifice, retroperitoneal adipose tissue (RPAT) was weighted and mammary gland was extracted for qRT-PCR analysis of MYC and PTEN. The DIO group showed a body weight 9.61% higher than the Lean group (p = 0.005). These differences were reflected on RPAT content on the DIO (3.32 ± 0.23 g) vs. the Lean group (2.30 ± 0.21 g; p = 0.006). Relevantly, gene expression on the mammary gland of these animals for the oncogene MYC was positively associated with body weight (r = 0.52;p = 0.023), fat mass (r = 0.51;p = 0.044) and RPAT (r = 0.61;p = 0.012). On the contrary, the levels of the tumor suppressor gene PTEN were negatively correlated with body weight (r = –0.57;p = 0.014), fat mass (r = –0.65;p = 0.013) and RPAT (r = –0.71;p = 0.003). These results point out to a possible deregulation on the normal proliferative/antitumoral function on the mammary gland of obese animals caused by excess adiposity, and suggest a possible involvement of RPAT on these changes. Further studies are needed to identify the role of obesity-related carcinogenic factors on these alterations.

Acknowledgments: The present research was funded by CIBERobn, Xunta de Galicia, and Mapfre foundation.

### P28 – The protective effect of white tea extract against bladder cancer progression is accompanied by changes in the metabolic phenotype of cells

Luís R. Silva, PhD^1,2,3^, Marco G Alves, PhD^1^, Elsa Ramalhosa, PhD^4^, José A Pereira, PhD^4^, Pedro F Oliveira, PhD^1^, Branca M Silva, PhD^1^. ^1^CICS-UBI - Health Sciences Research Centre Faculty of Health Sciences, University of Beira Interior, Av. Infante D. Henrique, 6201-506, Covilhã, Portugal. ^2^LEPABE, Department of Chemistry Engineering, Faculty of Engineering, University of Porto, Rua Dr. Roberto Frias, s/n, 4200-465 Porto, Portugal. ^3^Institute Polytechnic of Castelo Branco, Higher School of Health Dr. Lopes Dias, Avenida do Empresário, Campus da Talagueira, 6000-767, Castelo Branco, Portugal, ^4^Mountain Research Centre (CIMO), School of Agriculture, Polytechnic Institute of Bragança, Portugal (e-mail: bmcms@ubi.pt)

Tea is a beverage obtained from the infusion of the leaves or buds of *Camellia sinensis* L. and is widely known for its anticancer properties. Several studies reported that green tea extract and some of its components may cause cell apoptosis, cell cycle arrest, modulate cell specific pathways and inhibit metastization processes in cancer, particularly in bladder cancer (1). We have recently reported that the progression from a lower to a higher invasive stage of bladder cancer is associated with severe alterations in glucose and pyruvate metabolism (1) and that white tea (WTEA) extract can modulate the glycolytic profile of cells (2). Thus, we hypothesized that a WTEA extract could alter the glycolytic profile of bladder cancer cells, modulating their proliferation. For that purpose, we studied two human bladder cancer cells, RT4 and TCCSUP, in which the latter represents a more invasive stage. The levels of glucose, pyruvate, alanine and lactate in the extracellular media were measured by Proton Nuclear Magnetic Resonance. The protein expression levels of key glycolysis-related enzymes and transporters were determined. Cytotoxic studies revealed that 0.25 mg/ml and 1 mg/ml of WTEA extract successfully induced cell death in the primitive cancer stage, but proliferation arrest on TCCSUP cell line was only achieved by 1 mg/ml WT extract. This was associated with alterations in the glycolytic profile of bladder cancer cells. WTEA extract stimulates glucose consumption and lactate production in RT4 cells while decreases glucose consumption by TCCSUP. The phosphofructokinase-1 and monocarboxylate-4 expression in TCCSUP cells were modulated by exposure to the highest concentration of WTEA extract. Notably, pyruvate metabolism was reverted by exposure to WTEA in RT4 cells. Our work demonstrates that bladder cancer progression includes several alterations in the cells’ metabolism, from which pyruvate consumption seems to be a major factor. Notably, exposure to a WTEA extract successfully induces cell death in primitive and more advanced bladder cancer stages. This is accompanied by changes in cells metabolic phenotype. The mechanisms reported herein present new insights regarding WTEA anticancer properties and mechanisms of action which may be of extreme importance for the future development of new therapeutic strategies for bladder cancer.

**References:** (1) Conde VR et al (2015), Experimental Cell Research, 2015, 335(1): 91-98.

(2) Martins AD et al (2015), European Journal of Nutrition, 2014, 53(6):1383-1391.

Acknowledgments: This work was funded by FCT to CICS (PEst-OE/SAU/UI0709/2014); LRS (SFRH/BPD/105263/2014); MGA (SFRH/BPD/80451/2011) and PFO (PTDC/QUIBIQ/121446/2010).

### P29 – Ferritin expression in breast cancer infiltrating lymphocytes

Leite, Luciana, BSc^1^, Marques, Oriana, MSc^1,2,3,4^, Rosa, Ana, BSc^1,3,4^, Rêma, Alexandra, BSc^1^, Faria, Fátima, BSc^1^, Porto, Graça, MD, PhD^1,3,4,5^, Cruz Paula, Arnaud, MSc^2,6^, Lopes, Carlos, MD, PhD^1,6^, Martins da Silva, Berta, PhD^1,2^. ^1^Pathology and Molecular Immunology Department, Institute of Biomedical Sciences Abel Salazar (ICBAS), University of Porto, Porto, Portugal, ^2^Unit for Multidisciplinary Biomedical Research (UMIB), Institute of Biomedical Sciences Abel Salazar (ICBAS), University of Porto, Porto, Portugal, ^3^Basic and Clinical Research on Iron Biology, Instituto de Biologia Molecular e Celular (IBMC), University of Porto, Porto, Portugal, ^4^Instituto de Investigação e Inovação em Saúde (i3S), University of Porto, Porto, Portugal, ^5^Hematology Service, Hospital de Santo António, Centro Hospitalar do Porto, Porto, Portugal, ^6^Department of Pathology, Portuguese Oncology Institute (IPO), Porto, Portugal (e-mail: lucianareisleite@gmail.com)

Breast cancer development and progression are associated with a deregulation of iron homeostasis, as revealed by differences in the expression of several iron-related proteins. One of such proteins is ferritin, whose increased tissue levels have been consistently associated with breast cancer risk, severity and recurrence. Previous studies demonstrated that breast cancer infiltrating macrophages secrete mitogenic ferritin that stimulates the proliferation of breast cancer epithelial cells independently of its iron content. Our group has demonstrated that ferritin synthesis is also increased in breast cancer infiltrating lymphocytes. Accumulating evidence suggested that ferritin secretion could also be associated with the HLA-A^∗^03 allele and HFE polymorphisms. The main objective of this project was to verify if a certain immune-profile was associated with an increased expression of ferritin in breast cancer infiltrating lymphocytes. Additionally, we analyzed if ferritin expression in breast cancer epithelial cells and lymphocytes was associated with clinicopathological variables of breast cancer progression and behaviour. Ferritin expression in breast epithelial cells and lymphocytes, and total CD4, CD8 and CD4+FoxP3+ T-cell numbers were assessed by immunohistochemistry in a total of 134 samples from tissue microarray blocks. The median ferritin expression in the epithelium and lymphocytic infiltrate was evaluated by a semi-quantitative method, considering the stained area and its intensity. The median number of total lymphocytes was assessed in 5 High-Power Fields (400x). Hemosiderin deposits in epithelial and stromal inflammatory cells were detected with the DAB-enhanced Perls’ staining method. DNA extraction was performed from Formalin Fixed, Paraffin-embedded (FFPE) blocks and/or peripheral blood. HFE polymorphisms (C282Y and H63D) and HLA-A^∗^03 genotyping were evaluated by Polymerase Chain Reaction (PCR). We confirmed that median ferritin expression was decreased in epithelial cells from carcinoma samples, but increased in infiltrating lymphocytes. These carcinoma samples were characterized by higher median numbers of CD4+, CD8+ and CD4+FoxP3+T-cells. Surprisingly, the proportion of CD4/CD8 T-cells was not associated with an increased ferritin expression in lymphocytes. However, the FoxP3/CD4 ratio was positively correlated with the median ferritin expression in lymphocytes. In stromal inflammatory cells, the presence of hemosiderin deposits was associated with the median ferritin expression in epithelial cells and not in lymphocytes. In relation to clinicopathological variables, invasive ductal carcinoma (IDC) estrogen receptor (ER) positive cases presented a significantly higher median ferritin. In ductal carcinoma in situ (DCIS) samples, the median number of total lymphocytes was significantly higher in hormone receptor negative cases. CD4+ T-lymphocyte median numbers were significantly higher in ER negative, progesterone receptor (PR) negative and Human Epidermal Growth Factor 2 (HER-2) positive cases, in DCIS samples. A higher median number of CD8+ and CD4+FoxP3+T-cells was observed in ER negative DCIS cases. Our group has previously demonstrated that macrophages and lymphocytes present an “iron-donor” phenotype, as observed by its higher ferroportin 1 (Fpn1) expression in breast cancer tissue. However, evidences from other studies suggest that ferritin secretion, particularly by macrophages, may constitute an alternative route of iron delivery. The fact that ferritin expression in lymphocytes was not correlated with iron accumulation in stromal inflammatory cells may indicate a similar mechanism, not associated with ferritin's classical role as an iron storage protein. In fact, the “iron-donor” phenotype of tumor-infiltrating lymphocytes may play an important role in the tumor microenvironment, through the local regulation of iron homeostasis and potentially contributing to tumor nutrition.

### P30 – Phosphoprotein phosphatase 1 interactome in human prostate cancer: a myriad of potential therapeutic opportunities

Juliana Felgueiras, MSc^1^, Joana V Silva, MSc^1^, Margarida Fardilha, PhD^1^. ^1^Laboratory of Signal Transduction, Institute for Research in Biomedicine – iBiMED, Health Sciences Program, University of Aveiro, Campus Universitário de Santiago, 3810-193 Aveiro, Portugal (e-mail: mfardilha@ua.pt)

The complex protein-protein interaction network that governs normal prostate morphology and physiology is disrupted during prostate cancer (PCa) to fulfil cancer cells metabolic requirements. Alterations in protein phosphorylation are particularly relevant since they command the activation state of several proteins and regulate cancer-associated pathways. Phosphoprotein phosphatase 1 (PPP1) is a major Ser/Thr phosphatase that may exhibit both oncogene and tumour suppressor roles depending on its interacting proteins (PIPs). PPP1-PIPs complexes represent challenging therapeutic targets since they are more cell type- and context-specific than PPP1 itself. As far as we know, only 4 PPP1-PIPs complexes were hitherto characterized in human PCa models, but many more may remain unknown. Here, we identified PPP1 complexes potentially relevant in human prostate carcinogenesis using a bioinformatics approach. PIPs were retrieved from the HIPPIE database (access date: 30/06/2015). Tissue databases (e.g. TissueNet, BioGPS, HPA) were explored to determine their expression in human prostate tissue. Additional bioinformatics tools were used to analyse the biological relevance of PIPs in cancer- and prostate-related phenotypes (MGI database), pathways (KEGG database) and biological processes (AmiGO Term Enrichment tool). The bioinformatics search was complemented by a comprehensive literature search. The HIPPIE search revealed 315, 98 and 252 PIPs for the PPP1 isoforms (PPP1CA, PPP1CB and PPP1CC, respectively). None of the PIPs are prostate-specific, but at least 28 are highly expressed in prostate tissue. The knockout of several PIPs in mice leads to cancer-related phenotypes, but ESR1, PTEN and SCRIB knockouts are specifically associated with altered prostate morphology and/or physiology. Regarding PIPs involvement in cell signalling, “Pathways in cancer” appears within the top-5 results for the 3 PPP1 isoforms and “Prostate cancer” pathway seems to be particularly enriched. PIPs involved in “Prostate cancer” signalling include, for example, TP53, RB1, AKT1 and CCND1. PPP1CA/CB interactors are primarily involved in cell proliferation and differentiation, while PPP1CC interactors are mainly involved in cell proliferation and migration. In this study, we highlight a number of PPP1-PIPs complexes whose involvement in prostate carcinogenesis merits further elucidation in order to explore them as new potential therapeutic targets.

**Acknowledgements:** This work was supported by individual grant from FCT to JF (SFRH/BD/102981/2014).

## CONCLUDING REMARKS

Artur P Águas. Abel Salazar Institute of Biomedical Sciences, University of Porto, Portugal

“In my lifetime, the most important discovery in diabetes treatment was announced a couple of weeks ago at the European Association for the Study of Diabetes (EASD) meeting that I have attended last month!” Those were the words chosen by Jens Holst (Copenhagen) to begin his lecture at the second day of the UMIB Summit that took place in Porto in September 24/25, 2015. This episode illustrates the spirit of the meeting: renowned scientists offering their personal views on the latest advances in fundamental and clinical medicine. They were followed by lively discussions with an audience that filled the largest lecture hall of the Biomedical School of the University of Porto. The UMIB Summit was focused on issues of translational medicine of our day, e. g., dendritic cells and their ever growing role in immune surveillance and inflammation (Andrew Macdonald, Manchester), how exosomes may be useful in cancer treatment (Christian Rolfo, Antwerp), or on the changing concepts regarding late-onset hypogonadism (Ilpo Huhtaniemi, London). All sessions had lectures by Portuguese researchers and scientists coming from other European countries. Mariana P. Monteiro, the director of UMIB, the host institution, presented the latest discoveries on the so-called “surgery of diabetes” and how gastrointestinal hormones abrogate diabetes after bariatric surgery. Illuminating lessons were also presented on the difficulties and challenges to establish units for translational research within hospitals (Immaculada Cáceres, Madrid), and on how to participate in the EuroBioBank (Marina Mora, Milano). Poster presentations by young scientists fostered the exchange of experience and original data among the several hundred participants coming from all over Europe, and also from other continents. The UMIB Summit was a great success that deserves to be tried again!

